# Interactome Analyses Identify Ties of PrP^C^ and Its Mammalian Paralogs to Oligomannosidic N-Glycans and Endoplasmic Reticulum-Derived Chaperones

**DOI:** 10.1371/journal.ppat.1000608

**Published:** 2009-10-02

**Authors:** Joel C. Watts, Hairu Huo, Yu Bai, Sepehr Ehsani, Amy Hye Won, Tujin Shi, Nathalie Daude, Agnes Lau, Rebecca Young, Lei Xu, George A. Carlson, David Williams, David Westaway, Gerold Schmitt-Ulms

**Affiliations:** 1 Centre for Research in Neurodegenerative Diseases, University of Toronto, Toronto, Ontario, Canada; 2 Department of Laboratory Medicine and Pathobiology, University of Toronto, Toronto, Ontario, Canada; 3 Alberta Centre for Prions and Protein Folding Diseases, University of Alberta, Edmonton, Alberta, Canada; 4 McLaughlin Research Institute, Great Falls, Montana, United States of America; 5 Department of Biochemistry, University of Toronto, Toronto, Ontario, Canada; University of Edinburgh, United Kingdom

## Abstract

The physiological environment which hosts the conformational conversion of the cellular prion protein (PrP^C^) to disease-associated isoforms has remained enigmatic. A quantitative investigation of the PrP^C^ interactome was conducted in a cell culture model permissive to prion replication. To facilitate recognition of relevant interactors, the study was extended to Doppel (Prnd) and Shadoo (Sprn), two mammalian PrP^C^ paralogs. Interestingly, this work not only established a similar physiological environment for the three prion protein family members in neuroblastoma cells, but also suggested direct interactions amongst them. Furthermore, multiple interactions between PrP^C^ and the neural cell adhesion molecule, the laminin receptor precursor, Na/K ATPases and protein disulfide isomerases (PDI) were confirmed, thereby reconciling previously separate findings. Subsequent validation experiments established that interactions of PrP^C^ with PDIs may extend beyond the endoplasmic reticulum and may play a hitherto unrecognized role in the accumulation of PrP^Sc^. A simple hypothesis is presented which accounts for the majority of interactions observed in uninfected cells and suggests that PrP^C^ organizes its molecular environment on account of its ability to bind to adhesion molecules harboring immunoglobulin-like domains, which in turn recognize oligomannose-bearing membrane proteins.

## Author Summary

Prions underlie rare but invariably fatal neurodegenerative diseases in humans and other mammals. Public awareness of these diseases has grown with the occurrence of cases of BSE (also known as “Mad Cow Disease”) and the realization that this disease can, in rare instances, be transmitted to humans. The normal cellular prion protein is found in most cell types within the body. In disease, this protein acquires a different shape which tends to aggregate and poison nearby cells. The disease-associated conversion of the prion protein appears to require its localization to specialized cellular membrane regions rich in cholesterol; however, the precise molecular environment which hosts this event has remained elusive. We used a cell-based disease model to identify proteins which reside in close proximity to the prion protein and two closely related mammalian proteins. We demonstrate that these three proteins may not only populate highly similar environments but may also interact with each other. We further identified an extended molecular network in proximity of these proteins which supports functions in adhesion control, lactate metabolism and cell fusion events. It is anticipated that these insights will contribute to efforts to rationally interfere with aberrant protein-protein interactions underlying these diseases.

## Introduction

Prions are the causative agents underlying a range of rare but invariably fatal neurodegenerative diseases in humans and other mammals. In disease, the cellular prion protein (Prnp; herein referred to as PrP^C^) converts to a disease-associated conformer (PrP^Sc^) with different physicochemical properties [1]. PrP^C^ is a relatively small protein which assembles into an unstructured N-terminal domain and a globular C-terminal half characterized by the presence of an internal disulfide bridge, up to two N-linked glycans and a glycosylphosphatidylinositol (GPI) anchor for insertion into the cellular plasma membrane. Multiple lines of investigation have led to the conclusion that mature PrP^C^ is embedded in specialized membrane domains, so-called raft-like domains, rich in cholesterol and sphingolipids [2]. It has been suggested that these raft-like domains host the self-perpetuated accumulation of PrP^Sc^ which subsequently triggers a poorly-understood cascade of events that ultimately leads to cell death. Whereas significant progress has been made in the past few years in defining the minimal requirements for PrP^C^ conversion *in vitro* [3], the molecular environment which hosts the earliest steps in prion disease manifestation in neuronal cells remains enigmatic. This shortcoming is not due to a lack of proteins proposed to interact directly with PrP^C^. In fact, more than three dozen proteins have been suggested to reside in spatial proximity to PrP^C^ using multiple experimental paradigms [4]. Additional candidate interactors have been proposed to bind preferentially to PrP^Sc^ [5],[6],[7]. In surveying this body of literature, however, it is apparent that very few of the candidate interactors have been independently verified by multiple investigators and, overall, little agreement exists as to their relative importance for prion protein biology. Notable exceptions may represent the 37-kDa/67-kDa laminin receptor precursor (herein referred to as LRP; also known as ribosomal protein SA (Rpsa), and not to be confused with Lrp1, the low density lipoprotein receptor-related protein 1) [8], one of the first proteins identified to bind to the prion protein in a yeast two-hybrid (Y2H) screen [9]; the neural cell adhesion molecule (Ncam1; herein referred to as NCAM), which was initially identified in a cellular crosslinking study based on a co-immunoprecipitation methodology [10]; and heparin sulfate proteoglycans (HSPGs) [11],[12]. Since its original discovery, LRP has been proposed to act as a cell surface receptor for PrP^C^ that may play a role in prion propagation [13]. In the case of NCAM, binding to PrP has been shown to play a role in neuritic outgrowth possibly mediated through an interaction with Fyn tyrosine kinase [14],[15].

In studying the molecular environment of membrane proteins, a limitation exists in that the proteins must be solubilized by the addition of detergents in a manner that does not disrupt the protein-protein interactions under investigation. A solution to this obstacle constitutes the covalent stabilization of interactions by chemical crosslinking prior to the disruption of cellular integrity. In this regard, we previously reported on a large-scale investigation of the molecular PrP^C^ neighborhood in mice following limited *in vivo* crosslinking by time-controlled transcardiac perfusion (tcTPC)-based delivery of formaldehyde (FA) to the brain [16]. A conspicuous feature of this PrP^C^ interactome dataset was the relative abundance of membrane proteins which harbor immunoglobulin (Ig)-like folding motifs in their extracellular domains confirming previous data describing a binding domain of PrP^C^ within these folds [10].

Given the large diversity of cell types present in the brain, we questioned whether an equivalent concentration of Ig-like domain-harboring proteins would have been found if the investigation of the PrP^C^ interactome had been restricted to a single cell type. A particular concern was that the relative abundance of Ig-like domain-harboring proteins in the previous dataset may have masked the ability to identify biologically important interactions of PrP^C^ that are more transient in nature or involve less abundant proteins. We therefore chose to reinvestigate the PrP^C^ interactome in mouse neuroblastoma cells (N2a), which are by far the best-characterized cell model for the study of the biology and conversion of PrP^C^ to PrP^Sc^ [17],[18]. To facilitate the discrimination of unspecific binders from specific interactors in this study, we incorporated quantitative mass spectrometry based on isotopic labeling and extended our investigation to the mammalian PrP^C^ paralogs Doppel (encoded by the *Prnd* gene; protein product herein referred to as Dpl) and Shadoo (encoded by the *Sprn* gene; protein product herein referred to as Sho). To avoid the use of non-identical affinity chromatography steps, we equipped the three bait proteins with the same N-terminal FLAG epitope [19], with the awareness that a similar PrP^C^ expression construct had in prior investigations neither interfered with the posttranslational processing nor the conversion of PrP^C^ in cell or animal models [18]. We now present data which suggest that members of the mammalian prion protein family may populate highly similar molecular environments when expressed in the neuroblastoma cell model system. We further document that a subset of endoplasmic reticulum (ER) chaperones which interact strongly with PrP^C^ escape from the ER to reside in spatial proximity to PrP^C^ at the plasma membrane. Pharmacological inhibition of these chaperones could increase PrP^Sc^ levels in prion-infected N2a sublines, suggesting a protective role for the chaperones in this paradigm. Finally, our data consolidate multiple previously controversial interactions and suggest a scenario whereby PrP^C^ may organize its molecular environment by its ability to recognize a specialized subset of cell adhesion molecules which recruit membrane proteins carrying high-mannose glycans into spatial proximity with PrP^C^.

## Results

### Large-scale quantitative and comparative interactome investigation of members of the mammalian prion protein family

**Figure 1:**
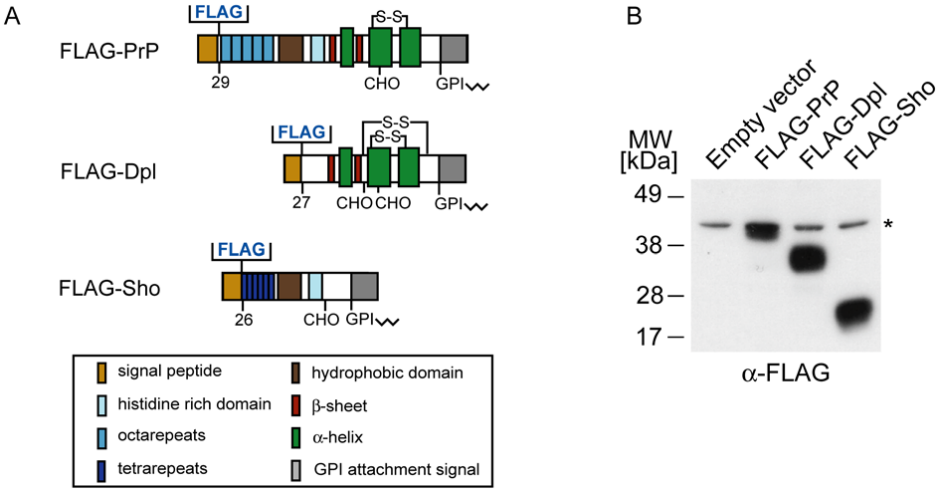
Expression analysis of FLAG-tagged mouse prion proteins. A, Schematic representation of murine prion proteins with FLAG tags inserted near the N-terminus. B, Expression of transiently-transfected FLAG-prion proteins in N2a cells as assessed by Western blotting with the anti-FLAG M2 antibody. The presence of a non-specific band in N2a lysates recognized by the M2 antibody is denoted by an asterisk.

**Figure 2:**
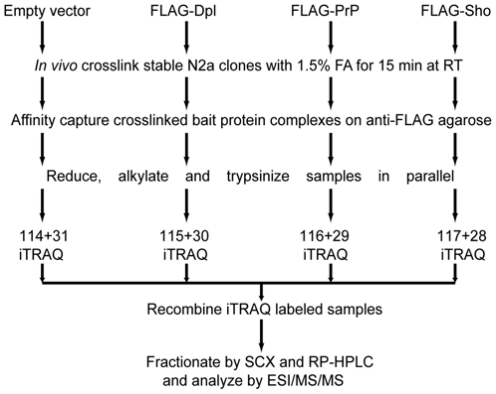
Flow chart depicting strategy for semi-quantitative comparison of prion protein family interactomes. *In vivo* formaldehyde crosslinked protein complexes containing N-terminally FLAG-tagged bait proteins are stringently purified on anti-FLAG agarose parallel to a negative control sample derived from an empty vector expression clone. Following alkylation, reduction and trypsinization, digests are side-by-side iTRAQ labeled and subsequently combined. Two-dimensional liquid chromatography of peptides is coupled to online ESI-MS/MS, which is followed by computationally-aided protein identification and quantitative analysis.

In preparation for this study, mouse neuroblastoma cells (N2a) stably expressing N-terminally FLAG-tagged full-length versions of Dpl, PrP and Sho were generated (Fig. 1). Small-scale expression tests followed by diagnostic N-glycosidase F or phosphatidylinositol-specific phospholipase C digestions confirmed that all three bait proteins mimicked their untagged parent molecules in (i) the presence of N-glycans, and (ii) membrane attachment by means of a GPI anchor [20] (and data not shown). To generate the biological source material for a large-scale comparative interactome investigation, the three cell lines and an “empty-vector” stably-transfected N2a cell line serving as a negative control were expanded to 10^9^ cells each using cell culture conditions which promote adherent growth. To covalently stabilize protein-protein interactions prior to the disruption of cellular integrity, cells were subjected to a 15-min treatment with FA [10]. Subsequently, cells were lysed by the addition of detergents and the four extracts (each containing approximately 500 mg of cellular protein) purified side-by-side on anti-FLAG affinity agarose matrices. The presence of covalent linkages between proteins permitted the use of highly stringent washing conditions to minimize the presence of unspecific binders. Following elution from the affinity matrix (with a yield of approximately 100 µg of protein material per sample), protein complexes were denatured and trypsinized. Finally, peptide mixtures were tagged with isobaric tags for relative and absolute quantitation (iTRAQ) [21], and the samples were combined and subjected to a comprehensive analysis by tandem mass spectrometry (MS/MS) (Fig. 2). A query of mouse protein databases led to the identification of more than 100 proteins. All identifications can be considered confident by multiple measures: (i) identical identifications were made by two matching algorithms; (ii) scores assigned by the algorithms exceeded significance thresholds (*P*<0.05) for all identifications and were based on a minimum of two peptides; and (iii) a search of a decoy database generated by the inversion of sequences for all mouse protein entries resulted in no identifications which shared any of the above features [22]. A reduction of the total list of identified proteins to the subset of proteins whose identification correlated with the presence of at least one of the three bait proteins was based on peak intensities of iTRAQ reporter ions found in the low mass range of individual collision induced dissociation (CID) spectra. The relative intensity of these ions is indicative of the relative contribution of each of the iTRAQ-labeled samples to the generation of a given CID spectrum. Thus, by calculating the ratio of reporter ion intensities for each of the three bait-specific reporter ions (iTRAQ 115: Dpl; iTRAQ 116: PrP; iTRAQ 117: Sho) and the negative control ion (iTRAQ 114 reporter), peptides purifying with at least one of the three baits were recognized by iTRAQ reporter ion ratios greater than 1. The above analysis revealed that more than 50 proteins co-purified with at least one of the three bait proteins (Table 1). Interestingly, the majority of these proteins co-purified with all three bait proteins, suggesting that the molecular environment of the three members of the mammalian prion protein family assessed in the context of mouse neuroblastoma cells is highly similar.

**Table 1:** Prion protein family interactome in mouse neuroblastoma cells.

IPI accession number	Symbol	Identified proteins^a^	Pept.^b^	Unique^c^	% Cov.^d^	114	115	116	117
						Control^e^	Dpl	PrP	Sho
IPI:IPI00131622.1	Prnd	prion protein dublet (doppel, Dpl)	8	13	39.1	2.0	92.5	3.0	2.5
IPI:IPI00120793.1	Prnp	prion protein (PrP)	5	5	26.8	4.0	20.5	65.1	10.5
IPI:IPI00226455.1	Sprn	shadow of prion protein (shadoo, Sho)	3	6	69.4	2.9	11.4	20.5	65.2
IPI:IPI00751369.1	Ldha	lactate dehydrogenase A	15	19	67.3	6.0	26.3	44.0	23.7
IPI:IPI00230108.6	Pdia3	protein disulfide isomerase associated 3	14	15	61.2	6.0	46.7	18.8	28.4
IPI:IPI00229517.5	Lgals1	galectin-1 (lectin, galactose binding, soluble 1)	5	7	57.8	5.3	32.8	37.4	24.4
IPI:IPI00122971.1	Ncam1	neural cell adhesion molecule 1 (NCAM)	23	34	54.3	3.4	58.6	16.6	21.3
IPI:IPI00670985.3	Gm9234	predicted gene 9234 (EG668548)	6	6	53.7	13.6	29.8	36.7	20.0
IPI:IPI00128973.1	Gap43	growth associated protein 43 (neuromodulin)	2	2	46.7	7.0	30.5	24.5	38.0
IPI:IPI00850840.1	Rpsa	ribosomal protein SA (laminin receptor precursor, LRP)	7	8	43.4	11.8	33.8	26.9	27.5
IPI:IPI00319992.1	Hspa5	heat shock protein 5	15	20	42.7	7.4	32.9	22.5	37.3
IPI:IPI00515173.1	H2-K1	histocompatibility 2, K1, K region	6	6	40.6	3.6	60.9	17.0	18.5
IPI:IPI00762203.2	Ftl1	ferritin light chain 1	3	4	39.9	10.4	36.9	29.4	23.3
IPI:IPI00454042.2	Fam3c	family with sequence similarity 3, member C	4	4	35.7	4.7	64.4	12.5	18.5
IPI:IPI00133522.1	P4hb	protein disulfide-isomerase (prolyl 4-hydroxylase beta)	8	9	35.4	5.7	22.5	28.4	43.4
IPI:IPI00135686.2	Ppib	peptidylprolyl isomerase B	4	4	34.3	10.3	30.3	24.2	35.1
IPI:IPI00132950.1	Rps21	ribosomal protein S21	2	2	31.3	8.6	18.7	37.9	34.9
IPI:IPI00110805.1	H2-D1	histocompatibility 2, D region locus 1	5	5	29.3	3.4	56.7	19.9	20.0
IPI:IPI00119618.1	Canx	calnexin precursor	8	8	28.8	7.1	40.7	18.8	33.4
IPI:IPI00554929.2	Hsp90ab1	heat shock protein 90 alpha (cytosolic), class B member 1	10	11	28.6	11.5	15.6	33.4	39.6
IPI:IPI00123639.1	Calr	calreticulin	3	3	27.9	7.2	32.5	24.7	35.6
IPI:IPI00116498.1	Ywhaz	14-3-3 protein zeta	4	5	27.8	10.0	21.6	35.4	33.0
IPI:IPI00462199.1	Bsg	basigin	5	5	27.3	4.1	32.0	23.2	40.7
IPI:IPI00230682.6	Ywhab	14-3-3 protein beta	2	2	27.0	10.5	26.3	23.4	39.8
IPI:IPI00857709.1	Tmed2	transmembrane emp24 domain trafficking protein 2	2	2	26.9	6.0	33.6	26.1	34.4
IPI:IPI00854971.1	Pdia6	protein disulfide isomerase associated 6	2	2	25.6	7.8	29.5	27.9	34.9
IPI:IPI00466570.4	Tmed10	transmembrane emp24-like trafficking protein 10	4	5	25.1	3.5	35.7	16.8	44.0
IPI:IPI00230707.6	Ywhag	14-3-3 protein gamma	2	2	23.9	9.8	12.0	48.7	29.6
IPI:IPI00132799.4	C1qbp	complement component 1, q subcomponent binding protein	4	4	23.3	9.8	17.4	22.2	50.7
IPI:IPI00124700.1	Tfrc	transferrin receptor protein 1	5	5	22.4	6.6	49.4	19.1	24.9
IPI:IPI00129526.1	Hsp90b1	heat shock protein 90, beta (Grp94), member 1 (endoplasmin)	3	3	20.9	7.7	23.4	28.7	40.2
IPI:IPI00123342.4	Hyou1	hypoxia up-regulated 1	2	2	20.6	10.4	25.3	31.4	32.9
IPI:IPI00762435.2	Fyn	fyn proto-oncogene	3	3	20.5	13.3	24.2	28.6	33.9
IPI:IPI00129519.3	Basp1	brain abundant, membrane attached signal protein 1	7	10	19.9	8.2	18.8	37.4	35.5
IPI:IPI00187289.2	Tmem206	transmembrane protein 206 (C1orf75)	2	2	18.9	3.5	18.2	11.1	67.2
IPI:IPI00416577.1	Gdi2	guanosine diphosphate (GDP) dissociation inhibitor 2	2	2	18.8	6.3	32.8	28.0	33.0
IPI:IPI00132474.3	Itgb1	integrin beta 1	5	5	18.4	7.1	33.3	28.1	31.6
IPI:IPI00265291.6	Enpp1	ectonucleotide pyrophosphatase/phosphodiesterase 1	4	4	18.3	9.5	37.6	24.2	28.7
IPI:IPI00114641.2	Slc3a2	solute carrier family 3, member 2 (CD98)	2	4	18.2	10.2	43.6	21.4	24.8
IPI:IPI00420835.3	Itga6	integrin alpha 6	3	3	15.6	8.1	40.2	20.6	31.1
IPI:IPI00762897.1	Ugcgl1	UDP-glucose ceramide glucosyltransferase-like 1	3	3	15.5	7.4	30.0	32.4	30.1
IPI:IPI00115546.4	Gnao1	guanine nucleotide binding protein, alpha O	2	2	15.3	10.1	32.6	27.3	29.9
IPI:IPI00120245.1	Itgav	integrin alpha V	2	2	14.5	9.0	37.4	23.5	30.1
IPI:IPI00311682.5	Atp1a1	sodium/potassium transporting ATPase alpha 1 polypeptide	3	3	14.2	6.5	43.3	19.0	31.2
IPI:IPI00124221.1	Atp1b3	sodium/potassium transporting ATPase beta 3 polypeptide	3	4	13.7	5.1	33.9	28.3	32.7
IPI:IPI00652902.1	Gnai2	guanine nucleotide binding protein, alpha inhibiting 2	2	2	13.5	5.1	35.8	24.3	34.8
IPI:IPI00271951.5	Pdia4	protein disulfide isomerase associated 4	4	4	13.3	10.1	25.1	27.0	37.8
IPI:IPI00123996.1	Nrp1	neuropilin 1	2	3	12.8	7.0	49.3	19.6	24.1
IPI:IPI00752565.1	Igsf8	immunoglobulin superfamily, member 8	3	3	12.2	4.8	49.0	21.2	25.0
IPI:IPI00118569.1	Gna13	guanine nucleotide binding protein, alpha 13	3	3	10.1	14.6	35.1	32.8	17.6
IPI:IPI00845689.1	Tmed9	transmembrane emp24 transport domain containing 9 (gp25L2)	5	7	9.7	7.2	44.2	26.1	22.5
IPI:IPI00469000.4	Slc39a6	metal ion transporter ZIP6	3	3	7.3	3.2	29.9	32.8	34.2
IPI:IPI00458003.1	Enpp3	ectonucleotide pyrophosphatase/phosphodiesterase 3	2	2	5.4	5.9	43.4	25.1	25.5
IPI:IPI00273801.3	Slc39a10	metal ion transporter ZIP10	2	2	4.3	2.4	36.3	16.7	44.6
IPI:IPI00831568.1	L1cam	L1 cell adhesion molecule	3	3	3.5	10.9	30.9	26.7	31.5

a Candidate interactors are listed in order, with the position of a given protein in the table reflecting the percentage of primary structure corresponding to the combined unique CID spectra. In instances where a subset of CID spectra were matched to more than one isoform or member of a protein family, only the highest scoring entry was selected unless an independent identification was supported by at least two unique CID spectra. Proteins were sorted into specific versus unspecific binder categories based on their iTRAQ distribution, i.e. proteins were considered unspecific interactors if their derived CID spectra revealed iTRAQ114 signature mass peak signal intensities which exceeded 15% of combined and normalized (100%) intensities for iTRAQ114-117 mass peaks. Only specific interactors are shown in this table. The complete dataset can be viewed in Table S1.

b Only CID spectra underlying different peptides were considered, i.e. if the same peptide was identified with different charge states or modifications it counted as one hit.

c Total number of unique CID spectra. Please note that the same peptide was only counted more than once if it was identified with different charge states or modifications.

d Percent sequence coverage based on the presence of peptides for which no higher ranked assignment to other proteins could be made.

e For the calculation of iTRAQ values the intensity of individual peptide associated iTRAQ signature peaks was normalized to combine to 100% per peptide and subsequently averaged. Standard deviations were determined and are listed in Table S1.

Bait proteins are shown in bold font.

**Figure 3:**
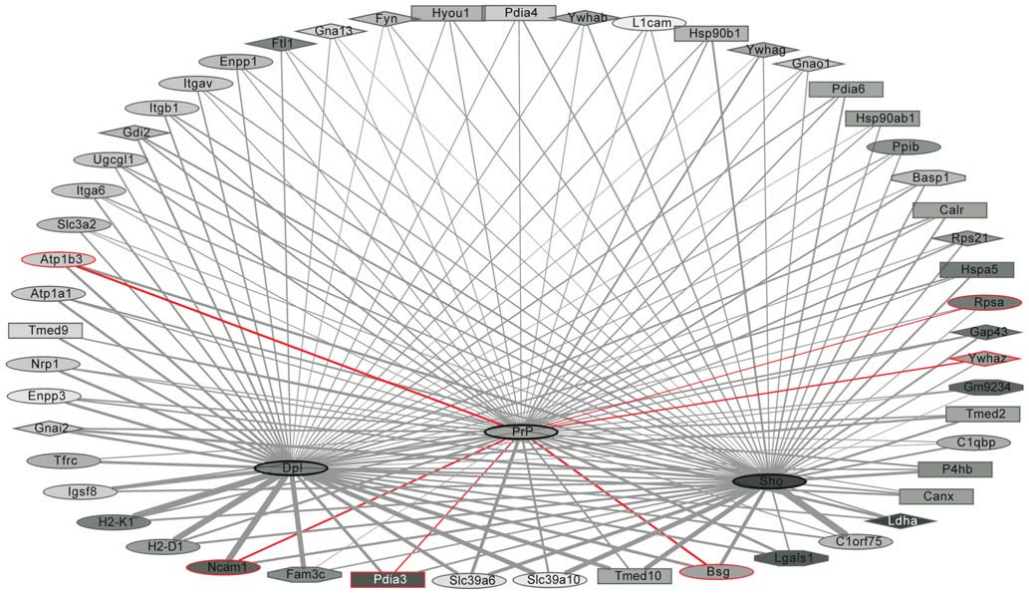
The mammalian prion protein family interactome. Network depicting proteins identified in spatial proximity to Dpl, PrP^C^ and Sho as nodes and their direct or indirect interactions as spokes. Components identified in previous studies by other investigators are shown with red lines surrounding and red edges connecting the nodes. The intensity of gray shading of nodes correlates directly with the fractional sequence coverage with which individual components were identified. Increased spoke thickness indicates stronger co-enrichment with the connected bait protein. Node shapes indicate predominant cellular localization of proteins, i.e. ER and Golgi compartments (rectangular), plasma membrane (oval), cytosol (diamond) or secreted (octagonal). Nodes depicting proteins whose presence in the samples correlated strongest with the presence of a given bait protein are arranged in spatial proximity to the respective bait protein nodes in the bottom half of the network.

We next explored the known cellular localization of candidate interactors based on bioinformatic methods and literature mining. This investigation revealed that multiple proteins in the dataset were likely to encounter the three bait proteins during their early passage through the secretory pathway, since they constitute (i) classical ER chaperones (heat shock protein 5, Hspa5; calnexin, Canx; calreticulin, Calr; endoplasmin, Hsp90b1), (ii) isomerases which facilitate disulfide or proline cis-trans rearrangements (protein disulfide isomerase associated 3, Pdia3; protein disulfide isomerase associated 4, Pdia4; prolyl 4-hydroxylase beta polypeptide, P4hb; peptidylprolyl isomerase B, Ppib), or (iii) proteins involved in the trafficking between ER and Golgi compartments (transmembrane emp24 transport domain containing 9, Tmed9; and transmembrane emp24-like trafficking protein 10, Tmed10). Other proteins in the dataset were likely to reside in spatial proximity to the mature bait proteins at the plasma membrane, because they are themselves known to be either (iv) embedded in the plasma membrane through transmembrane (TM) domains (NCAM; transferrin receptor, Tfrc; integrins; neuropilin 1, Nrp1; L1 cell adhesion molecule, L1cam; basigin, Bsg), or constitute (v) secreted proteins (galectin-1, Lgals1; family with sequence similarity 3, member C, Fam3c; ectonucleotide pyrophosphatase/phosphodiesterase 1 and 3, Enpp1/3) or (vi) cytosolic proteins (14-3-3 proteins zeta/beta/gamma, Ywhaz/Ywhab/Ywhag; guanine nucleotide binding proteins, Gnao1/Gnai2/Gna13; and Fyn proto-oncogene) that attach to the plasma membrane from outside or within the cell, respectively (Fig. 3). In contrast to the above proteins that were designated as specific interactors by their iTRAQ reporter ion ratios, proteins which were equally represented in control and specific interactome cells and therefore constituted unspecific binders could be mapped to a wide range of cellular compartments including the nucleus and mitochondria. This result was expected based on the known cellular biology of PrP^C^ and therefore served as strong biological validation of the iTRAQ-based discrimination of specific interactors from unspecific contaminants.

### Interactions amongst members of the mammalian prion protein family

It was anticipated that iTRAQ reporter ion signals in CID spectra derived from tryptic peptides of the three bait proteins would reflect the logic of the iTRAQ conjugation step. For example, CID spectra which supported the identification of Dpl had to be mainly contributed by the sample which was labeled with the iTRAQ 115 reagent. A close inspection of the low mass range of CID spectra confirmed that this expectation was met for all three bait proteins and thereby served as an internal control for the reliability with which relative iTRAQ reporter ion intensities could be employed to trace the contribution of samples to the identification of proteins (Table 1). Interestingly, however, a closer inspection of the above spectra also revealed that approximately one-third of the peptides which supported the identification of PrP were contributed by the FLAG-Dpl and FLAG-Sho affinity purified samples. This observation was supported by five CID spectra consistent with the interpretation that the purification of FLAG-Dpl and FLAG-Sho caused a significant co-purification of endogenous wild-type (wt) PrP^C^ (*P*<0.05). A similar co-purification of endogenous wt Sho in N2a cells was observed with FLAG-Dpl and FLAG-PrP. A co-purification of wt Dpl with FLAG-PrP and FLAG-Sho baits was neither observed nor expected because no endogenous expression of Dpl can be reliably detected in N2a cells (or indeed in the adult mouse central nervous system) [23]. Taken together, these data suggest that PrP, Dpl and Sho are capable of residing in close spatial proximity at the cell membrane and may engage in reciprocal contacts with each other.

### Direct versus indirect PrP interactors

It is unlikely that members of the mammalian prion protein family engage in direct interactions with all candidate interactors we identified. For example, some retrieved proteins may represent complexes of the type PrP^C^- crosslink - interactor 1 - crosslink - interactor 2. Such indirect interactors are often less strongly represented in such datasets, due to both the non-stoichiometric crosslinking that can be achieved and the stringent washing conditions applied. A good correlate for the relative abundance of a protein in proteomics investigations of this nature is the sequence coverage with which it was identified. Based on this rationale, the three bait proteins may bind directly to lactate dehydrogenase (Ldha), protein disulfide isomerases Pdia3 and P4hb, galectin-1, NCAM, Gm9234 (also known as EG668548), neuromodulin (Gap43), LRP and its ribosomal protein partner Rps21, heat shock proteins Hspa5 and Hsp90ab1, histocompatibility antigens H2-K1 and H2-D1, Fam3c, Ppib, calnexin, calreticulin, 14-3-3 zeta/delta and basigin, as these proteins were identified with the highest sequence coverage (25–75%).

Due to their relatively small size, members of the mammalian prion protein family would be expected to contain a small number of distinct surface domains that can engage in direct protein-protein interactions. Consequently, at least a subset of binders may bind to the same face within the bait proteins and may even resemble each other in their primary structure or structural folds. When candidate interactors were subjected to scrutiny from this angle, similarities between NCAM and basigin, two of the proteins represented with the highest sequence coverage in the dataset, became apparent. Not only do both proteins harbor Ig-like domains, but a subset of these domains bear a striking resemblance in their sequence, an observation made previously [24],[25]. Interestingly, these very same Ig-like domains are capable of binding to membrane proteins carrying oligomannosidic N-glycans [26],[27],[28],[29]. The latter modification is only found on a relatively small number of mature N-glycosylated proteins expressed mainly in the brain, because oligomannose structures typically represent transient intermediates subject to additional posttranslational modification during their passage through the secretory pathway [26],[29],[30]. Amongst the few known proteins carrying oligomannosidic N-glycan structures are the cell adhesion molecule L1, the β-subunit of the Na/K ATPase, the transferrin receptor, integrins [31] and members of the ectonucleotide pyrophosphatase/phosphodiesterase protein family [30]. Remarkably, not only were all the above proteins present in our dataset, but their iTRAQ ratios also documented their specific co-purification with members of the mammalian prion protein family. In light of the relatively low sequence coverage observed for proteins carrying oligomannosidic N-glycans, their presence in the dataset likely reflects indirect binding to the bait proteins mediated by the mentioned molecules that harbor Ig-like domains with an affinity for oligomannosidic N-glycans.

### PrP forms high-molecular weight complexes with proteins carrying oligomannosidic N-glycans

**Figure 4:**
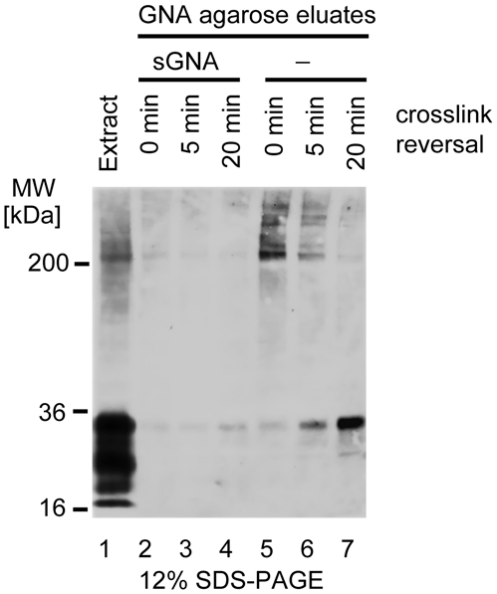
Evidence for PrP^C^ in high molecular weight protein complexes captured by a lectin with specificity for oligomannosidic glycans. Brain extracts from mice subjected to transcardiac perfusion crosslinking were passed over snowdrop lectin agarose. Western blot analysis of eluate fractions with 7A12 antibody documented presence of PrP^C^ in high molecular weight complexes (lane 5). Only weak bands were detected when the affinity capture step was preceded by incubation of extracts with soluble snowdrop lectin (sGNA, lane 2). Crosslink reversal by 5-min (lanes 3 and 6) or 20-min (lanes 4 and 7) heat treatment of samples caused a shift of PrP^C^ reactive bands to levels of fully glycosylated PrP^C^ consistent with the release of PrP^C^ from crosslinked complexes.

To investigate whether PrP^C^ also resides in spatial proximity to membrane proteins equipped with oligomannosidic N-glycans in the mouse brain, we subjected anaesthetized wild-type mice to brief crosslinking with formaldehyde to stabilize protein-protein interactions *in vivo* prior to the disruption of cellular integrity [16]. Perfused mouse brains were then rapidly dissected and homogenized in the presence of a quenching reagent. To verify the success of the crosslinking reaction and characterize the size distribution of crosslinked protein complexes containing PrP^C^ an aliquot of extracts was passed through a pre-calibrated size-exclusion chromatography column. This pilot analysis documented that PrP^C^ was found in protein complexes co-eluting with calibration markers which span a range of 160 kDa to 669 kDa. Uncrosslinked PrP^C^ could be recovered from the crosslinked complexes by a crosslink reversal procedure based on the administration of heat and reducing agents (Fig. S1). Subsequently, the crosslinked extracts were passed over snowdrop lectin (*Galanthus nivalis* agglutinin, GNA) agarose, a lectin which specifically recognizes oligomannosidic N-glycans [30]. Eluate fractions, when subjected to Western blotting analysis, documented the presence of a subset of PrP^C^ in high-molecular weight protein complexes that were specifically enriched by snowdrop lectin agarose (Fig. 4). The binding to the affinity matrix was determined to be specific by (i) the PrP^C^-specific band pattern in the eluate fraction demonstrating the selective capture of PrP^C^ in high-molecular weight crosslinked complexes, rather than in its uncrosslinked form; and (ii) the observation that pre-incubation of the extract with soluble snowdrop lectin prevented affinity capture. A crosslink reversal time series revealed that the high-molecular weight complexes preferentially contain fully-glycosylated PrP^C^ consistent with the notion that this interaction involves mature PrP^C^ present at the cell surface.

### A subset of cellular P4hb, Pdia3 and calreticulin reside at the cell surface of neuroblastoma cells

**Figure 5:**
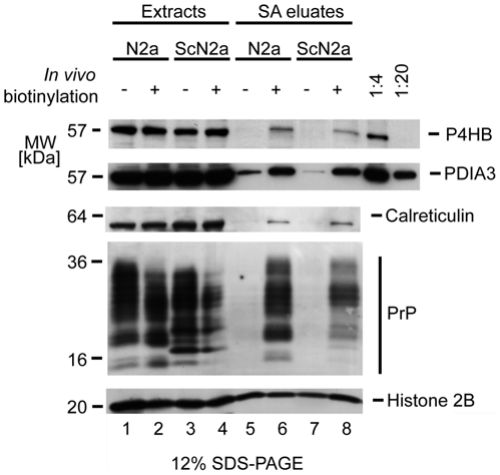
Evidence for cell surface localization of a subset of PDIs and calreticulin in mouse neuroblastoma cells. N2a and ScN2a cells were subjected to cell surface biotinylation or mock treatment. Subsequently, cellular extracts were side-by-side affinity purified on a streptavidin agarose matrix. Extracts and streptavidin agarose eluate fractions were analyzed by Western blotting and membranes probed with antibodies directed against histone 2B, PrP, P4hb, Pdia3 and calreticulin. The relative strength of P4hb-, Pdia3- and calreticulin-specific signals present in eluate fractions from biotinylated (lanes 6 and 8) versus non-biotinylated samples (lanes 5 and 7) is consistent with the conclusion that a subset of these proteins resided at the cell surface during the biotinylation step. The relative intensity of P4hb- and Pdia3-derived signals in extract (40 µg total protein loaded per lane) and eluate fractions can be estimated from the concomitant analysis of 1/4 and 1/20 of the extract fraction shown in lane 1. Thus, P4hb and Pdia3 signals in the eluate fraction (captured from 2 mg total extract) were equivalent to or exceeded the amount of these proteins present in 2 µg of extract. Please note the presence of histone 2B in all eluate fractions (lanes 5–8), in support of the conclusion that the protein binds unspecifically to the affinity matrix.

A conspicuous feature of the Dpl/PrP/Sho-interactome dataset constituted the identification of multiple PDIs. In particular, Pdia3 (also known as ERp57) was represented with strong sequence coverage (61%) and its identification was further corroborated by similarly strong identifications for calnexin and calreticulin, known interactors of this PDI. Whereas binding of PDIs to both Dpl and PrP was expected due to the presence of internal disulfide bridges in these proteins, co-segregation with Sho (Pdia3 117∶114 iTRAQ reporter ion ratio of 2∶1), which does not carry a disulfide bridge, was an unexpected finding. PDIs are known to harbor both isomerase and chaperone functions. In light of previous calls for an involvement of chaperone-like activities in the conversion of PrP^C^ to PrP^Sc^, we asked whether a subset of PDIs may play a role in this process. It is currently thought that the conversion of PrP^C^ occurs only after it has reached the cell surface [32],[33], i.e. well-removed from its presence within the ER where it would be expected to encounter PDIs. Thus, if PDIs were involved in PrP^C^ conversion, these proteins would also have to be present outside the ER, preferably at the plasma membrane. Intriguingly, a small number of reports suggest that, at least in some cells, this might indeed be the case (please refer to Discussion section). To investigate whether Pdia3, P4hb and calreticulin are also present at the cell surface in mouse neuroblastoma cells, a cell surface biotinylation experiment was undertaken. Cells were incubated for a brief duration with a cell-impermeable biotin-labeling reagent, which conjugates to primary amines through an N-hydroxysuccinimide ester group, or were subjected to mock treatment. To determine the degree to which broken cells were present at the time of cell surface biotinylation, the downstream analysis was extended to histone 2B. If a large amount of broken cells had been available, histone 2B would have been present at appreciable levels in the cell culture medium resulting in an unequal signal, absent in the mock-treated sample and strong in the biotinylated sample. The observation that histone 2B signals in streptavidin agarose eluates were equal documented that low levels of non-biotinylated histone 2B had been captured unspecifically and served as evidence that levels of extract employed in the side-by-side streptavidin agarose capture steps had indeed been equal. Western blot analysis of extract and streptavidin eluate fractions probed with a PrP^C^-specific antibody served as a positive control (Fig. 5). Taken together this experiment revealed that a subset of Pdia3, P4hb and calreticulin was enriched in streptavidin eluate fractions derived from *in vivo* biotinylated cells compared to the corresponding samples derived from mock-treated control cells. The possibility that this distribution may have been the result of an unspecific binding to the affinity matrix was excluded based on a comparison with histone 2B signals observed evenly across all streptavidin eluate fractions, a distribution expected for a protein which binds to the affinity matrix unspecifically. A calculation based on a semi-quantitative titration analysis of Western blot signal intensities revealed that the relative amounts of PDIs and calreticulin at the plasma membrane are small compared to their respective quantities in the ER, but constitute at least 1∶1000 of the total amounts in these cells. Actual amounts may exceed these estimates as the calculation did not account for the non-stoichiometric labeling achieved during the biotin conjugation step and losses of biotin-derivatized PDIs incurred during the streptavidin affinity purification step.

### Inhibitors of protein disulfide isomerases increase PrP^Sc^ levels in a subset of ScN2a cell clones

**Figure 6:**
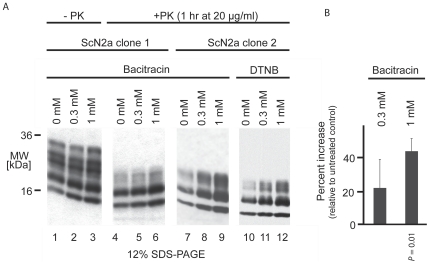
Inhibition of protein disulfide isomerases causes accumulation of PrP^Sc^ in a subset of ScN2a cell clones. A, Bacitracin or DTNB, reagents known to inhibit PDIs through orthogonal modes of binding, were added at the indicated concentrations to the cell culture medium and left on the ScN2a cells for a duration of 2 days. Following cell lysis, protein levels were adjusted and a subset of samples subjected to digestion with proteinase K (lanes 411). All samples were analyzed by Western blotting with a PrP specific antibody. Please note the increase in intensity of low molecular weight bands characteristic for the presence of PrP^Sc^ even without proteinase K treatment (lanes 1–3). B, A two-sample, two-tailed Student's *t*-test assuming unequal variance was utilized to determine the statistical significance of bacitracin-treated band intensities compared to normalized untreated samples. The result of this analysis indicated a significant increase of PrP^Sc^ in ScN2a clones 1 and 2 treated with 1 mM bacitracin (*P*-value = 0.01).

We next addressed whether PDI-inherent chaperone activities influence PrP conversion in prion-infected mouse neuroblastoma cells (ScN2a). The identification of four different PDIs in the dataset suggested that the knockdown of just one PDI may be uninformative. We therefore employed inhibitors known to interfere broadly with the activity of PDIs. One possible outcome of PDI inhibition *in vivo* would include a depression of PrP^Sc^ levels by inhibiting PDI-mediated unfolding of an obligate precursor form of PrP^C^. Interestingly, both the addition of the dodecapeptide antibiotic bacitracin or 5,5′-dithiobis(2-nitrobenzoic acid) (DTNB), membrane-impermeable reagents known to inhibit PDIs through noncovalent and covalent modes of binding, respectively [34], caused an increase in the level of proteinase K-resistant PrP^Sc^ (Fig. 6). The effect sizes correlated with inhibitor concentrations applied, and in the presence of up to 1 mM of either of the inhibitors, the viability of ScN2a cells (assessed by the trypan blue dye exclusion assay) was not impaired during the course of the experiment. A direct inhibitory effect of these reagents on the proteinase K digestion itself can be ruled out, since levels of the faster migrating PrP^Sc^-derived signals, characteristic for the presence of PrP^Sc^ in this disease model (attributed to the action of endogenous proteases that produce a “trimming” of PrP N-terminal sequences [35],[36]), were similarly elevated even prior to the proteinase K digestion step. This observation was made with two clones of ScN2a cells obtained from independent sources; surprisingly, however, it could not be observed with an isolate of chronically prion-infected GT1 cells or two additional ScN2a clones derived from yet other sources (Fig. S2), possibly indicating the existence of cell line- or subclone-specific PDI family member expression profiles. We note that variations in the behavior of different ScN2a cell clones are well-documented, as is the occurrence of heteroploidy. While variations in our experimental paradigm preclude a generalized observation, they nonetheless put a spotlight on ER-derived chaperones that may engage in physiological interactions with PrP^C^ even at the cell surface, as discussed below.

## Discussion

The work presented here may comprise the most comprehensive cell-based analysis of the PrP^C^ interactome to date. Furthermore, it is, to our knowledge, the first study to extend the PrP^C^ interactome characterization to other members of the mammalian prion protein family. Critical technical advances from previous PrP^C^ interactome work constitute the incorporation of FLAG affinity purification tags and the use of iTRAQ-based quantitative mass spectrometry for the discrimination of specific from unspecific interactors. These conceptual choices greatly facilitated bioinformatic analyses and afforded an in-depth glimpse into the molecular environment of these proteins.

The data presented support the conclusion that all three members of the mammalian prion protein family populate highly similar molecular environments when expressed in neuroblastoma cells. This interpretation is based on (i) the high degree of overlap amongst candidate interactors identified for each of the three bait proteins, and (ii) the reciprocal co-capture of endogenous prion proteins in affinity purifications of FLAG-tagged bait proteins. Due to the stringent washing steps applied following the FLAG tag-dependent capture step, this observation is unlikely to represent an unspecific co-enrichment of raft-like membrane domains rich in GPI-anchored proteins. Instead, it points at a close spatial proximity, if not direct interaction, of members of the prion protein family at the plasma membrane. The possibility of a direct interaction between Dpl and PrP^C^ had been raised previously in a report demonstrating rescue of a Dpl-dependent ataxic phenotype in two PrP-deficient (*Prnp* ^0/0^) mouse lines by reintroduction of PrP^C^ [37]. Recently, a similar rescue of Dpl-mediated toxicity was documented upon overexpression of PrP^C^ or Sho in a primary cell culture model of cerebellar granule neurons which exhibits undetectable endogenous levels of these proteins [20]. However, it has remained unresolved whether these observations depend on the spatial proximity of the respective proteins as opposed to action upon other proteins in shared or intersecting biochemical pathways.

The comparison of protein-protein interaction data reported here with previous data based on either the immunocapture of endogenous PrP^C^ [16] or myc-tagged transgenic PrP^C^ [38] harvested from mouse brains suggests that the move to a cell model may, together with the increase in scale, contributed to a reduction in protein ‘noise’ and thereby an enhanced analysis depth. As a result, the study revealed spatial proximity of the bait proteins to multiple proteins (NCAM, LRP, Na/K ATPase and PDIs) which had previously been proposed to bind to PrP^C^ in the context of widely-differing experimental paradigms.

A comparison of input and eluent protein amounts in this study suggests that less than 0.1% of membrane-associated proteins present in the crosslinked cellular extracts were withdrawn by individual immunoaffinity capture steps. The strength with which a given protein was identified in proximity to our bait proteins may at least partially reflect its relative abundance in the vicinity of the bait protein. Multiple lines of evidence in our dataset support the notion that candidate proteins we identified do indeed reside naturally in spatial proximity to members of the mammalian prion protein *in vivo*: (1) the proteins we identified originate from cellular compartments to which PrP^C^ is expected to localize; (2) some highly abundant cellular proteins (tubulin, actin, histones) were only seen in the list of unspecific binders to the affinity matrix; (3) no nuclear proteins were found despite the use of deoxycholate in the cell lysis buffer, a detergent known to solubilize nuclear membranes; and (4) ten out of about fifty candidate interactors in the dataset had previously been reported to interact with the cellular prion protein. The majority of candidate interactors identified in this work (e.g. PDIs, calreticulin, LRP, 14-3-3 proteins and Na/K ATPase) are known to be widely expressed across a large number of diverse tissues and as such are plausible interactors of the bait proteins also outside of N2a cells. Other interactors exhibit a more restricted tissue expression and are primarily known for their expression in cell types belonging to the neuronal lineage (e.g. NCAM and L1), in agreement with the predominant and partially overlapping expression of PrP^C^ and Sho in a subset of these cell types. For Dpl, a protein normally confined to the testis, a physiological proximity to some of these proteins is unlikely, except in the artificial experimental circumstances (described above) which result in ectopic expression of Dpl in the brain.

Proteins that are expected to engage in short-lived interactions are typically underrepresented in interactome datasets of this kind, and as such it was not surprising to find that enzymes known to modify N-glycans or responsible for attachment of the GPI anchor to PrP^C^ were not identified. Moreover, it is likely that the crosslinking chemistry we employed may have precluded the detection of some interactions which lack suitably-positioned functional groups required for the generation of productive covalent bonds. A bias towards covalent interactions that do not sterically block the FLAG epitope may have further skewed the dataset. The above note of caution should also be extended to instances in which candidate interactors co-purify only with a subset of the bait proteins, as seen here for the largely uncharacterized proteins Fam3C and C1orf75 that co-purified with Dpl and Sho, respectively. More work will be needed to determine whether this apparent selective binding to a subset of bait proteins represents a technical artifact or reflects underlying differences in the biology of the bait proteins.

The conversion of the cellular prion protein has repeatedly been proposed to involve additional host-encoded factors. In particular, the participation of chaperones in this process has remained an attractive hypothesis based on theoretical considerations and species barrier effects [39]. Any chaperone to fulfill this role would be expected to bind to PrP^C^ and reside at the cellular plasma membrane. The latter conclusion can be drawn from multiple lines of investigation in cell models of prion disease [32],[33]. This study revealed multiple chaperone molecules such as Pdia3, P4hb and calreticulin that may share these characteristics. The subsequent observation that pharmacological inhibition of PDIs causes an increase in proteinase K-resistant PrP^Sc^ levels in a subset of ScN2a cell clones is exciting as it suggests PDIs may, under certain circumstances, be protective in the context of prion disease, thereby extending observations from an earlier report which documented an increase in Pdia3 levels in prion infected mice and an inverse correlation of PrP^Sc^ toxicity and Pdia3 expression levels in mouse neuroblastoma cells [40]. Consistent with a more direct role in PrP conversion reactions, PDIs have been shown to localize to lipid rafts in other cell systems [41], and experiments which employed an *in vitro* PrP^Sc^ amplification methodology suggested that free sulfhydryl groups — through an unidentified mechanism — play a critical role in this reaction [42]. The cellular disulfide exchange homeostasis is a complex aspect of cell biology due to the existence of more than ten PDI family members in mammals with distinct but only partially understood substrate specificities, cellular localizations and intricate regulations [43]. The scope and emphasis of this study was such that it could only begin to address the role these molecules may play in prion biology. However, it is attractive to hypothesize that some cells may put a low ER leakage rate of PDIs to good use by employing these molecules to “patrol” their plasma membrane and prevent undesired occurrences of protein misfolding. In disease, or when pharmacologically suppressed, the rate of misfolding may exceed the rate of PDI-mediated rescue and thus produce a net accumulation of PrP^Sc^. Similar concepts implicating rates of synthesis and degradation have been proposed in a ‘dynamic susceptibility’ model based on observations in N2a cells [44].

Similar to PDIs, calreticulin — in addition to its well established role in the ER — may play an important function in cell-cell recognition in apoptosis and cell-surface recognition during cellular attachment to laminin [45]. This is relevant in the current context as multiple lines of evidence suggest that PrP^C^ confers protection against apoptosis and plays a role in adhesion to the extracellular matrix [46]. While it remains to be determined whether the spatial proximity of PrP^C^ and calreticulin plays a role in these cellular activities, we noted with interest that calreticulin binding to laminin depends on the presence of oligomannosidic N-glycans on laminin [47],[48]. The link to oligomannose-carrying N-glycans was intriguing because the same posttranslational structures had also been signaled by a comprehensive bioinformatic analysis to which our dataset was subjected. The latter pointed to a previously reported similarity in the ability of NCAM and basigin, two proteins we identified with high sequence coverage, to bind to oligomannose-bearing membrane proteins through a specialized Ig-like domain. Surprisingly, scrutiny of the dataset for proteins reported to carry this posttranslational modification subsequently established their strong enrichment. A snowdrop lectin binding experiment then confirmed that PrP^C^ indeed resides in spatial proximity to proteins carrying oligomannosidic N-glycans in the intact mouse brain. From these observations, a model emerged based on which members of the mammalian prion protein family may primarily ‘organize’ their molecular microenvironment by binding to NCAM and basigin which in turn recognize a small subset of proteins carrying N-glycans of the rare oligomannose type. The latter proteins may themselves be part of protein assemblies containing more than one component.

One of these assemblies surrounds the oligomannosidic carbohydrate-carrying β-subunit of the Na/K ATPase (Atp1b3), a protein complex known to also consist of an α-subunit and a tightly associated G-protein complex, both of which were observed in this dataset and an earlier PrP^C^ interactome investigation based on *in vivo* crosslinked proteins in the mouse brain [16]. Recently, a role for the cellular prion protein in lactate metabolism has been proposed [49]. The authors also documented interactions of PrP^C^ within a molecular assembly involving basigin and sodium/potassium channels. Our data corroborate key interactions proposed in this initial study and documented that this novel concept is not restricted to PrP^C^ but also extends to Dpl and Sho.

A molecule which may participate in multiple sub-nodes is the integrin β1 subunit (Itgb1). In addition to Itgb1, our data documented spatial proximity of all three bait proteins to the laminin-specific integrin α6 (Itga6) and the integrin αv (Itgav). The binding of bait proteins to these integrins is unlikely to have occurred independently of Itgb1 binding, because stable Itgb1–Itgav (integrin αvβ1) or Itgb1–Itga6 (integrin α6β1, also known as VLA-6) heterodimeric complexes are well described in the literature [50]. VLA-6 plays an important role in the adhesion of cells to laminin and in this capacity has been shown to associate with ecto-calreticulin [51] or LRP [52]. The identification of LRP (Rpsa), better known as the 37-kDa/67-kDa laminin receptor in the prion research field and currently under investigation as a therapeutic target for prion disease [53],[54],[55], corroborated previous experiments documenting its spatial proximity to PrP^C^ (and possibly Dpl [56],[57]) and was complemented by the identification of ribosomal protein S21 (Rps21), a protein which not only can bind to LRP but shares with the latter the ability to exert a ribosome-independent function at the plasma membrane [58],[59]. Finally, Itgb1-containing integrins may functionally interact with CD98 (Slc3a2), a type II TM protein [60],[61]. The circle to basigin can be closed by a well-established interaction between CD98 and basigin [62],[63]. Although the details of this interaction are far from understood, it has been suggested that binding of CD98 to basigin may play a role in cell fusion control, including the specialized fusion of a sperm to the membrane of an egg that precedes fertilization [64],[65],[66]. Given the independently proposed role of Dpl in sperm fusion events [67], it will be of interest to determine whether the direct interaction of Dpl with the above protein machinery bears functional significance in this context.

During the assembly of this work, a systems biology study reported on global changes in protein expression during the course of prion disease in mice [68]. Not surprisingly, a comparison of the two datasets revealed little overlap in the proteins which we found to reside in proximity to PrP^C^ and the proteins whose (mRNA) expression is affected in disease. This finding is consistent with an interpretation that global responses to prion disease may not center on proteins which are spatially close to PrP^C^ and also the distinct technological bases of the two datasets. A notable exception to this observation may represent proteins from the ZIP metal ion transporter family of which two paralogs were found in the vicinity of PrP^C^ in this study (ZIP6 and ZIP10), and another paralog ZIP14 was found to be upregulated during the course of prion disease in five independent mouse strains. Interestingly, these three ZIP proteins belong to a common branch (referred to as the LIV-1 subfamily) within mammalian ZIP paralogs whose N-terminal extracellular domain harbors features that are strikingly reminiscent to sequence features found within members of the mammalian prion protein family itself. Extensive follow-up investigations have led to the surprising conclusion that the mammalian prion gene family is phylogenetically derived from a ZIP ancestor molecule (Schmitt-Ulms *et al.*, in revision).

The possible participation of PrP^C^ family members in both lactate metabolism and laminin adhesion are unlikely to represent independent functional specializations exerted in the setting of distinct protein microenvironments. Arguing against such a scenario are (i) the known interactions between molecules we identified in close proximity to members of the mammalian prion proteins, and (ii) the recurring theme of oligomannosidic N-glycans which emerged from the bioinformatic analysis. Instead, a unifying model came into focus which suggests that the involvement of PrP^C^ in these biological functions is mediated by remarkably interconnected molecular machineries. In proposing the model, it was not our intent to oversimplify a complex molecular biology that surely must underlie interactions in this network. It is, for example, likely that connections amongst proteins in this network are not arranged in a chain-like and static fashion through a series of single contact sites. Rather, a more realistic representation of such a network would predict a system built upon highly cooperative and dynamic interactions.

### Conclusion

Taken together, this work suggests that members of the prion protein family may be embedded in specialized membrane domains characterized by an extended molecular network which supports functions in adhesion control, lactate metabolism and cell fusion events. Our investigation leads to the conclusion that biochemical interactions among the members of the mammalian prion protein family exist. It confirms multiple candidate interactors of PrP^C^ in a unified paradigm including an independent verification of the laminin receptor precursor (LRP) as an authentic PrP interactor. It thereby not only reconciles a fragmented literature, but should facilitate future efforts to further define the relative contribution of these interactors to the biology of PrP^C^. Novel interactors uncovered in this study paint a coherent picture based on which an argument for the spatial proximity of NCAM, basigin, Na/K ATPase and LRP can be made, even independent of their ability to bind to PrP^C^. Moreover, data presented here raise many new questions as to (i) whether biological outputs of PrP^C^ implied by the “guilt-by-association” logic will be confirmed, (ii) the importance of oligomannosidic N-glycans for the structural integrity and assembly of the molecular environment of PrP^C^, (iii) the role which a subset of cellular chaperones may play in PrP biology and disease, and (iv) the exact environment which hosts the conversion of PrP^C^. It is hoped that this work will stimulate investigations which will not only resolve these questions, but lead to insights from which a therapeutic strategy for addressing prion diseases can be derived.

## Materials and Methods

### Ethics statement

All procedures which required the handling of mice were conducted in accordance with an animal use protocol (No. 20006633) approved by the University of Toronto Animal Care Committee.

### Antibodies

The mouse monoclonal antibody directed against PrP (clone 7A12) was a generous gift from Dr. Man-Sun Sy (Case Western Reserve University School of Medicine, Cleveland, OH, USA). The anti-FLAG M2 antibody (F3165) was obtained from Sigma-Aldrich (Oakville, ON, Canada). Anti-ERp57 (Pdia3) antiserum was raised against glutathione *S*-transferase-fused mouse ERp57. Anti-P4hb antiserum (SPA-891) was purchased from StressGen Biotechnologies (Victoria, BC, Canada).

### Clones

FLAG affinity tags (N-DYKDDDDK-C) were inserted before residue 29 of mouse PrP, residue 27 of mouse Dpl, and residue 26 of mouse Sho (all in the pcDNA3 mammalian expression vector) using standard PCR-based mutagenesis techniques. The identity of all constructs was verified by DNA sequencing.

### Cell culture, *in vivo* crosslinking, inhibitor treatments and cell viability assay

Cell culture and formaldehyde crosslinking of N2a cells followed a protocol described before [10]. For bulk selection of stably transfected cells, cultures were expanded in the presence of 1 mg/mL G418 and maintained at a concentration of 0.2 mg/mL G418. RML-infected N2a cells (ScN2a) were maintained as above. For PDI inhibition experiments, DTNB or bacitracin (Sigma-Aldrich) was added to the cell culture medium at indicated concentrations followed by a two-day incubation at 37°C. The effect of inhibitor treatments on cell viability was assessed by the Trypan blue dye exclusion assay. Briefly, 0.4% Trypan blue was gently mixed with trypsinized cells and incubated for 5 minutes at room temperature. Subsequently, the percentage of viable cells per volume equivalent was determined with the use of a hemocytometer.

### Affinity purification of bait proteins

Approximately 10^9^ *in vivo* FA-crosslinked cells each of control and FLAG-prion expressing N2a cell lines were lysed in homogenization buffer (50 mM NH_4_Cl, 80 mM Tris, pH 8.0) supplemented with 1× Complete Protease Inhibitor Cocktail (Roche, Palo Alto, CA, USA). To ensure near quantitative extraction of membrane proteins, an equal volume of extraction buffer (20 mM NaCl, 1% sodium deoxycholate, 1% NP-40, 20 mM Tris, pH 8.0) was added, followed by a 30-min incubation and 5-min sonication in a water bath sonicator. Insoluble cellular debris was removed by high-speed centrifugation (100,000×*g*, 1 h). Subsequently, the crosslinked bait protein complexes were immunoaffinity-captured on anti-FLAG-agarose (Sigma-Aldrich). During this step samples were gently agitated on a turning wheel for 12 h, then washed extensively with 0.5 M NaCl, 0.05% SDS, 1% NP-40, 20 mM HEPES, pH 7.3, and detergents removed by a pre-elution wash with 10 mM NH_4_HCO_3_, pH 8.0. Proteins were eluted by acidification with 0.2% trifluoroacetic acid, 20% acetonitrile, pH 2.0.

### Protein reduction, alkylation and trypsinization

Protein-containing fractions were denatured in the presence of 6 M urea, 20 mM NH_4_HCO_3_, pH 8.0, followed by reduction with 1 mM tris-(2-carboxyethyl)-phosphine for 30 min at 60°C and alkylation with 2.5 mM 4-vinylpyridine for 1 h at room temperature in the dark. Samples were diluted four-fold to ensure that the concentration of urea did not exceed 1.5 M. Tryptic digestion was initiated by the addition of 1% (wt/wt) of side chain-modified, TPCK-treated porcine trypsin and allowed to proceed at 37°C for 6 h.

### iTRAQ labeling

Following trypsinization, equal quantities of tryptic peptide mixtures were spiked with 1 pmol of synthetic (Glu1)-Fibrinopeptide B (GluFib) (Sigma-Aldrich) to serve in the downstream analysis as an internal control for the efficiency of individual labeling reactions. Equal labeling with all four reagents was confirmed by equal intensities of 114∶115∶116∶117 signature peaks upon forced fragmentation of the GluFib [M+2H]^2+^ parent ion at m/z 785.85. Any strong deviation from this ratio would have indicated problems with the labeling reaction or recovery of individual samples prior to the sample mixing step. Individual iTRAQ labeling reagents (Applied Biosystems, Foster City, CA, USA) were reconstituted in ethanol, added to peptide mixtures derived from the tryptic digestion of IP eluates (Control: iTRAQ 114, Dpl: iTRAQ 115, PrP: iTRAQ 116, Sho: iTRAQ 117) and incubated at room temperature in the dark for 3 h.

### Two-dimensional liquid chromatography

Strong cation exchange (SCX) chromatography was used to achieve peptide fractionation of the complex digest mixture. Samples digested with trypsin were adjusted to 25% acetonitrile and acidified (pH 3.0) by 20-fold dilution in 25% acetonitrile, 20 mM KH_2_PO_4_, pH 3.0. High performance liquid chromatography (HPLC) was carried out using the Ultimate System (Dionex, Sunnyvale, CA, USA) equipped with a microflow calibration cartridge, a Valco injection port and a 180 nL volume UV cell. Separation was achieved on a self-packed 0.5 mm×110 mm Luna SCX column (Phenomenex, Torrance, CA, USA) at a flow rate of 18 µl/min with a steep salt gradient from 0–400 mM NH_4_Cl in 25% acetonitrile, 20 mM KH_2_PO_4_, pH 3.0. Fractions eluted from the SCX column were desalted with C18 Empore (3 M, Minneapolis, MN, USA) stop and go extraction (STAGE) tips and subsequently subjected to nano-flow RP-HPLC using the Ultimate LC system (Dionex, Sunnyvale, CA, USA) equipped with a nanoflow calibration cartridge at a flow rate of 250 nL/min. Peptides were separated on a 75-µm ID self-packed column containing Proteo C12 reverse-phase matrix (Phenomenex) using a 100-min gradient from 2%–34% acetonitrile in water, with 0.1% (wt/vol) formic acid as the ion-pairing agent.

### Electrospray ionization QqTOF mass spectrometry analysis

The column effluent was coupled directly via a fused silica capillary transfer line to a QSTAR XL hybrid quadrupole/time-of-flight tandem mass spectrometer (Applied Biosystems; MDS Sciex, Concord, ON, Canada) equipped with a MicroIonSpray source. The progress of each LC/MS run was monitored by recording the total ion current (TIC) as a function of time for ions in the m/z range 300 to 1800. At 5-s intervals through the gradient, a mass spectrum was acquired for 1 s, followed by one collision-induced dissociation (CID) acquisition of 4 s each on ions selected by preset parameters of the information-dependent acquisition (IDA) method, using nitrogen as the collision gas. Singly-charged ions were excluded from CID selection. The collision energy was adjusted automatically for each CID spectrum using an empirically-optimized formula which considers the charge state and m/z value of the precursor ion.

### Database searches

Peak lists for database searching were created using Mascot Distiller (Version 1; MatrixScience, London, UK). Searches were performed using designated MS/MS data interpretation algorithms within Protein Prospector (Version 4.21.3; University of California, San Francisco, CA, USA) [69] and Mascot (Version 2.2; MatrixScience). Searches considered up to one missed cleavage and charge states ranging from +2 to +4. The analysis of iTRAQ data was assisted by the software program ProteinPilot (Version 2.0; Applied Biosystems; MDS Sciex). For a protein to be listed in the data tables, it had to be identified by all three search algorithms. All proteins listed in the table were identified with high confidence by the application of the following filters: (i) CID spectra with individual confidence scores of less than 80% were not included (the determination of these confidence scores is documented in the Protein Pilot 2.0 Software Help); (ii) assignments to non-iTRAQ-labeled peptides were not considered; (iii) all identifications of proteins had to be based on at least two CID spectra. Raw iTRAQ ratios were corrected for impurity levels of individual reagent lots determined by the manufacturer. In instances where only two peptides supported the identification of a protein, we required the underlying CID spectra to generate a Mascot score indicating a <5% probability that the match could be considered a random event and further confirmed matches by manual interpretation of spectra. As searches were carried out without species restriction, the correct assignment of matches to mouse entries served as an additional internal control. Identifications were confirmed in repeat experiments performed at a two-fold lower scale. It should be noted that the vast majority of proteins were identified with Mascot scores exceeding thresholds conventionally applied for confident identifications. The mass tolerance range between expected and observed masses used for database searches was ±150 ppm for MS peaks, and ±0.15 Da for MS/MS fragment ions. These relatively large thresholds were used to capture more of the low intense peaks that frequently display broader distribution and are therefore assigned with lower mass accuracy. Threshold levels were optimized based on LC/MS/MS datasets of tryptic digests of standard proteins. All samples were searched against the National Center for Biotechnology Information nonredundant database (nrNCBI, release: June 2008) and a ‘decoy’ database in which all entries of the above NCBI database had been inverted. The interactome diagram was constructed using Cytoscape 2.6.0 [70].

### Snowdrop lectin affinity purification

Mice were subjected to time-controlled transcardiac perfusion crosslinking and brain extracts generated as described before [16]. Brain extracts which had been cleared of all insoluble content by high speed centrifugation (100,000×*g*, 1 hr) were added to snowdrop (*Galanthus nivalis*) lectin agarose (Sigma-Aldrich), which had been pre-equilibrated in Lysis buffer (50 mM Tris, pH 8.3, 150 mM NaCl, 0.5% NP-40, 0.5% sodium deoxycholate, 1× Complete Protease Inhibitor), and incubated on an end-over-end turning wheel for 12 h at 4°C. Following extensive washes with lysis buffer, proteins were eluted by incubation of lectin agarose beads with 1× Laemmli sample buffer containing 100 mM DTT. To generate the crosslink reversal series, snowdrop lectin agarose eluate fractions were subjected to 90°C heat treatment for the indicated durations.

### Cell surface biotinylation

N2a and ScN2a cells, grown to 90% confluency, were washed three times with 10 ml of ice-cold PBS and subsequently subjected to biotinylation with 0.5 mg/ml HOOK-Sulfo-NHS-SS-Biotin (Pierce Biotechnology, Rockford, IL, USA) in PBS for a duration of 30 min at 4°C. Residual biotin was quenched by the addition of 100 mM glycine in PBS and further incubation for 15 min. Following cell lysis in detergent containing buffer (0.5% NP40, 0.5% DOC, 150 mM NaCl, 1× Complete Protease Inhibitor Cocktail) the capture of biotinylated proteins on streptavidin agarose (Sigma-Aldrich) occurred during a 12 h incubation. Following extensive washing with lysis buffer, captured proteins were eluted by reducing the disulfide bond within the HOOK-Sulfo-NHS-SS Biotin conjugation reagent in the presence of 100 mM DTT, 62.5 mM Tris, pH 6.8, 2% SDS.

### Proteinase K digestion

Confluent cells were lysed in 10 mM Tris-HCl, pH 8.0; 100 mM NaCl; 0.5% NP-40; 0.5% sodium deoxycholate (DOC). Equal amounts of protein determined using the bicinchoninic acid (BCA) reagent were digested with 20 µg/ml PK (GIBCO, Carlsbad, CA, USA) at a ratio of 1∶25 (w/w protease to protein) for 1 h at 37°C. Reactions were stopped by the addition of 2 mM phenylmethylsulfonyl fluoride (PMSF). Following ultracentrifugation, pellets were resuspended in SDS-loading buffer and subjected to immunoblot analysis.

## Supporting Information

Figure S1 Evidence for PrP^C^ in high-molecular weight protein complexes. Brain extracts from mice subjected to transcardiac perfusion crosslinking were passed over a size exclusion chromatography column. Western blot analysis of eluate fractions with 7A12 antibody documented the presence of PrP^C^ in high-molecular weight complexes which co-eluted together with calibration proteins spanning a molecular weight range of 669 kDa (lane 1) to 440 kDa (lane 3) to 160 kDa (lane 5). Crosslink reversal by 20-min heat treatment of samples caused a shift of PrP^C^ reactive bands to levels of fully glycosylated PrP^C^ consistent with the release of PrP^C^ from crosslinked complexes (lanes 2, 4 and 6). Th, thyroglobulin; Ft, ferritin; Ig, immunoglobulin; Lg, lactoglobulin.
(0.11 MB PDF)

Figure S2 Inhibition of protein disulfide isomerases shows no effect on PrP^Sc^ formation in a subset of ScN2a cell clones. Bacitracin was added at the indicated concentrations to the cell culture medium and left on the ScN2a clone 3 cells for a duration of 2 days. Following cell lysis, protein levels were adjusted and a subset of samples subjected to digestion with proteinase K. All samples were analyzed by Western blotting with a PrP specific antibody. Please note the absence of an effect on PrP^Sc^ levels in this cell clone.
(0.10 MB PDF)

Table S1 Complete quantitative interactome dataset of prion protein family in mouse neuroblastoma cells.
(0.94 MB PDF)
