# Correction: Interactome Analyses Identify Ties of PrP^C^ and Its Mammalian Paralogs to Oligomannosidic N-Glycans and Endoplasmic Reticulum-Derived Chaperones

**DOI:** 10.1371/annotation/9eb11869-6acb-49b0-978e-abedc3cc545a

**Published:** 2009-10-29

**Authors:** Joel C. Watts, Hairu Huo, Yu Bai, Sepehr Ehsani, Amy Hye Won Jeon, Tujin Shi, Nathalie Daude, Agnes Lau, Rebecca Young, Lei Xu, George A. Carlson, David Williams, David Westaway, Gerold Schmitt-Ulms

The fifth author's name was spelled incorrectly. The correct name is: Amy Hye Won Jeon. 

