# Supplementary material for: Interactome Analyses Identify Ties of PrPC and Its Mammalian Paralogs to Oligomannosidic N-Glycans and Endoplasmic Reticulum-Derived Chaperones
Source: PLoS Pathog. 2009 Oct 2;5(10):e1000608. doi: 10.1371/journal.ppat.1000608 (PMC2749441; doi:10.1371/journal.ppat.1000608)

Supplemental Figure 1

Evidence for PrPC in high-molecular weight protein complexes. Brain extracts from mice subjected to transcardiac perfusion crosslinking were passed over a size exclusion chromatography column. Western blot analysis of eluate fractions with 7A12 antibody documented the presence of PrPC in high-molecular weight complexes which co-eluted together with calibration proteins spanning a molecular weight range of 669 kDa (lane 1) to 440 kDa (lane 3) to 160 kDa (lane 5). Crosslink reversal by 20-min heat treatment of samples caused a shift of PrPC reactive bands to levels of fully glycosylated PrPC consistent with the release of PrPC from crosslinked complexes (lanes 2, 4 and 6). Th, thyroglobulin; Ft, ferritin; Ig, immunoglobulin; Lg, lactoglobulin.

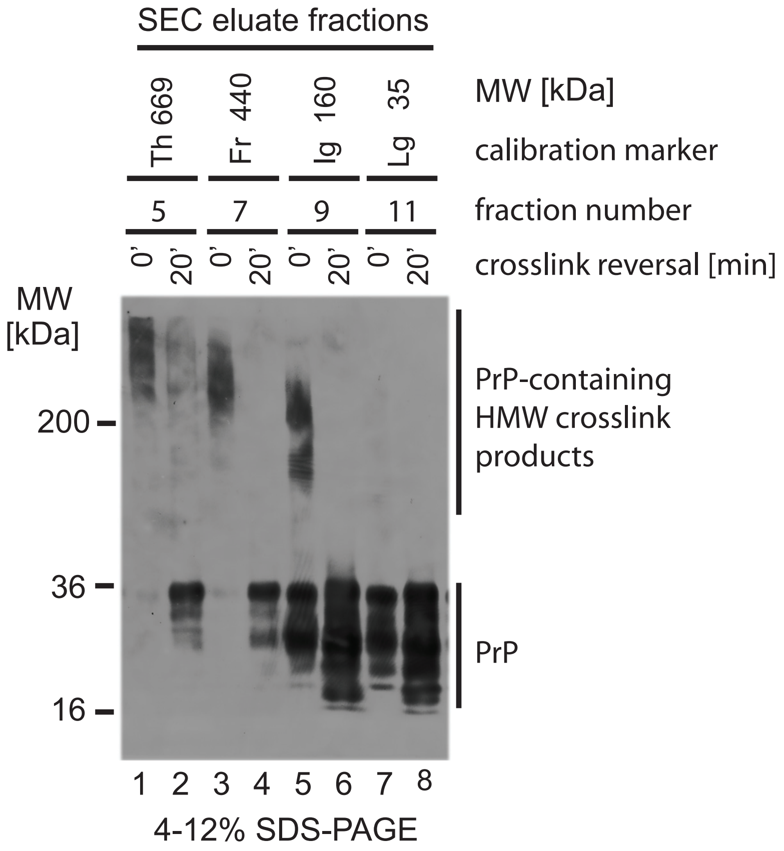

Supplement: Figure S1 — Evidence for PrPC in high-molecular weight protein complexes. Brain extracts from mice subjected to transcardiac perfusion crosslinking were passed over a size exclusion chromatography column. Western blot analysis of eluate fractions with 7A12 antibody documented the presence of PrPC in high-molecular weight complexes which co-eluted together with calibration proteins spanning a molecular weight range of 669 kDa (lane 1) to 440 kDa (lane 3) to 160 kDa (lane 5). Crosslink reversal by 20-min heat treatment of samples caused a shift of PrPC reactive bands to levels of fully glycosylated PrPC consistent with the release of PrPC from crosslinked complexes (lanes 2, 4 and 6). Th, thyroglobulin; Ft, ferritin; Ig, immunoglobulin; Lg, lactoglobulin. (0.11 MB PDF) [file ppat.1000608.s001.pdf]
