# Supplementary material for: Interactome Analyses Identify Ties of PrPC and Its Mammalian Paralogs to Oligomannosidic N-Glycans and Endoplasmic Reticulum-Derived Chaperones
Source: PLoS Pathog. 2009 Oct 2;5(10):e1000608. doi: 10.1371/journal.ppat.1000608 (PMC2749441; doi:10.1371/journal.ppat.1000608)

Supplemental Figure 2

Inhibition of protein disulfide isomerases shows no effect on PrPSc formation in a subset of ScN2a cell clones. Bacitracin was added at the indicated concentrations to the cell culture medium and left on the ScN2a clone 3 cells for a duration of 2 days. Following cell lysis, protein levels were adjusted and a subset of samples subjected to digestion with proteinase K. All samples were analyzed by Western blotting with a PrP specific antibody. Please note the absence of an effect on PrPSc levels in this cell clone.

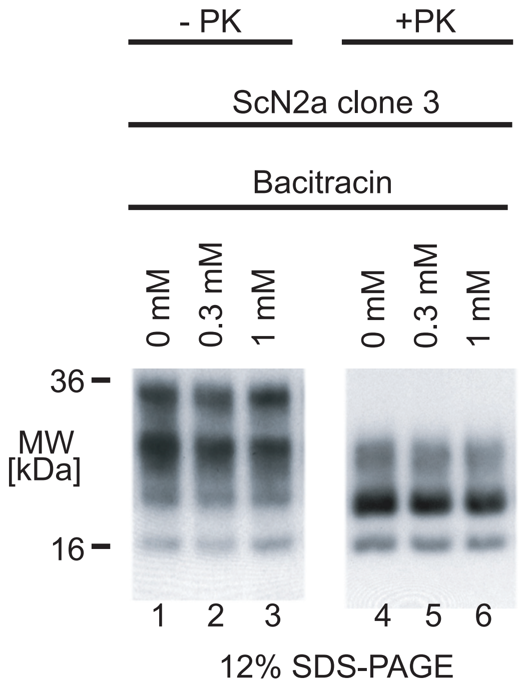

Supplement: Figure S2 — Inhibition of protein disulfide isomerases shows no effect on PrPSc formation in a subset of ScN2a cell clones. Bacitracin was added at the indicated concentrations to the cell culture medium and left on the ScN2a clone 3 cells for a duration of 2 days. Following cell lysis, protein levels were adjusted and a subset of samples subjected to digestion with proteinase K. All samples were analyzed by Western blotting with a PrP specific antibody. Please note the absence of an effect on PrPSc levels in this cell clone. (0.10 MB PDF) [file ppat.1000608.s002.pdf]
