# Supplementary material for: Interactome Analyses Identify Ties of PrPC and Its Mammalian Paralogs to Oligomannosidic N-Glycans and Endoplasmic Reticulum-Derived Chaperones
Source: PLoS Pathog. 2009 Oct 2;5(10):e1000608. doi: 10.1371/journal.ppat.1000608 (PMC2749441; doi:10.1371/journal.ppat.1000608)
Supplement: Table S1 — Complete quantitative interactome dataset of prion protein family in mouse neuroblastoma cells. (0.94 MB PDF) [file ppat.1000608.s003.pdf]

Table S1

Complete quantitative interactome dataset of prion protein family in mouse neuroblastoma cells.

Identified proteins					Identified peptides				Identified parent ions						iTRAQ area (measured)				iTRAQ area (normalized)				iTRAQ area average				iTRAQ ratio			iTRAQ Stdev			
N	%Cov	Accessions	Symbol	Names	Conf	Sequence	Modifications	Cleavages	dMass	Prec MW	Prec m/z	Theor MW	Theor m/z	z	114	115	116	117	114	115	116	117	114	115	116	117	115:114	116:114	117:114	114	115	116	117
1	56.7	IPI:IPI00228113.4	MTHFD1	methylenetetrahydrofolate dehydrogenase	99	AEIDLVCELAK	iTRAQ4plex@N-term; Pyridylethyl(C)@7; iTRAQ4plex(K)@11		0.01	1595.90	532.97	1595.88	532.97	3	67.7	76.1	115.7	93.7	19.2	21.5	32.8	26.5	18.4	22.0	29.1	30.6	1.2	1.6	1.7	4.8	5.2	3.9	5.3
1					99	VLVVGAHGPLEAALQWLFQR	iTRAQ4plex@N-term		0.05	2347.38	783.47	2347.33	783.45	3	14.4	31.4	33.4	36.7	12.4	27.1	28.8	31.7											
1					99	TDPSTLTEEEVR	iTRAQ4plex@N-term		0.01	1519.76	760.89	1519.75	760.88	2	255.0	192.6	222.6	317.5	25.8	19.5	22.5	32.1											
1					99	LVPLVNGVR	iTRAQ4plex@N-term		0.00	1109.71	555.86	1109.70	555.86	2	427.7	644.7	924.9	786.4	15.4	23.2	33.2	28.3											
1					99	DFTLPISDVR	iTRAQ4plex@N-term		0.00	1305.71	653.86	1305.71	653.86	2	138.8	201.6	278.4	258.3	15.8	23.0	31.7	29.4											
1					99	LIAEESVSLLAAALR	iTRAQ4plex@N-term		0.00	1699.00	567.34	1699.00	567.34	3	20.4	30.3	45.0	52.2	13.8	20.5	30.4	35.3											
1					99	EFSEIQLSR	iTRAQ4plex@N-term		0.00	1251.66	626.84	1251.66	626.84	2	615.0	765.8	1087.8	997.5	17.7	22.1	31.4	28.8											
1					99	ASGLVPNVVVLVATVR	iTRAQ4plex@N-term		-0.03	1737.04	580.02	1737.06	580.03	3	4.3	4.0	6.5	5.7	21.1	19.5	31.7	27.7											
1					98	VQLSLLER	iTRAQ4plex@N-term		-0.01	1100.66	551.34	1100.67	551.34	2	221.8	267.6	357.0	277.8	19.7	23.8	31.8	24.7											
1					89	LHPLSPVPSDIEISR	iTRAQ4plex@N-term; His->Gln@2		-0.02	1793.98	599.00	1794.00	599.01	3	10.1	19.1	37.2	38.5	9.7	18.2	35.5	36.7											
1					99	EIGLLADEIEIYGK	iTRAQ4plex@N-term; iTRAQ4plex(K)@14		-0.03	1850.00	617.67	1850.03	617.68	3	8.7	18.0	13.3	12.9	16.4	34.1	25.2	24.3											
1					96	FQFLYDVQLPIVEK	iTRAQ4plex@N-term; iTRAQ4plex(K)@14		-0.02	2026.12	676.38	2026.14	676.39	3	33.9	35.6	79.2	87.6	14.3	15.1	33.5	37.1											
1					99	VHGLTLQISEDSLSNK	iTRAQ4plex@N-term; iTRAQ4plex(K)@16		0.03	2028.14	508.04	2028.11	508.03	4	24.7	42.6	27.6	25.5	20.5	35.4	22.9	21.2											
1					99	STVTIGLVQALTAHLK	iTRAQ4plex@N-term; iTRAQ4plex(K)@16		0.00	1939.17	647.40	1939.17	647.40	3	29.4	34.2	46.9	61.9	17.1	19.8	27.2	35.9											
1					99	DGQPVTAEDLGVTGALTVLMK	iTRAQ4plex@N-term; iTRAQ4plex(K)@21		0.01	2402.31	801.78	2402.30	801.77	3	16.1	23.0	24.6	25.5	18.0	25.8	27.6	28.6											
1					99	DIELSPEAQSK	iTRAQ4plex@N-term; Lys->Arg@11		0.08	1387.79	694.90	1387.71	694.86	2	91.8	144.6	160.3	172.5	16.1	25.4	28.2	30.3											
1					99	DVDGVTDINLGK	iTRAQ4plex@N-term; Lys->Arg@12		0.05	1416.78	709.40	1416.73	709.37	2	17.8	12.0	14.0	17.6	29.0	19.5	22.8	28.7											
1					99	DIELSPEAQSK	iTRAQ4plex@N-term; Lys->Gln@11		0.05	1359.71	680.86	1359.66	680.84	2	57.5	58.7	68.0	77.6	22.0	22.4	26.0	29.6											
1					99	LSGGSPGVPVDK	iTRAQ4plex@N-term; Lys->Gln@12		0.04	1255.69	628.85	1255.65	628.83	2	245.7	207.0	222.1	245.6	26.7	22.5	24.1	26.7											
1					99	TDPSTLTEEEVRK	iTRAQ4plex@N-term; Lys->Gln@13	missed R-K@12	0.05	1647.86	550.29	1647.81	550.28	3	70.7	77.2	88.8	100.3	21.0	22.9	26.4	29.8											
1					99	EIGLLADEIEIYGK	iTRAQ4plex@N-term; Lys->Gln@14		0.02	1705.91	569.64	1705.89	569.64	3	8.9	7.7	9.0	11.1	24.2	21.1	24.5	30.2											
1					99	STVTIGLVQALTAHLK	iTRAQ4plex@N-term; Lys->Gln@16		0.03	1795.06	599.36	1795.03	599.35	3	10.2	16.2	18.7	14.6	17.1	27.2	31.3	24.4											
1					99	NPAFKPVLVVIQAGDDNLMK	iTRAQ4plex@N-term; Lys->Gln@5; Val->Ala@10; iTRAQ4plex(K)@20		0.02	2428.32	608.09	2428.30	608.08	4	30.9	34.7	68.8	64.3	15.5	17.5	34.6	32.3											
1					98	DMNQNLAK	iTRAQ4plex@N-term; Lys->Gln@8		0.07	1076.57	539.29	1076.50	539.26	2	94.0	114.6	243.1	401.7	11.0	13.4	28.5	47.1											
1					96	EVLSLLQEK	iTRAQ4plex@N-term; Lys->Gln@9		0.06	1201.73	601.87	1201.67	601.84	2	41.4	34.9	82.3	89.5	16.7	14.1	33.2	36.1											
1					99	YVLVAGITPSPLGEGK	iTRAQ4plex@N-term; Methyl(S)@10; Lys->Gln@16		0.14	1758.11	587.04	1757.97	587.00	3	8.9	6.5	11.8	12.2	22.6	16.6	29.8	31.0											
2	88.5	IPI:IPI00110850.1	ACTB	actin	99	AGFAGDDAPR	iTRAQ4plex@N-term		0.02	1119.56	560.79	1119.54	560.78	2	196.9	93.9	264.4	160.1	27.5	13.1	37.0	22.4	19.1	21.6	31.8	27.5	1.1	1.7	1.4	8.9	11.7	6.4	10.6
2					99	VAPEEHPVLLTEAPLNPK	Carbamyl@N-term; iTRAQ4plex(K)@18		-0.04	2140.13	714.38	2140.17	714.40	3	5.8	362.6	105.0	66.9	1.1	67.1	19.4	12.4											
2					93	VAPEEHPVLLTEAPLNPK	Formyl@N-term; iTRAQ4plex(K)@18		-0.02	2125.14	709.39	2125.15	709.39	3	22.4	10.2	12.0	7.1	43.3	19.8	23.3	13.7											
2					80	EITALAPSTMK	Glu->pyro-Glu@N-term; iTRAQ4plex(K)@11		0.06	1286.76	644.39	1286.70	644.36	2	174.5	84.3	233.9	206.1	25.0	12.1	33.5	29.5											
2					99	DLYANTVLSGGTTMYPGIADR	iTRAQ4plex@N-term		0.13	2358.29	787.10	2358.16	787.06	3	4.3	11.4	11.8	11.4	11.0	29.3	30.3	29.4											
2					99	QEYDESGPSIVHR	iTRAQ4plex@N-term		0.04	1659.84	554.29	1659.80	554.27	3	11.9	77.6	52.4	38.9	6.6	42.9	29.0	21.5											
2					99	TTGIVMDSGDGVTHTVPIYEGYALPHAILR	iTRAQ4plex@N-term		0.04	3326.75	832.69	3326.71	832.68	4	32.24	26.65	38.09	33.02	24.8	20.5	29.3	25.4											
2					99	TVLSGGTTMYPGIADR	iTRAQ4plex@N-term	cleaved N-T@N-term	0.03	1781.94	594.99	1781.91	594.98	3	31.1	24.8	51.1	40.1	21.1	16.9	34.7	27.2											
2					99	GYSFTTTAER	iTRAQ4plex@N-term		0.00	1275.63	638.82	1275.62	638.82	2	413.9	446.9	1114.6	1056.9	13.6	14.7	36.8	34.9											
2					99	AVFPSIVGRPR	iTRAQ4plex@N-term		-0.01	1341.79	448.27	1341.80	448.27	3	124.4	99.3	166.8	110.2	24.9	19.8	33.3	22.0											
2					99	SYELPDGQVITIGNER	iTRAQ4plex@N-term		-0.02	1933.97	645.66	1933.99	645.67	3	26.5	97.1	72.0	41.4	11.2	41.0	30.4	17.5											
2					99	AVFPSIVGR	iTRAQ4plex@N-term	cleaved R-P@C-term	-0.02	1088.63	545.32	1088.65	545.33	2	689.1	329.8	708.5	807.4	27.2	13.0	27.9	31.9											
2					99	TTGIVMDSGDGVTHTVPIYEGYALPH	iTRAQ4plex@N-term	cleaved H-A@C-term	-0.04	2873.36	958.79	2873.40	958.81	3	51.4	131.2	203.2	279.8	7.7	19.7	30.5	42.0											
2					98	MTQIMFETFNTPAMYVAIQAVLSLYASGR	iTRAQ4plex@N-term		0.07	3396.77	850.20	3396.70	850.18	4	4.0	23.5	12.8	4.9	8.9	52.0	28.3	10.8											
2					99	SYELPDGQVITIGNER	iTRAQ4plex@N-term; Asn->Arg@14		0.01	1976.05	659.69	1976.04	659.69	3	7.4	8.1	14.6	10.2	18.3	20.0	36.3	25.4											
2					99	SYELPDGQVITIGNER	iTRAQ4plex@N-term; Asp->Pro@6		-0.08	1915.93	639.65	1916.01	639.68	3	1.4	4.2	8.3	11.6	5.5	16.6	32.5	45.4											
2					99	SYELPDGQVITIGNER	iTRAQ4plex@N-term; Deamidated(N)@14; Glu->Arg@15		0.02	1962.05	655.02	1962.03	655.02	3	27.9	33.0	40.4	38.4	20.0	23.6	28.9	27.5											
2					99	AVFPSIVGRPR	iTRAQ4plex@N-term; Deamidated(R)@9		-0.03	1342.76	448.59	1342.78	448.60	3	1423.5	796.8	1888.0	1484.0	25.5	14.2	33.8	26.5											
2					99	SYELPDGQVITIGNER	iTRAQ4plex@N-term; Dehydrated(D)@6		-0.05	1915.93	639.65	1915.98	639.67	3	7.3	9.2	22.7	28.3	10.9	13.7	33.6	41.9											
2					81	SYELPDGQVITIGNER	iTRAQ4plex@N-term; Dehydrated(D)@6; Dimethyl(R)@16		0.01	1944.02	649.01	1944.01	649.01	3	5.8	9.5	18.4	11.2	13.0	21.2	41.0	24.8											
2					99	DSYVGDEAQSK	iTRAQ4plex@N-term; Dehydrated(S)@2; Lys->Gln@11		0.04	1323.61	662.81	1323.57	662.79	2	77.8	23.8	78.6	85.7	29.3	8.9	29.6	32.2											
2					99	GYSFTTTAER	iTRAQ4plex@N-term; Dimethyl(R)@10		0.05	1303.71	652.86	1303.65	652.83	2	571.9	173.3	828.1	966.2	22.5	6.8	32.6	38.0											
2					84	AVFPSIVGRPR	iTRAQ4plex@N-term; Dimethyl(R)@11		0.01	1369.85	457.62	1369.83	457.62	3	83.3	48.6	95.4	62.2	28.8	16.8	32.9	21.5											
2					99	SYELPDGQVITIGNER	iTRAQ4plex@N-term; Dimethyl(R)@16		-0.01	1962.01	655.01	1962.02	655.01	3	23.7	17.9	47.7	38.1	18.6	14.0	37.5	29.9											
2					99	DLYANTVLSGGTTMYPGIADR	iTRAQ4plex@N-term; Dimethyl(R)@21		0.03	2386.23	796.42	2386.20	796.41	3	13.4	8.3	0.0	22.8	30.1	18.6	0.0	51.3											
2					99	TTGIVMDSGDGVTHTVPIYEGYALPHAILR	iTRAQ4plex@N-term; Dimethyl(R)@30		-0.03	3354.72	839.69	3354.74	839.69	4	198.5	91.6	204.7	135.7	31.5	14.5	32.5	21.5											
2					99	KDLYANTVLSGGTTMYPGIADR	iTRAQ4plex@N-term; iTRAQ4plex(K)@1		0.04	2630.40	658.61	2630.36	658.60	4	42.8	113.3	97.3	87.9	12.5	33.2	28.5	25.8											
2					99	DSYVGDEAQSK	iTRAQ4plex@N-term; iTRAQ4plex(K)@11		0.04	1485.76	496.26	1485.72	496.25	3	67.4	89.1	95.4	48.0	22.5	29.7	31.8	16.0											
2					99	DSYVGDEAQSKR	iTRAQ4plex@N-term; iTRAQ4plex(K)@11	missed K-R@11	0.04	1641.86	548.29	1641.82	548.28	3	71.1	128.3	175.2	22.0	17.9	32.4	44.2	5.5											
2					99	HQGVMVGMGQK	iTRAQ4plex@N-term; iTRAQ4plex(K)@11		0.00	1458.77	487.26	1458.77	487.26	3	154.3	325.2	397.9	185.1	14.5	30.6	37.5	17.4											
2					99	LDLAGRDLTDYLMK	iTRAQ4plex@N-term; iTRAQ4plex(K)@14	missed R-D@6	0.01	1911.05	638.02	1911.04	638.02	3	137.5	53.7	110.6	36.6	40.6	15.9	32.7	10.8											
2					99	DLTDYLMK	iTRAQ4plex@N-term; iTRAQ4plex(K)@8		0.00	1285.68	643.85	1285.68	643.85	2	348.9	453.4	720.2	396.2	18.2	23.6	37.5	20.7											
2					99	DLYANTVLSGGTTMYPGIADR	iTRAQ4plex@N-term; Leu->Pro@2		-0.02	2342.12	781.71	2342.13	781.72	3	4.1	20.1	21.0	26.2	5.7	28.2	29.4	36.7											
2					99	EITALAPSTMKI	iTRAQ4plex@N-term; Lys->Ala@11; Ile->Ala@12	cleaved I-K@C-term; missed K-I@11	0.01	1318.70	660.36	1318.69	660.35	2	284.9	136.8	444.9	713.2	18.0	8.7	28.2	45.1											
2					99	DSYVGDEAQSK	iTRAQ4plex@N-term; Lys->Arg@11		0.04	1369.67	685.84	1369.62	685.82	2	144.6	72.0	208.1	242.8	21.7	10.8	31.2	36.4											
2					99	EITALAPSTMK	iTRAQ4plex@N-term; Lys->Arg@11		0.01	1332.73	667.37	1332.72	667.37	2	205.4	118.6	351.2	363.8	19.8	11.4	33.8	35.0											
2					90	HQGVMVGMGQK	iTRAQ4plex@N-term; Lys->Arg@11		0.03	1342.70	448.57	1342.67	448.56	3	4.4	5.3	13.2	15.7	11.4	13.7	34.2	40.7											
2					99	VAPEEHPVLLTEAPLNPK	iTRAQ4plex@N-term; Lys->Arg@18		-0.04	2125.12	709.38	2125.17	709.40	3	49.9	60.4	81.1	50.8	20.6	24.9	33.5	21.0											
2					99	HQGVMVGMGQK	iTRAQ4plex@N-term; Lys->Gln@11		0.05	1314.68	439.23	1314.63	439.22	3	86.0	134.0	193.2	194.6	14.2	22.0	31.8	32.0											
2					99	DSYVGDEAQSK	iTRAQ4plex@N-term; Lys->Gln@11		0.04	1341.62	671.82	1341.58	671.80	2	297.1	129.9	454.1	493.7	21.6	9.4	33.0	35.9											
2					96	DSYVGDEAQSKR	iTRAQ4plex@N-term; Lys->Gln@11	missed K-R@11	0.08	1497.76	500.26	1497.68	500.23	3	7.3	11.2	12.1	6.0	19.9	30.6	33.0	16.4											
2					89	EITALAPSTMK	iTRAQ4plex@N-term; Lys->Gln@11		0.07	1304.75	653.38	1304.68	653.35	2	478.7	444.8	758.6	635.2	20.7	19.2	32.7	27.4											
2					99	VAPEEHPVLLTEAPLNPK	iTRAQ4plex@N-term; Lys->Gln@18		0.01	2097.14	700.05	2097.12	700.05	3	90.5	138.4	230.5	196.3	13.8	21.1	35.2	29.9											
2					98	DLTDYLMK	iTRAQ4plex@N-term; Lys->Gln@8		0.02	1141.57	571.79	1141.54	571.78	2	330.3	266.4	955.6	1576.4	10.6	8.5	30.5	50.4											
2					99	YPIEHGIVTNWDDMEK	iTRAQ4plex@N-term; Methyl(E)@4; iTRAQ4plex(K)@16		-0.01	2248.10	750.37	2248.11	750.38	3	70.1	175.1	258.6	145.2	10.8	27.0	39.8	22.4											
2					87	DSYVGDEAQSK	iTRAQ4plex@N-term; Ser->Asp@10; iTRAQ4plex(K)@11		0.01	1513.72	505.58	1513.71	505.58	3	156.0	103.6	199.5	126.2	26.7	17.7	34.1	21.6											
2					92	DSYVGDEAQSK	iTRAQ4plex@N-term; Ser->Val@10; iTRAQ4plex(K)@11		0.01	1497.76	500.26	1497.76	500.26	3	29.6	27.2	31.2	20.4	27.3	25.1	28.8	18.8											
3	54.3	IPI:IPI00122971.1	NCAM1	neural cell adhesion molecule	99	AGEQDASIHLK	iTRAQ4plex@N-term; iTRAQ4plex(K)@11		-0.06	1455.73	486.25	1455.79	486.27	3	78.2	2247.3	349.1	379.6	2.6	73.6	11.4	12.4	3.4	58.6	16.6	21.3	17.3	4.9	6.3	2.4	12.5	5.9	10.8
3					99	QDDGGSPIR	iTRAQ4plex@N-term		0.04	1087.58	544.80	1087.54	544.78	2	32.7	1070.3	189.7	252.6	2.1	69.3	12.3	16.3											
3					99	DIQVIVNVPPTVQAR	iTRAQ4plex@N-term		0.00	1792.03	598.35	1792.03	598.35	3	7.4	74.1	30.8	33.3	5.1	50.9	21.1	22.9											
3					99	FIVLSNNYLQIR	iTRAQ4plex@N-term		-0.01	1622.91	541.98	1622.93	541.98	3	3.2	198.9	44.3	44.2	1.1	68.4	15.2	15.2											
3					99	SLGEESWHFK	iTRAQ4plex@N-term; iTRAQ4plex(K)@10		0.03	1506.80	503.27	1506.77	503.26	3	110.7	878.8	154.1	125.3	8.7	69.3	12.1	9.9											
3					95	TVPNDATQTK	iTRAQ4plex@N-term; iTRAQ4plex(K)@10		0.06	1361.80	454.94	1361.74	454.92	3	4.9	54.0	16.3	12.6	5.6	61.5	18.6	14.4											
3					99	LEGQMGEDGNSIK	iTRAQ4plex@N-term; iTRAQ4plex(K)@13		0.03	1664.86	555.96	1664.83	555.95	3	1.6	99.9	47.1	18.6	1.0	59.8	28.2	11.1											
3					99	GPDPEPTQPGTVK	iTRAQ4plex@N-term; iTRAQ4plex(K)@13		0.01	1609.86	537.63	1609.86	537.63	3	52.3	579.6	182.0	140.5	5.5	60.7	19.1	14.7											
3					98	DESKEPIVEVR	iTRAQ4plex@N-term; iTRAQ4plex(K)@4		0.00	1587.87	530.30	1587.87	530.30	3	9.1	65.2	45.6	20.6	6.5	46.4	32.4	14.7											
3					99	DESKEPIVEVR	iTRAQ4plex@N-term; iTRAQ4plex(K)@4; Dimethyl(R)@11		0.01	1615.91	539.64	1615.90	539.64	3	174.0	879.2	384.1	446.4	9.2	46.7	20.4	23.7											
3					99	ALASEWKPEIR	iTRAQ4plex@N-term; iTRAQ4plex(K)@7		0.05	1586.95	529.99	1586.90	529.97	3	62.7	868.2	231.4	200.1	4.6	63.7	17.0	14.7											
3					92	QEAPSTK	iTRAQ4plex@N-term; iTRAQ4plex(K)@7		0.03	1047.61	524.81	1047.58	524.80	2	1.8	96.4	21.4	17.5	1.3	70.4	15.6	12.7											
3					89	LAALNGK	iTRAQ4plex@N-term; iTRAQ4plex(K)@7		-0.02	973.59	487.80	973.62	487.82	2	478.8	16402.1	2632.3	2333.4	2.2	75.1	12.0	10.7											
3					89	VSSLTLK	iTRAQ4plex@N-term; iTRAQ4plex(K)@7		-0.04	1034.61	518.31	1034.66	518.34	2	340.8	11820.8	1479.0	1345.5	2.3	78.9	9.9	9.0											
3					99	SEPPESEAKPAPTEVK	iTRAQ4plex@N-term; iTRAQ4plex(K)@9; iTRAQ4plex(K)@16		0.01	2127.15	532.79	2127.14	532.79	4	31.8	1181.0	196.6	134.1	2.1	76.5	12.7	8.7											
3					99	LQVDIVPSQGEISVGESK	iTRAQ4plex@N-term; Lys->Arg@18	cleaved S-L@N-term	0.00	2056.10	686.37	2056.09	686.37	3	1.4	23.6	16.9	26.3	2.1	34.6	24.7	38.6											
3					99	GLGEISAATEFK	iTRAQ4plex@N-term; Lys->Gln@12		0.05	1365.74	683.88	1365.69	683.85	2	52.6	285.8	126.6	224.4	7.6	41.5	18.4	32.5											
3					99	EANMEGIVTIMGLKPETR	iTRAQ4plex@N-term; Lys->Gln@14		-0.02	2132.05	711.69	2132.07	711.70	3	12.4	545.3	146.3	288.3	1.2	55.0	14.7	29.1											
3					99	LQVDIVPSQGEISVGESK	iTRAQ4plex@N-term; Lys->Gln@18	cleaved S-L@N-term	0.07	2028.12	677.05	2028.05	677.02	3	1.4	13.5	7.6	7.3	4.7	45.2	25.5	24.6											
3					99	VEPYSSTAQVQFDEPEATGGVPILK	iTRAQ4plex@N-term; Lys->Gln@25		0.09	2805.48	936.17	2805.38	936.13	3	3.1	136.9	22.0	88.9	1.2	54.6	8.8	35.4											
3					99	ASPAPTPTPAGAASPLAAVAAPATDAPQAK	iTRAQ4plex@N-term; Lys->Gln@30		0.10	2811.55	938.19	2811.45	938.16	3	2.1	45.0	13.7	15.6	2.7	58.9	18.0	20.4											
3					99	DESKEPIVEVR	iTRAQ4plex@N-term; Lys->Gln@4		0.02	1443.76	482.26	1443.73	482.25	3	31.4	429.6	164.0	311.6	3.4	45.9	17.5	33.3											
3					99	NAPTPQEFK	iTRAQ4plex@N-term; Lys->Gln@9		0.02	1174.59	588.30	1174.57	588.29	2	122.5	4459.7	962.7	3580.1	1.3	48.9	10.5	39.2											
3					99	SEPPESEAKPAPTEVK	iTRAQ4plex@N-term; Lys->Gln@9; iTRAQ4plex(K)@16		0.08	1983.09	662.04	1983.00	662.01	3	1.7	46.9	14.6	12.8	2.2	61.7	19.2	16.8											
3					99	SEPPESEAKPAPTEVK	iTRAQ4plex@N-term; Lys->Gln@9; Lys->Gln@16		0.12	1838.98	614.00	1838.87	613.96	3	4.6	208.7	40.8	205.1	1.0	45.5	8.9	44.7											
3					99	FFLCQVAGDAK	iTRAQ4plex@N-term; Pyridylethyl(C)@4; iTRAQ4plex(K)@11		-0.03	1590.82	531.28	1590.85	531.29	3	18.1	838.5	94.7	87.6	1.7	80.7	9.1	8.4											
3					99	FFLCQVAGDAK	iTRAQ4plex@N-term; Pyridylethyl(C)@4; Lys->Arg@11		0.04	1474.80	492.61	1474.75	492.59	3	41.3	459.9	187.7	375.6	3.9	43.2	17.6	35.3											
3					99	FFLCQVAGDAK	iTRAQ4plex@N-term; Pyridylethyl(C)@4; Lys->Gln@11		-0.02	1446.69	483.24	1446.71	483.24	3	34.0	1151.0	281.2	685.4	1.6	53.5	13.1	31.9											
3					99	EGEDAVIVCDVVSSLPPTIIWK	iTRAQ4plex@N-term; Pyridylethyl(C)@9; iTRAQ4plex(K)@22		0.02	2762.50	691.63	2762.48	691.63	4	1.4	21.0	6.4	6.2	4.0	60.0	18.2	17.8											
4	99.1	IPI:IPI00850020.1	IGKC	immunoglobulin kappa constant	99	DSTYSMSSTLTLTK	iTRAQ4plex@N-term; iTRAQ4plex(Y)@4; iTRAQ4plex(K)@14		0.02	1966.05	656.36	1966.03	656.35	3	350.0	191.9	146.1	83.9	45.3	24.9	18.9	10.9	22.7	30.0	26.2	21.1	1.3	1.2	0.9	6.2	8.2	5.3	9.5
4					99	TNGNTYLEWYLQKPGQSPK	Carbamyl@N-term; iTRAQ4plex(K)@13; iTRAQ4plex(K)@19	cleaved H-T@N-term	0.06	2554.37	639.60	2554.31	639.58	4	128.8	246.1	131.7	64.9	22.5	43.1	23.0	11.4											
4					99	FSGSGSGTDFTLK	Formyl@N-term; iTRAQ4plex(K)@13		0.00	1474.71	738.36	1474.71	738.36	2	71.6	104.6	118.9	79.5	19.1	27.9	31.7	21.2											
4					84	DSTYSMSSTLTLTKDEYER	Formyl@N-term; Oxidation(M)@6; iTRAQ4plex(K)@14		0.05	2414.15	805.72	2414.09	805.70	3	11.6	19.7	19.5	3.5	21.4	36.3	35.9	6.5											
4					99	QNGVLNSWTDQDSK	Gln->pyro-Glu@N-term; iTRAQ4plex(K)@14		0.04	1717.84	859.93	1717.80	859.91	2	347.3	598.1	466.1	391.3	19.3	33.2	25.9	21.7											
4					98	QNGVLNSWTDQDSK	Gln->pyro-Glu@N-term; Lys->Gln@14		0.12	1573.78	787.90	1573.66	787.84	2	17.7	16.6	10.8	25.5	25.1	23.5	15.2	36.1											
4					93	QNGVLNSWTDQDSK	Gln->pyro-Glu@N-term; Methyl(S)@7; iTRAQ4plex(K)@14		0.06	1731.88	866.95	1731.82	866.92	2	131.4	194.4	128.3	106.1	23.5	34.7	22.9	18.9											
4					99	QNGVLNSWTDQDSK	iTRAQ4plex@N-term; Deamidated(N)@2; iTRAQ4plex(K)@14		0.04	1879.95	627.66	1879.92	627.65	3	106.6	158.0	91.6	49.3	26.3	39.0	22.6	12.1											
4					95	NGNTYLEWYLQKPGQSPK	iTRAQ4plex@N-term; iTRAQ4plex(K)@12; iTRAQ4plex(K)@18	cleaved T-N@N-term	0.01	2554.37	639.60	2554.35	639.60	4	128.8	246.1	131.7	64.9	22.5	43.1	23.0	11.4											
4					99	FSGSGSGTDFTLK	iTRAQ4plex@N-term; iTRAQ4plex(K)@13		-0.06	1590.75	531.26	1590.81	531.28	3	441.5	630.5	570.4	448.8	21.1	30.2	27.3	21.5											
4					99	QNGVLNSWTDQDSK	iTRAQ4plex@N-term; iTRAQ4plex(K)@14		0.02	1878.95	627.32	1878.93	627.32	3	225.4	310.9	178.6	93.3	27.9	38.5	22.1	11.5											
4					98	DSTYSMSSTLTLTK	iTRAQ4plex@N-term; iTRAQ4plex(K)@14		0.03	1821.95	608.33	1821.93	608.32	3	158.0	290.5	188.9	116.7	21.0	38.5	25.1	15.5											
4					99	QNGVLNSWTDQDSKDSTYSMSSTLTLTK	iTRAQ4plex@N-term; iTRAQ4plex(K)@14; iTRAQ4plex(K)@28		-0.01	3538.73	885.69	3538.75	885.69	4	210.3	382.7	232.1	42.3	24.2	44.1	26.8	4.9											
4					99	WKIDGSER	iTRAQ4plex@N-term; iTRAQ4plex(K)@2	missed K-I@2	-0.03	1277.66	426.89	1277.70	426.91	3	749.0	767.8	444.8	117.9	36.0	36.9	21.4	5.7											
4					93	TSTSPIVK	iTRAQ4plex@N-term; iTRAQ4plex(K)@8		-0.06	1119.62	560.82	1119.67	560.84	2	11967.3	18120.0	9801.5	3350.6	27.7	41.9	22.7	7.7											
4					99	QNGVLNSWTDQDSK	iTRAQ4plex@N-term; Lys->Arg@14		0.02	1762.85	588.62	1762.84	588.62	3	43.9	58.2	66.4	87.1	17.2	22.8	26.0	34.1											
4					95	DSTYSMSSTLTLTK	iTRAQ4plex@N-term; Lys->Arg@14		-0.02	1705.81	569.61	1705.83	569.62	3	41.3	40.6	56.6	68.0	20.0	19.7	27.4	32.9											
4					94	TSTSPIVK	iTRAQ4plex@N-term; Lys->Arg@8		0.01	1003.59	502.80	1003.58	502.80	2	624.4	609.1	699.9	619.9	24.5	23.9	27.4	24.3											
4					97	FSGSGSGTDFTLK	iTRAQ4plex@N-term; Lys->Gln@13		0.04	1446.71	724.36	1446.67	724.34	2	760.9	1057.1	1298.9	1561.9	16.3	22.6	27.8	33.4											
4					99	DSTYSMSSTLTLTKDEYER	iTRAQ4plex@N-term; Lys->Gln@14		0.06	2370.12	791.05	2370.07	791.03	3	50.8	89.5	111.9	53.8	16.6	29.2	36.6	17.6											
4					99	DSTYSMSSTLTLTK	iTRAQ4plex@N-term; Lys->Gln@14		0.03	1677.82	560.28	1677.79	560.27	3	41.1	50.9	64.8	75.2	17.7	21.9	27.9	32.4											
4					99	QNGVLNSWTDQDSK	iTRAQ4plex@N-term; Lys->Gln@14		0.02	1734.82	579.28	1734.79	579.27	3	179.9	184.0	155.7	212.4	24.6	25.1	21.3	29.0											
4					99	FSGSGSGTDFTLK	iTRAQ4plex@N-term; Methyl(L)@12; iTRAQ4plex(K)@13		-0.01	1604.82	535.95	1604.83	535.95	3	262.8	252.9	278.3	206.2	26.3	25.3	27.8	20.6											
4					99	QNGVLNSWTDQDSK	iTRAQ4plex@N-term; Methyl(S)@13; Lys->Gln@14		0.01	1748.82	583.95	1748.81	583.94	3	175.2	191.1	175.5	248.4	22.2	24.2	22.2	31.4											
4					99	DSTYSMSSTLTLTK	iTRAQ4plex@N-term; Oxidation(M)@6; iTRAQ4plex(K)@14		0.04	1837.96	613.66	1837.92	613.65	3	83.0	134.7	65.7	73.1	23.3	37.8	18.4	20.5											
4					99	DSTYSMSSTLTLTKDEYER	iTRAQ4plex@N-term; Oxidation(M)@6; Lys->Arg@14		0.10	2414.21	805.74	2414.10	805.71	3	206.1	157.4	253.2	211.7	24.9	19.0	30.6	25.6											
4					98	DSTYSMSSTLTLTK	iTRAQ4plex@N-term; Oxidation(M)@6; Lys->Arg@14		0.03	1721.85	574.96	1721.83	574.95	3	4.4	5.4	10.8	11.5	13.8	16.7	33.7	35.8											
4					91	DSTYSMSSTLTLTK	iTRAQ4plex@N-term; Oxidation(M)@6; Lys->Gln@14		0.03	1693.81	565.61	1693.78	565.60	3	23.2	43.6	48.9	39.1	15.0	28.2	31.6	25.3											
4					98	QNGVLNSWTDQDSK	iTRAQ4plex@N-term; Oxidation(W)@8; Lys->Gln@14		0.04	1750.83	584.62	1750.79	584.60	3	63.8	117.3	76.8	77.0	19.1	35.0	22.9	23.0											
4					99	DVLMTQTPLSLPVSLGDQASISCR	iTRAQ4plex@N-term; Thr->Glu@7; Pyridylethyl(C)@23		0.08	2807.51	702.89	2807.43	702.87	4	17.5	26.3	34.5	33.2	15.7	23.6	31.0	29.8											
4					99	DVLMTQTPLSLPVSLGDQASISCR	iTRAQ4plex@N-term; Thr->Leu@7; Pyridylethyl(C)@23		-0.02	2791.46	698.87	2791.47	698.88	4	54.9	42.1	75.9	44.5	25.2	19.4	34.9	20.5											
5	53.9	IPI:IPI00133903.1	HSP9	heat shock protein 9	99	AMQDAEVSK	iTRAQ4plex@N-term; Lys->Gln@9		0.07	1121.59	561.80	1121.51	561.76	2	67.8	70.2	144.6	110.4	17.3	17.9	36.8	28.1	16.2	22.2	30.9	30.7	1.4	1.9	1.9	4.0	5.4	5.6	7.8
5					99	VLENAEGAR	iTRAQ4plex@N-term		0.01	1101.60	551.81	1101.59	551.80	2	638.0	816.2	1235.1	1136.2	16.7	21.3	32.3	29.7											
5					99	TTPSVVAFTADGER	iTRAQ4plex@N-term		0.00	1593.81	532.28	1593.81	532.28	3	21.8	36.0	42.5	44.4	15.1	24.9	29.4	30.7											
5					99	NAVITVPAYFNDSQR	iTRAQ4plex@N-term		-0.03	1837.92	613.65	1837.94	613.66	3	30.3	62.9	86.7	92.9	11.1	23.1	31.8	34.1											
5					99	VQQTVQDLFGR	iTRAQ4plex@N-term		-0.06	1433.72	717.87	1433.77	717.89	2	772.2	809.5	1541.4	1241.2	17.7	18.5	35.3	28.4											
5					93	DNMALQR	iTRAQ4plex@N-term		0.02	990.52	496.27	990.50	496.26	2	413.3	681.9	699.1	700.0	16.6	27.3	28.0	28.1											
5					91	ETAENYLGHTAK	iTRAQ4plex@N-term; iTRAQ4plex(K)@12		0.04	1620.87	541.30	1620.84	541.29	3	5.8	9.5	6.2	7.8	19.8	32.5	21.1	26.6											
5					99	ASNGDAWVEAHGK	iTRAQ4plex@N-term; iTRAQ4plex(K)@13		0.01	1628.83	543.95	1628.82	543.95	3	185.0	225.1	263.4	198.3	21.2	25.8	30.2	22.7											
5					99	ERVEAVNMAEGIIHDTETK	iTRAQ4plex@N-term; iTRAQ4plex(K)@19	missed R-V@2	0.00	2429.24	608.32	2429.25	608.32	4	61.4	88.6	155.4	92.2	15.4	22.3	39.1	23.2											
5					99	AQFEGIVTDLIK	iTRAQ4plex@N-term; Lys->Gln@12		0.02	1476.81	739.41	1476.79	739.40	2	60.1	77.2	208.5	190.9	11.2	14.4	38.9	35.6											
5					99	SQVFSTAADGQTQVEIK	iTRAQ4plex@N-term; Lys->Gln@17		0.05	1952.01	651.68	1951.96	651.66	3	17.4	20.2	19.4	21.4	22.2	25.8	24.7	27.3											
5					89	ETGVDLTK	iTRAQ4plex@N-term; Lys->Gln@8		0.03	1005.54	503.78	1005.51	503.76	2	26.4	33.4	42.6	45.9	17.8	22.5	28.7	31.0											
5					87	DDIENMVK	iTRAQ4plex@N-term; Lys->Gln@8		0.05	1106.56	554.29	1106.50	554.26	2	35.0	52.7	102.6	221.2	8.5	12.8	24.9	53.7											
6	64.2	IPI:IPI00117312.1	GOT2	Got2 aspartate aminotransferase	99	ASAELALGENNEVLK	No iTRAQ4plex@N-term; iTRAQ4plex(K)@15		-0.04	1700.87	567.96	1700.91	567.98	3	8.5	12.6	14.3	23.1	14.5	21.6	24.5	39.5	17.8	23.2	33.1	25.8	1.3	1.9	1.5	4.8	8.1	6.8	9.2
6					99	IAATILTSPDLR	iTRAQ4plex@N-term		0.02	1413.85	707.93	1413.83	707.92	2	139.9	290.2	343.0	202.6	14.3	29.7	35.2	20.8											
6					99	ILIRPLYSNPPLNGAR	iTRAQ4plex@N-term		0.01	1937.14	646.72	1937.13	646.72	3	102.8	193.2	207.0	143.1	15.9	29.9	32.0	22.1											
6					99	NMGLYGER	iTRAQ4plex@N-term		-0.02	1082.51	542.26	1082.53	542.27	2	2168.2	3655.9	4994.7	4469.7	14.2	23.9	32.7	29.2											
6					99	DAGMQLQGYR	iTRAQ4plex@N-term		-0.02	1281.61	641.81	1281.63	641.82	2	1012.2	2544.6	4371.3	3422.2	8.9	22.4	38.5	30.2											
6					99	FVTVQTISGTGALR	iTRAQ4plex@N-term		-0.04	1592.87	531.96	1592.90	531.97	3	41.4	38.6	70.4	79.3	18.0	16.8	30.7	34.5											
6					99	MNLGVGAYR	iTRAQ4plex@N-term		-0.06	1123.53	562.77	1123.59	562.80	2	4524.9	5628.5	13496.6	12127.4	12.6	15.7	37.7	33.9											
6					99	DAGMQLQGYR	iTRAQ4plex@N-term; Dimethyl(R)@10		-0.01	1309.64	655.83	1309.66	655.84	2	570.8	436.5	1367.3	973.3	17.0	13.0	40.8	29.1											
6					99	ILIRPLYSNPPLNGAR	iTRAQ4plex@N-term; Dimethyl(R)@16		-0.01	1965.15	656.06	1965.16	656.06	3	206.1	155.3	308.8	217.0	23.2	17.5	34.8	24.5											
6					99	ISVAGVTSGNVGYLAHAIHQVTK	iTRAQ4plex@N-term; iTRAQ4plex(K)@23		0.00	2609.45	653.37	2609.45	653.37	4	20.1	47.9	21.3	15.3	19.2	45.8	20.4	14.6											
6					90	SSWWTHVEMGPPDPILGVTEAFKR	iTRAQ4plex@N-term; iTRAQ4plex(K)@23	cleaved A-S@N-term; missed K-R@23	0.10	3027.66	757.92	3027.55	757.90	4	36.0	80.1	65.7	28.6	17.1	38.1	31.2	13.6											
6					99	DDNGKPYVLPSVR	iTRAQ4plex@N-term; iTRAQ4plex(K)@5		0.02	1746.97	583.33	1746.95	583.32	3	87.0	88.2	163.3	89.7	20.3	20.6	38.1	21.0											
6					99	DDNGKPYVLPSVR	iTRAQ4plex@N-term; iTRAQ4plex(K)@5; Dimethyl(R)@13		-0.01	1774.98	592.67	1774.98	592.67	3	485.5	407.4	660.7	406.3	24.8	20.8	33.7	20.7											
6					92	EFSVYMTK	iTRAQ4plex@N-term; iTRAQ4plex(K)@8		0.02	1291.70	646.86	1291.67	646.84	2	131.7	111.4	235.5	100.8	22.7	19.2	40.6	17.4											
6					99	TQLVSNLKK	iTRAQ4plex@N-term; iTRAQ4plex(K)@8; Lys->Gln@9	missed K-K@8	0.05	1317.83	440.29	1317.79	440.27	3	107.2	132.1	210.3	140.8	18.2	22.4	35.6	23.8											
6					94	ASAELALGENNEVLK	iTRAQ4plex@N-term; Lys->Arg@15		0.04	1728.95	577.32	1728.91	577.31	3	10.2	17.2	12.6	39.2	12.8	21.8	15.9	49.5											
6					99	SSWWTHVEMGPPDPILGVTEAFKR	iTRAQ4plex@N-term; Lys->Gln@23	cleaved A-S@N-term; missed K-R@23	0.02	2883.44	721.87	2883.41	721.86	4	19.1	21.6	37.9	23.3	18.8	21.2	37.2	22.9											
6					99	ASSWWTHVEMGPPDPILGVTEAFKR	iTRAQ4plex@N-term; Trp->Asp@5; Lys->Arg@24	missed K-R@24	0.08	2911.52	728.89	2911.44	728.87	4	26.8	16.7	34.6	17.0	28.2	17.6	36.4	17.9											
7	70.8	IPI:IPI00856379.1	ALDOA	fructose-bisphosphate aldolase A	99	AAQEEYIKR	iTRAQ4plex@N-term; iTRAQ4plex(K)@8	missed K-R@8	-0.03	1394.74	465.92	1394.78	465.93	3	257.9	340.9	766.7	241.4	16.0	21.2	47.7	15.0	19.4	22.9	29.3	28.4	1.2	1.5	1.5	9.6	6.4	9.1	11.3
7					99	YTPSGQSGAAASESLFISNHAY	iTRAQ4plex@N-term		0.05	2401.18	801.40	2401.13	801.38	3	20.3	37.0	67.6	35.1	12.7	23.1	42.3	21.9											
7					99	QLLLTADDR	iTRAQ4plex@N-term		0.03	1187.69	594.85	1187.66	594.84	2	314.2	455.1	677.1	1151.0	12.1	17.5	26.1	44.3											
7					99	GVVPLAGTNGETTTQGLDGLSER	iTRAQ4plex@N-term		0.00	2415.24	806.09	2415.24	806.09	3	36.5	52.7	77.7	91.1	14.2	20.4	30.1	35.3											
7					99	FSNEEIAMATVTALRR	iTRAQ4plex@N-term	missed R-R@15	-0.02	1952.01	651.68	1952.03	651.68	3	101.0	140.2	122.2	125.2	20.7	28.7	25.0	25.6											
7					99	FSNEEIAMATVTALR	iTRAQ4plex@N-term		-0.02	1795.90	599.64	1795.93	599.65	3	11.8	11.5	18.6	28.8	16.7	16.3	26.3	40.8											
7					99	GVVPLAGTNGETTTQGLDGLSER	iTRAQ4plex@N-term; Dimethyl(R)@23		-0.01	2443.26	815.43	2443.27	815.43	3	5.9	6.4	7.0	12.1	18.8	20.3	22.3	38.6											
7					99	ADDGRPFPQVIK	iTRAQ4plex@N-term; iTRAQ4plex(K)@12		-0.06	1629.85	544.29	1629.91	544.31	3	326.4	518.9	753.2	320.1	17.0	27.0	39.3	16.7											
7					99	ALQASALK	iTRAQ4plex@N-term; iTRAQ4plex(K)@8		0.01	1088.69	545.35	1088.68	545.35	2	59.1	53.1	55.5	45.2	27.8	24.9	26.1	21.2											
7					84	FSNEEIAMATVTALR	iTRAQ4plex@N-term; Met->His@8		-0.01	1801.94	601.65	1801.94	601.66	3	26.5	27.5	50.6	76.9	14.6	15.1	27.9	42.4											
7					99	ALANSLACQGK	iTRAQ4plex@N-term; Pyridylethyl(C)@8; iTRAQ4plex(K)@11		0.01	1467.82	490.28	1467.81	490.28	3	11.5	27.9	14.4	18.7	15.8	38.5	19.9	25.8											
7					99	ALANSLACQGK	No iTRAQ4plex@N-term; Pyridylethyl(C)@8; iTRAQ4plex(K)@11		0.02	1323.73	442.25	1323.71	442.24	3	804.2	376.2	315.5	221.9	46.8	21.9	18.4	12.9											
8	42.7	IPI:IPI00319992.1	HSPA5	heat shock protein 5	99	AKFEELNMDLFR	iTRAQ4plex@N-term; iTRAQ4plex(K)@2	missed K-F@2	0.02	1799.97	601.00	1799.95	600.99	3	29.6	166.9	105.7	71.4	7.9	44.7	28.3	19.1	7.4	32.9	22.5	37.3	4.5	3.0	5.1	4.3	9.7	7.4	16.8
8					99	ITITNDQNR	iTRAQ4plex@N-term		0.02	1217.67	609.84	1217.65	609.83	2	550.7	2912.2	1354.6	1941.6	8.1	43.1	20.0	28.7											
8					99	ITPSYVAFTPEGER	iTRAQ4plex@N-term		0.02	1709.89	570.97	1709.87	570.97	3	20.9	79.2	76.1	30.4	10.1	38.3	36.8	14.7											
8					99	FEELNMDLFR	iTRAQ4plex@N-term		0.01	1456.73	729.37	1456.71	729.36	2	53.5	523.3	361.2	721.5	3.2	31.5	21.8	43.5											
8					99	SDIDEIVLVGGSTR	iTRAQ4plex@N-term		0.01	1603.86	535.63	1603.85	535.63	3	1.4	17.8	10.9	22.3	2.7	34.0	20.8	42.5											
8					99	KSDIDEIVLVGGSTR	iTRAQ4plex@N-term; iTRAQ4plex(K)@1	missed K-S@1	-0.02	1876.03	626.35	1876.05	626.36	3	76.8	212.6	139.0	108.0	14.3	39.6	25.9	20.1											
8					94	VYEGERPLTK	iTRAQ4plex@N-term; iTRAQ4plex(K)@10		0.01	1478.85	493.96	1478.83	493.95	3	232.5	680.1	339.1	308.6	14.9	43.6	21.7	19.8											
8					99	ELEEIVQPIISK	iTRAQ4plex@N-term; iTRAQ4plex(K)@12		-0.03	1684.95	562.66	1684.99	562.67	3	69.3	282.6	178.7	274.2	8.6	35.1	22.2	34.1											
8					99	TFAPEEISAMVLTK	iTRAQ4plex@N-term; iTRAQ4plex(K)@14		-0.03	1823.97	609.00	1823.99	609.01	3	87.7	265.2	132.6	128.8	14.3	43.2	21.6	21.0											
8					92	NQLTSNPENTVFDAK	iTRAQ4plex@N-term; iTRAQ4plex(K)@15		0.02	1965.03	656.02	1965.00	656.01	3	55.5	150.9	123.1	103.2	12.8	34.9	28.4	23.9											
8					99	IINEPTAAAIAYGLDK	iTRAQ4plex@N-term; iTRAQ4plex(K)@16		-0.01	1947.08	650.03	1947.09	650.04	3	44.1	189.1	173.4	121.8	8.4	35.8	32.8	23.0											
8					99	TFAPEEISAMVLTK	iTRAQ4plex@N-term; Lys->Arg@14		0.05	1707.95	570.32	1707.90	570.31	3	1.4	7.1	12.2	33.0	2.6	13.2	22.7	61.5											
8					95	NQLTSNPENTVFDAK	iTRAQ4plex@N-term; Lys->Arg@15		0.04	1848.95	617.32	1848.91	617.31	3	12.9	29.7	33.1	57.1	9.7	22.4	25.0	43.0											
8					99	NELESYAYSLK	iTRAQ4plex@N-term; Lys->Gln@11		0.05	1459.74	730.88	1459.70	730.85	2	69.3	279.5	290.8	569.8	5.7	23.1	24.0	47.1											
8					91	ELEEIVQPIISK	iTRAQ4plex@N-term; Lys->Gln@12		0.01	1540.85	514.63	1540.85	514.62	3	1.4	4.6	0.0	17.4	6.0	19.6	0.0	74.4											
8					99	TFAPEEISAMVLTK	iTRAQ4plex@N-term; Lys->Gln@14		0.03	1679.89	560.97	1679.86	560.96	3	1.4	26.8	12.8	52.5	1.5	28.7	13.7	56.2											
8					97	IINEPTAAAIAYGLDK	iTRAQ4plex@N-term; Lys->Gln@16		0.02	1802.98	602.00	1802.95	601.99	3	3.6	41.5	17.4	24.2	4.2	47.9	20.0	27.9											
8					99	SQIFSTASDNQPTVTIK	iTRAQ4plex@N-term; Lys->Gln@17		0.04	1980.04	661.02	1979.99	661.00	3	10.8	57.1	33.6	70.0	6.3	33.3	19.6	40.8											
8					99	ETAEAYLGK	iTRAQ4plex@N-term; Lys->Gln@9		0.05	1124.60	563.31	1124.55	563.28	2	20.7	156.3	144.5	485.9	2.6	19.4	17.9	60.2											
8					99	LYGSGGPPPTGEEDTSEKDEL	iTRAQ4plex@N-term; No iTRAQ4plex(K)@18		0.01	2321.08	774.70	2321.07	774.70	3	10.4	78.2	74.9	128.9	3.5	26.7	25.6	44.1											
9	67.3	IPI:IPI00751369.1	LDHA	L-lactate dehydrogenase A chain	99	ATLKDQLIVNLLKEEQAPQNK	iTRAQ4plex(K)@4; iTRAQ4plex(K)@13; Glu->Gln@14; iTRAQ4pl	cleaved M-A@N-term	-0.11	2865.56	717.40	2865.67	717.42	4	10.6	54.4	44.1	34.1	7.4	38.0	30.8	23.8	6.0	26.3	44.0	23.7	4.4	7.3	3.9	8.5	6.6	9.9	11.8
9					99	VTLTPEEEAR	iTRAQ4plex@N-term		0.00	1287.68	644.85	1287.68	644.85	2	333.7	1088.0	2004.1	999.1	7.5	24.6	45.3	22.6											
9					99	LVIITAGAR	iTRAQ4plex@N-term		-0.04	1056.63	529.32	1056.68	529.35	2	516.4	1285.5	3094.2	1383.4	8.2	20.5	49.3	22.0											
9					99	QVVDSAYEVIK	iTRAQ4plex@N-term; iTRAQ4plex(K)@11		-0.01	1537.85	513.62	1537.86	513.63	3	26.8	83.9	101.1	43.4	10.5	32.9	39.6	17.0											
9					99	GEMMDLQHGSLFLK	iTRAQ4plex@N-term; iTRAQ4plex(K)@14		-0.03	1892.95	631.99	1892.97	632.00	3	91.9	561.9	1238.7	714.2	3.5	21.6	47.5	27.4											
9					99	DLADELALVDVMEDK	iTRAQ4plex@N-term; iTRAQ4plex(K)@15		0.00	1963.01	655.34	1963.01	655.34	3	5.1	42.8	61.2	29.9	3.7	30.8	44.0	21.5											
9					99	LLIVSNPVDILTYVAWK	iTRAQ4plex@N-term; iTRAQ4plex(K)@17		0.00	2231.31	744.78	2231.32	744.78	3	4.5	141.5	218.0	102.4	1.0	30.3	46.7	22.0											
9					99	VHPISTMIK	iTRAQ4plex@N-term; iTRAQ4plex(K)@9		0.00	1312.78	438.60	1312.78	438.60	3	27.7	112.8	137.5	83.5	7.7	31.2	38.0	23.1											
9					90	DQLIVNLLK	iTRAQ4plex@N-term; iTRAQ4plex(K)@9		0.00	1342.85	448.62	1342.84	448.62	3	1.4	15.8	24.7	8.7	2.8	31.1	48.9	17.2											
9					99	QVVDSAYEVIK	iTRAQ4plex@N-term; Lys->Arg@11		0.03	1421.79	711.90	1421.76	711.89	2	18.7	90.3	138.8	111.4	5.2	25.1	38.7	31.0											
9					99	QVVDSAYEVIK	iTRAQ4plex@N-term; Lys->Gln@11		0.06	1393.78	697.90	1393.72	697.87	2	40.0	344.4	627.7	494.3	2.7	22.9	41.7	32.8											
9					99	SLNPELGTDADKEQWK	iTRAQ4plex@N-term; Lys->Gln@12; iTRAQ4plex(K)@16		0.09	2118.14	707.05	2118.05	707.02	3	25.9	55.2	177.8	95.5	7.3	15.6	50.2	26.9											
9					97	FIIPNIVK	iTRAQ4plex@N-term; Lys->Gln@8		0.04	1086.70	544.36	1086.66	544.34	2	23.4	101.1	209.2	146.3	4.9	21.1	43.6	30.5											
9					99	DLADELALVDVMEDK	iTRAQ4plex@N-term; Methyl(E)@13; Methyl(D)@14; iTRAQ4plex(K)@15		-0.01	1991.03	664.68	1991.04	664.69	3	5.8	11.9	35.6	27.8	7.1	14.7	43.9	34.3											
9					97	ITVVGVGAVGMACAISILMK	iTRAQ4plex@N-term; Pyridylethyl(C)@13; iTRAQ4plex(K)@20		-0.04	2325.28	582.33	2325.32	582.34	4	1.4	7.2	10.9	7.1	5.3	26.9	41.1	26.7											
9					99	GLYGINEDVFLSVPCILGQNGISDVVK	iTRAQ4plex@N-term; Pyridylethyl(C)@15; iTRAQ4plex(K)@27		0.00	3241.73	811.44	3241.73	811.44	4	15.3	59.6	82.4	29.1	8.2	32.0	44.2	15.6											
9					94	DYCVTANSK	iTRAQ4plex@N-term; Pyridylethyl(C)@3; iTRAQ4plex(K)@9		0.04	1392.74	465.25	1392.70	465.24	3	21.1	70.5	65.4	20.0	11.9	39.8	37.0	11.3											
9					99	VIGSGCNLDSAR	iTRAQ4plex@N-term; Pyridylethyl(C)@6		1.01	1440.74	481.25	1439.73	480.92	3	6.0	69.9	183.7	69.3	1.8	21.3	55.8	21.1											
9					99	VIGSGCNLDSAR	iTRAQ4plex@N-term; Pyridylethyl(C)@6; Ala->Val@11		-0.03	1467.73	490.25	1467.76	490.26	3	201.1	528.2	1347.7	620.9	7.5	19.6	50.0	23.0											
10	61.2	IPI:IPI00230108.6	PDIA3	protein disulfide-isomerase A3	99	ALEQFLQEYFDGNLKR	iTRAQ4plex@N-term; iTRAQ4plex(K)@15	missed K-R@15	0.04	2258.24	753.75	2258.19	753.74	3	47.9	715.3	209.8	169.6	4.2	62.6	18.4	14.8	6.0	46.7	18.8	28.4	7.7	3.1	4.7	5.4	14.3	6.3	9.7
10					99	FVMQEEFSR	iTRAQ4plex@N-term		0.03	1315.67	658.84	1315.64	658.82	2	77.7	451.3	194.2	332.0	7.4	42.8	18.4	31.5											
10					86	ELNDFISYLQR	iTRAQ4plex@N-term		-0.01	1540.80	771.41	1540.80	771.41	2	1.4	49.2	24.4	30.3	1.3	46.7	23.2	28.8											
10					99	FAHTNIESLVK	iTRAQ4plex@N-term; iTRAQ4plex(K)@11		0.00	1545.88	516.30	1545.88	516.30	3	68.1	336.9	114.6	180.4	9.7	48.1	16.4	25.8											
10					99	DLFSDGHSEFLK	iTRAQ4plex@N-term; iTRAQ4plex(K)@12		-0.04	1681.81	561.61	1681.86	561.63	3	248.1	1075.7	515.1	474.4	10.7	46.5	22.3	20.5											
10					99	GFPTIYFSPANKK	iTRAQ4plex@N-term; iTRAQ4plex(K)@12; Lys->Gln@13	missed K-K@12	0.06	1757.00	586.67	1756.94	586.65	3	56.7	235.7	121.8	193.1	9.3	38.8	20.1	31.8											
10					99	SEPIPESNEGPVK	iTRAQ4plex@N-term; iTRAQ4plex(K)@13		0.05	1669.93	557.65	1669.88	557.63	3	12.2	134.4	33.3	24.6	6.0	65.8	16.3	12.0											
10					99	MDATANDVPSPYEVK	iTRAQ4plex@N-term; iTRAQ4plex(K)@15		0.00	1923.95	642.32	1923.95	642.32	3	65.4	391.6	154.7	178.7	8.3	49.5	19.6	22.6											
10					99	EYDDNGEGITIFRPLHLANK	iTRAQ4plex@N-term; iTRAQ4plex(K)@20		0.00	2589.34	648.34	2589.34	648.34	4	42.8	253.1	129.2	133.2	7.7	45.3	23.1	23.9											
10					99	FISDKDASVVGFFR	iTRAQ4plex@N-term; iTRAQ4plex(K)@5		0.04	1875.05	626.02	1875.01	626.01	3	75.8	1167.3	352.5	394.3	3.8	58.7	17.7	19.8											
10					89	GIVPLAK	iTRAQ4plex@N-term; iTRAQ4plex(K)@7		-0.03	984.63	493.32	984.66	493.34	2	667.4	4280.7	1772.4	2351.5	7.4	47.2	19.5	25.9											
10					99	YGVSGYPTLK	iTRAQ4plex@N-term; Lys->Gln@10		0.02	1227.65	614.83	1227.63	614.82	2	98.7	664.6	347.9	984.3	4.7	31.7	16.6	47.0											
10					97	YGVSGYPTLK	iTRAQ4plex@N-term; No iTRAQ4plex(K)@10		0.02	1227.68	614.85	1227.66	614.84	2	16.0	98.5	48.6	139.9	5.3	32.5	16.0	46.2											
10					99	FIQDSIFGLCPH	iTRAQ4plex@N-term; Pyridylethyl(C)@10	cleaved H-M@C-term	0.03	1624.85	542.63	1624.82	542.61	3	9.0	142.5	59.2	167.3	2.4	37.7	15.7	44.3											
10					98	SDVLELTDENFESR	iTRAQ4plex@N-term; Ser->Asp@13	cleaved A-S@N-term	0.05	1824.90	609.31	1824.85	609.29	3	1.4	26.9	10.7	18.0	2.5	47.3	18.7	31.6											
11	58.6	IPI:IPI00308885.6	HSPD1	heat shock 60kDa protein 1	99	ALMLQGVDLLADAVAVTMGPK	iTRAQ4plex@N-term; iTRAQ4plex(K)@21		-0.04	2400.30	801.11	2400.34	801.12	3	34.8	65.1	58.3	41.7	17.4	32.6	29.2	20.9	16.3	19.8	31.6	32.3	1.2	1.9	2.0	3.3	5.6	2.5	6.1
11					99	LVQDVANNTNEEAGDGTTTATVLAR	iTRAQ4plex@N-term		0.00	2703.34	902.12	2703.34	902.12	3	46.6	74.3	117.6	109.8	13.4	21.3	33.8	31.5											
11					96	GANPVEIR	iTRAQ4plex@N-term		0.00	998.56	500.29	998.56	500.29	2	422.1	761.7	992.5	1217.0	12.4	22.4	29.2	35.9											
11					90	VTDALNATR	iTRAQ4plex@N-term		-0.01	1103.60	552.80	1103.61	552.81	2	2478.7	3583.9	5143.8	4110.6	16.2	23.4	33.6	26.8											
11					99	LVQDVANNTNEEAGDGTTTATVLAR	iTRAQ4plex@N-term; Dimethyl(R)@25		0.02	2731.39	911.47	2731.37	911.47	3	41.3	41.8	66.4	53.6	20.3	20.6	32.7	26.4											
11					99	GVMLAVDAVIAELK	iTRAQ4plex@N-term; iTRAQ4plex(K)@14		-0.03	1715.98	573.00	1716.01	573.01	3	5.9	5.2	11.1	15.7	15.6	13.6	29.3	41.4											
11					99	DIGNIISDAMK	iTRAQ4plex@N-term; Lys->Gln@11		0.07	1319.72	660.87	1319.65	660.83	2	69.7	52.3	95.9	105.3	21.6	16.2	29.7	32.6											
11					99	NAGVEGSLIVEK	iTRAQ4plex@N-term; Lys->Gln@12		0.07	1358.79	680.40	1358.72	680.37	2	41.1	49.0	111.9	112.4	13.1	15.6	35.6	35.7											
11					99	IQEITEQLDITTSEYEK	iTRAQ4plex@N-term; Lys->Gln@17		0.04	2183.10	728.71	2183.06	728.69	3	7.3	9.4	18.7	19.5	13.4	17.1	34.0	35.5											
11					99	TALLDAAGVASLLTTAEAVVTEIPKEEK	iTRAQ4plex@N-term; Lys->Gln@25; iTRAQ4plex(K)@28		-0.03	3127.68	782.93	3127.71	782.93	4	13.3	10.5	20.2	25.1	19.3	15.1	29.2	36.3											
12	67.9	IPI:IPI00119478.1	TMOD3	tropomodulin-3	99	DLDEDELLGK	iTRAQ4plex@N-term; No iTRAQ4plex(K)@10		0.03	1289.68	645.85	1289.65	645.83	2	148.9	12.4	60.0	77.7	49.8	4.1	20.1	26.0	25.8	20.7	29.2	24.2	0.8	1.1	0.9	9.1	11.8	4.7	11.5
12					99	QLETVLDDLDPENALLPAGFR	iTRAQ4plex@N-term		0.00	2469.29	824.10	2469.29	824.10	3	45.1	73.2	72.2	36.0	19.9	32.3	31.9	15.9											
12					99	FGYQFTQQGPR	iTRAQ4plex@N-term		0.00	1471.73	491.58	1471.73	491.58	3	250.9	175.7	295.8	220.8	26.6	18.6	31.4	23.4											
12					99	SLNMESNFITGAGVLALIDALR	iTRAQ4plex@N-term		0.00	2448.31	817.11	2448.32	817.11	3	15.9	29.9	24.8	17.4	18.1	34.0	28.2	19.7											
12					91	DRDDYVPYTGEK	iTRAQ4plex@N-term; iTRAQ4plex(K)@12	missed R-D@2	0.04	1744.89	582.64	1744.85	582.62	3	46.5	79.6	61.3	38.6	20.6	35.2	27.1	17.1											
12					99	SNDPVAVAFADMLK	iTRAQ4plex@N-term; iTRAQ4plex(K)@14		-0.02	1764.91	589.31	1764.93	589.32	3	19.4	10.0	22.1	14.2	29.5	15.3	33.6	21.7											
12					95	AANAITK	iTRAQ4plex@N-term; iTRAQ4plex(K)@7		0.03	975.62	488.82	975.60	488.81	2	374.7	233.9	314.7	120.3	35.9	22.4	30.2	11.5											
12					94	LSESELK	iTRAQ4plex@N-term; iTRAQ4plex(K)@7		-0.01	1092.62	547.32	1092.63	547.32	2	326.6	230.5	326.7	118.7	32.6	23.0	32.6	11.8											
12					94	TLEANTHVK	iTRAQ4plex@N-term; iTRAQ4plex(K)@9		0.02	1299.76	434.26	1299.74	434.25	3	83.2	127.5	55.9	19.3	29.1	44.6	19.5	6.7											
12					97	DNETLMELK	iTRAQ4plex@N-term; Lys->Arg@9		0.01	1263.64	632.83	1263.63	632.82	2	92.5	43.1	178.4	220.3	17.3	8.1	33.4	41.2											
12					99	MLEENTNILK	iTRAQ4plex@N-term; Lys->Gln@10		0.02	1347.71	674.86	1347.68	674.85	2	126.0	97.3	212.7	228.6	19.0	14.6	32.0	34.4											
12					93	QQLGTSVELEMAK	iTRAQ4plex@N-term; Lys->Gln@13		0.02	1576.80	526.61	1576.79	526.60	3	31.1	24.9	46.8	72.9	17.7	14.2	26.6	41.5											
12					99	ILPVFDEPPNPTNVEESLKR	iTRAQ4plex@N-term; Lys->Gln@19	missed K-R@19	0.02	2437.28	813.43	2437.26	813.43	3	402.3	211.9	555.9	448.5	24.9	13.1	34.3	27.7											
12					80	DNETLMELK	iTRAQ4plex@N-term; Lys->Gln@9		0.03	1235.61	618.81	1235.58	618.80	2	344.3	177.4	474.7	671.7	20.6	10.6	28.5	40.3											

13	66.8	IPI:IPI00115564.5	SLC25A4	ADP/ATP translocase 1	99	DFLAGGIAAAVSK	iTRAQ4plex@N-term; Lys->Gln@13		0.01	1362.74	682.38	1362.73	682.37	2	94.7	81.3	126.6	143.6	21.2	18.2	28.4	32.2	15.4	26.4	26.8	31.4	1.7	1.7	2.0	6.2	5.3	4.4	8.8
13					99	EQGFLSFWR	iTRAQ4plex@N-term		-0.04	1312.62	657.32	1312.67	657.34	2	578.5	1549.8	1531.3	2576.9	9.3	24.9	24.6	41.3
13					99	QIFLGGVDR	iTRAQ4plex@N-term		-0.08	1147.57	574.79	1147.65	574.83	2	2787.7	6031.2	6406.7	11092.0	10.6	22.9	24.3	42.1
13					98	GNLANVIR	iTRAQ4plex@N-term		-0.03	999.57	500.79	999.59	500.80	2	3123.1	7182.0	5566.6	11200.2	11.5	26.5	20.6	41.4
13					99	LLLQVQHASK	iTRAQ4plex@N-term; iTRAQ4plex(K)@10		0.02	1423.89	475.64	1423.88	475.63	3	370.6	358.2	319.2	259.6	28.3	27.4	24.4	19.9
13					99	YFPTQALNFAFK	iTRAQ4plex@N-term; iTRAQ4plex(K)@12		-0.02	1733.92	578.98	1733.94	578.99	3	28.4	81.0	52.8	51.9	13.3	37.9	24.7	24.2
13					91	LAADVGK	iTRAQ4plex@N-term; iTRAQ4plex(K)@7		0.00	960.58	481.30	960.58	481.30	2	78.8	121.3	144.8	89.4	18.1	27.9	33.3	20.6
13					99	YFPTQALNFAFK	iTRAQ4plex@N-term; Lys->Gln@12		0.06	1589.86	795.94	1589.80	795.91	2	53.5	104.7	100.0	111.3	14.5	28.3	27.1	30.1
13					99	GADIMYTGTLDCWR	iTRAQ4plex@N-term; Pyridylethyl(C)@12		-0.03	1849.83	617.62	1849.86	617.63	3	17.3	36.3	51.5	47.7	11.3	23.8	33.7	31.2
14	68.0	IPI:IPI00462072.3	ENO1	alpha-enolase	99	AAVPSGASTGIYEALELR	iTRAQ4plex@N-term		-0.05	1947.98	650.34	1948.04	650.35	3	15.8	38.7	71.8	53.4	8.8	21.5	40.0	29.7	17.2	21.7	32.3	28.9	1.3	1.9	1.7	3.9	6.7	6.2	5.2
14					99	LAMQEFMILPVGASSFR	iTRAQ4plex@N-term		0.00	2040.06	681.03	2040.07	681.03	3	14.3	18.5	19.7	24.2	18.6	24.1	25.7	31.5
14					99	LAQSNGWGVMVSHR	iTRAQ4plex@N-term		-0.02	1684.84	562.62	1684.86	562.63	3	77.6	173.9	197.1	178.2	12.4	27.7	31.5	28.4
14					99	AAVPSGASTGIYEALELR	iTRAQ4plex@N-term; Dimethyl(R)@18		-0.01	1976.06	659.69	1976.07	659.70	3	12.0	4.3	26.0	19.5	19.4	6.9	42.1	31.6
14					99	GVSQAVEHINK	iTRAQ4plex@N-term; iTRAQ4plex(K)@11		-0.03	1468.79	490.60	1468.82	490.62	3	354.9	385.1	590.6	409.1	20.4	22.1	33.9	23.5
14					99	YITPDQLADLYK	iTRAQ4plex@N-term; iTRAQ4plex(K)@12		-0.04	1726.90	576.64	1726.94	576.65	3	378.2	423.2	421.9	403.0	23.3	26.0	25.9	24.8
14					99	HIADLAGNPEVILPVPAFNVINGGSHAGNK	iTRAQ4plex@N-term; iTRAQ4plex(K)@30		0.01	3308.79	662.77	3308.79	662.76	5	34.9	60.2	70.9	49.0	16.2	28.0	33.0	22.8
14					99	YITPDQLADLYK	iTRAQ4plex@N-term; Lys->Arg@12		0.04	1610.89	806.45	1610.84	806.43	2	134.1	71.7	233.2	179.1	21.7	11.6	37.7	29.0
14					99	DATNVGDEGGFAPNILENK	iTRAQ4plex@N-term; Lys->Arg@19		0.01	2132.04	711.69	2132.03	711.68	3	5.8	12.6	13.6	14.0	12.5	27.5	29.6	30.4
14					99	LMIEMDGTENK	iTRAQ4plex@N-term; Lys->Gln@11		0.04	1423.68	712.85	1423.64	712.83	2	280.2	285.5	643.4	296.9	18.6	19.0	42.7	19.7
14					99	YITPDQLADLYK	iTRAQ4plex@N-term; Lys->Gln@12		0.04	1582.84	792.43	1582.80	792.41	2	98.8	172.5	150.3	152.9	17.2	30.0	26.2	26.6
14					95	GNPTVEVDLYTAK	iTRAQ4plex@N-term; Lys->Gln@13		0.04	1549.82	517.61	1549.77	517.60	3	4.4	4.0	7.0	10.5	17.1	15.4	27.1	40.5
14					99	DATNVGDEGGFAPNILENK	iTRAQ4plex@N-term; Lys->Gln@19		0.07	2104.06	702.36	2103.98	702.33	3	16.2	19.1	28.6	30.2	17.2	20.3	30.4	32.1
14					99	LAMQEFMILPVGASSFR	iTRAQ4plex@N-term; Oxidation(M)@7		-0.02	2056.04	686.35	2056.06	686.36	3	5.9	7.9	9.0	11.5	17.1	23.1	26.3	33.6
15	46.6	IPI:IPI00454008.1	SHMT2	serine hydroxymethyltransferase	99	GFPMPGFDER	iTRAQ4plex@N-term		-0.01	1295.60	648.81	1295.61	648.81	2	664.3	713.0	1144.1	1339.7	17.2	18.5	29.6	34.7	15.7	22.8	31.0	30.5	1.5	2.0	1.9	4.9	10.3	4.8	7.1
15					99	QQVEQFAR	iTRAQ4plex@N-term		-0.01	1148.60	575.31	1148.61	575.31	2	1530.5	1288.8	2742.1	3246.7	17.4	14.6	31.1	36.9
15					99	LNPQTGLIDYDQLALTAR	iTRAQ4plex@N-term		-0.02	2145.14	716.05	2145.16	716.06	3	18.7	40.4	30.7	33.7	15.2	32.7	24.9	27.3
15					92	LGAPALTSR	iTRAQ4plex@N-term		0.01	1028.62	515.32	1028.61	515.31	2	187.2	615.9	465.9	383.7	11.3	37.3	28.2	23.2
15					91	EIPYTFEDR	iTRAQ4plex@N-term		0.01	1312.65	657.33	1312.64	657.33	2	83.8	165.9	307.1	222.6	10.8	21.3	39.4	28.6
15					87	SGLIFYR	iTRAQ4plex@N-term		-0.01	998.56	500.29	998.57	500.29	2	143.5	336.1	285.1	246.0	14.2	33.3	28.2	24.3
15					91	VVDFIDEGVNIGLEVK	iTRAQ4plex@N-term; iTRAQ4plex(K)@16		-0.02	2033.10	678.71	2033.13	678.72	3	22.0	29.1	34.2	36.5	18.1	23.9	28.1	29.9
15					99	GYSLVSGGTDTHLVLVDLRPK	iTRAQ4plex@N-term; iTRAQ4plex(K)@21		0.00	2514.40	629.61	2514.40	629.61	4	82.0	182.9	269.0	211.1	11.0	24.6	36.1	28.3
15					99	VLELVSITANK	iTRAQ4plex@N-term; Lys->Gln@11		0.06	1329.82	665.92	1329.76	665.89	2	53.8	89.3	95.3	101.1	15.9	26.3	28.1	29.8
15					99	GLELIASENFCSR	iTRAQ4plex@N-term; Pyridylethyl(C)@11		0.04	1686.89	563.30	1686.85	563.29	3	218.4	144.3	219.8	192.4	28.2	18.6	28.4	24.8
15					99	YYGGAEVVDEIELLCQR	iTRAQ4plex@N-term; Pyridylethyl(C)@15		2.07	2207.16	736.73	2205.09	736.04	3	6.1	0.0	17.4	21.5	13.5	0.0	38.7	47.8
16	39.1	IPI:IPI00131622.1	DPL	doppel	99	HCDFWLER	iTRAQ4plex@N-term; Pyridylethyl(C)@2		-0.03	1353.61	452.21	1353.64	452.22	3	135.7	2028.4	76.8	37.6	6.0	89.0	3.4	1.6	2.0	92.5	3.0	2.5	46.7	1.5	1.3	1.7	3.7	1.6	1.5
16					99	VAENRPGAF	iTRAQ4plex@N-term	cleaved F-I@C-term	0.00	1103.58	552.80	1103.58	552.80	2	36.9	4866.0	87.6	146.6	0.7	94.7	1.7	2.9
16					99	VLPSSGGQITEAR	iTRAQ4plex@N-term		-0.01	1457.79	729.90	1457.80	729.91	2	4.3	8100.0	100.0	48.5	0.1	98.1	1.2	0.6
16					99	LDIDFGAEGNR	iTRAQ4plex@N-term		-0.02	1349.65	675.83	1349.67	675.84	2	27.0	1965.0	54.4	36.3	1.3	94.3	2.6	1.7
16					99	VLPSSGGQITEAR	iTRAQ4plex@N-term; Ala->Val@12		0.01	1485.84	496.29	1485.83	496.28	3	32.8	1837.2	50.0	123.3	1.6	89.9	2.4	6.0
16					99	VLPSSGGQITEARVAENRPGAFIKQGR	iTRAQ4plex@N-term; Asn->His@17; Lys->Glu@24	missed R-V@13; missed K-Q@24	0.00	3005.59	752.41	3005.59	752.41	4	76.7	1757.6	79.2	40.9	3.9	89.9	4.1	2.1
16					99	VLPSSGGQITEAR	iTRAQ4plex@N-term; Deamidated(Q)@8; Dimethyl(R)@13		0.06	1486.87	496.63	1486.81	496.61	3	36.7	3195.1	155.2	115.8	1.0	91.2	4.4	3.3
16					99	KVLPSSGGQITEAR	iTRAQ4plex@N-term; iTRAQ4plex(K)@1	missed K-V@1	-0.02	1729.97	577.66	1729.99	577.67	3	16.6	908.9	40.7	32.3	1.7	91.0	4.1	3.2
16					99	KLDIDFGAEGNR	iTRAQ4plex@N-term; iTRAQ4plex(K)@1	missed K-L@1	-0.04	1621.83	541.62	1621.87	541.63	3	26.9	989.0	71.1	41.9	2.4	87.6	6.3	3.7
16					99	KVLPSSGGQITEAR	iTRAQ4plex@N-term; iTRAQ4plex(K)@1; HexNAc(T)@11	missed K-V@1	0.02	1933.09	645.37	1933.07	645.36	3	54.1	1967.2	69.5	42.2	2.5	92.2	3.3	2.0
16					99	VAENRPGAFIK	iTRAQ4plex@N-term; iTRAQ4plex(K)@11		0.96	1489.83	497.62	1488.87	497.30	3	1.4	7397.0	0.0	45.9	0.0	99.4	0.0	0.6
16					99	VAENRPGAFIK	iTRAQ4plex@N-term; Lys->Arg@11		-0.05	1372.72	687.37	1372.77	687.39	2	70.7	6798.6	106.9	116.0	1.0	95.9	1.5	1.6
16					92	KVLPSSGGQITEAR	iTRAQ4plex@N-term; Lys->Gln@1	missed K-V@1	0.05	1585.90	529.64	1585.85	529.63	3	24.1	605.4	25.6	24.9	3.5	89.0	3.8	3.7
17	37.4	IPI:IPI00468481.2	ATP5B	ATP synthase subunit beta	99	AIAELGIYPAVDPLDSTSR	iTRAQ4plex@N-term		-0.03	2131.10	711.37	2131.13	711.38	3	20.4	31.2	62.2	46.1	12.8	19.5	38.9	28.9	17.4	24.6	30.5	27.5	1.4	1.8	1.6	4.8	5.8	7.3	3.6
17					99	TIAMDGTEGLVR	iTRAQ4plex@N-term		0.02	1405.76	469.59	1405.74	469.59	3	30.9	34.7	30.0	36.6	23.4	26.3	22.7	27.7
17					99	LVLEVAQHLGESTVR	iTRAQ4plex@N-term		0.02	1794.03	599.02	1794.01	599.01	3	92.1	131.2	217.9	196.9	14.4	20.6	34.1	30.9
17					99	FLSQPFQVAEVFTGHMGK	iTRAQ4plex@N-term; Oxidation(M)@16; iTRAQ4plex(K)@18		0.06	2326.26	776.43	2326.20	776.41	3	66.9	111.9	92.6	78.4	19.1	32.0	26.5	22.4
18	53.8	IPI:IPI00323592.2	MDH2	malate dehydrogenase	99	FVFSLVDAMNGK	iTRAQ4plex@N-term; Lys->Gln@12		0.09	1470.82	736.42	1470.73	736.37	2	39.0	78.1	183.8	307.6	6.4	12.8	30.2	50.6	15.9	22.0	31.6	30.4	1.4	2.0	1.9	7.5	8.1	3.7	13.9
18					99	LTLYDIAHTPGVAADLSHIETR	iTRAQ4plex@N-term		-0.02	2536.32	635.09	2536.34	635.09	4	10.1	19.8	20.3	14.3	15.7	30.7	31.5	22.2
18					98	IFGVTTLDIVR	iTRAQ4plex@N-term		-0.01	1376.80	689.41	1376.81	689.41	2	45.4	61.1	109.2	60.4	16.4	22.1	39.5	21.9
18					99	VAVLGASGGIGQPLSLLLK	iTRAQ4plex@N-term; iTRAQ4plex(K)@19		-0.05	2080.23	694.42	2080.29	694.44	3	50.4	40.4	48.2	29.4	29.9	24.0	28.6	17.4
18					93	ITPFEEK	iTRAQ4plex@N-term; iTRAQ4plex(K)@7		-0.01	1150.63	576.32	1150.65	576.33	2	4279.9	4410.2	6004.1	2983.9	24.2	24.9	34.0	16.9
18					91	MIAEAIPELK	iTRAQ4plex@N-term; Lys->Gln@10		0.05	1257.72	629.87	1257.68	629.85	2	135.6	139.1	362.2	534.5	11.6	11.9	30.9	45.6
18					89	ANTFVAELK	iTRAQ4plex@N-term; Lys->Gln@9		0.04	1135.64	568.83	1135.60	568.81	2	83.2	124.1	213.9	330.3	11.1	16.5	28.5	43.9
18					99	GCDVVVIPAGVPR	iTRAQ4plex@N-term; Pyridylethyl(C)@2		0.00	1529.85	510.96	1529.85	510.96	3	65.3	116.9	84.6	51.7	20.5	36.7	26.6	16.2
18					99	TIIPLISQCTPK	iTRAQ4plex@N-term; Pyridylethyl(C)@9; Lys->Arg@12		0.03	1589.94	530.99	1589.91	530.98	3	23.0	50.0	115.6	149.1	6.8	14.8	34.2	44.1
18					81	TIIPLISQCTPK	iTRAQ4plex@N-term; Pyridylethyl(C)@9; Lys->Gln@12		0.06	1561.92	521.65	1561.87	521.63	3	35.0	55.7	69.5	54.6	16.3	25.9	32.4	25.4
19	50.5	IPI:IPI00117352.1	TUBB5	tubulin beta-5	99	AILVDLEPGTMDSVR	iTRAQ4plex@N-term		-0.05	1758.88	587.30	1758.93	587.32	3	32.5	32.0	59.3	50.6	18.6	18.4	34.0	29.0	17.5	21.2	31.6	29.7	1.2	1.8	1.7	2.5	6.5	6.8	6.0
19					99	GHYTEGAELVDSVLDVVR	iTRAQ4plex@N-term		1.93	2104.01	702.34	2102.08	701.70	3	150.7	280.5	297.9	350.3	14.0	26.0	27.6	32.5
19					99	LAVNMVPFPR	iTRAQ4plex@N-term		0.01	1286.74	644.38	1286.73	644.37	2	1633.8	1249.0	3496.6	2258.8	18.9	14.5	40.5	26.1
19					99	IREEYPDR	iTRAQ4plex@N-term	missed R-E@2	0.01	1220.64	407.89	1220.63	407.88	3	94.5	90.8	161.8	90.9	21.6	20.7	36.9	20.7
19					99	LHFFMPGFAPLTSR	iTRAQ4plex@N-term		-0.01	1763.92	588.98	1763.93	588.98	3	27.5	54.2	42.9	42.3	16.5	32.5	25.7	25.3
19					92	YLTVAAVFR	iTRAQ4plex@N-term		0.00	1182.69	592.35	1182.69	592.35	2	160.9	156.3	180.5	344.8	19.1	18.6	21.4	40.9
19					99	MAVTFIGNSTAIQELFK	iTRAQ4plex@N-term; iTRAQ4plex(K)@17		0.00	2157.17	720.07	2157.17	720.07	3	10.2	17.6	19.0	21.2	14.9	25.9	27.9	31.2
19					99	LHFFMPGFAPLTSR	iTRAQ4plex@N-term; Ser->Asp@13		0.07	1792.00	598.34	1791.93	598.32	3	20.7	16.5	47.7	39.2	16.7	13.3	38.4	31.5
20	28.6	IPI:IPI00554929.2	HSP90AB1	heat shock protein 90-beta	99	ADLINNLGTIAK	iTRAQ4plex@N-term; No iTRAQ4plex(K)@12		-0.01	1385.79	693.90	1385.80	693.91	2	50.1	55.1	136.1	148.3	12.8	14.2	34.9	38.1	11.5	15.6	33.4	39.6	1.4	2.9	3.5	3.7	3.2	5.0	8.3
20					99	YHTSQSGDEMTSLSEYVSR	iTRAQ4plex@N-term		0.09	2320.13	774.38	2320.04	774.35	3	54.6	52.4	83.4	65.8	21.3	20.5	32.5	25.7
20					99	GVVDSEDLPLNISR	iTRAQ4plex@N-term		0.04	1656.92	829.47	1656.88	829.45	2	200.9	257.1	658.0	595.0	11.7	15.0	38.5	34.8
20					99	EDQTEYLEER	iTRAQ4plex@N-term		0.02	1454.69	728.35	1454.66	728.34	2	293.0	530.6	1270.0	1111.5	9.1	16.6	39.6	34.7
20					99	ELISNASDALDK	iTRAQ4plex@N-term; Lys->Arg@12		0.06	1446.80	724.41	1446.74	724.38	2	47.2	47.2	106.6	224.3	11.1	11.1	25.1	52.7
20					91	NPDDITQEEYGEFYK	iTRAQ4plex@N-term; Lys->Arg@15		0.04	2018.94	673.99	2018.90	673.97	3	5.8	9.6	16.5	22.0	10.8	17.8	30.6	40.8
20					99	DNSTMGYMMAK	iTRAQ4plex@N-term; Lys->Gln@11		0.05	1391.61	696.81	1391.56	696.79	2	43.2	92.3	166.3	155.7	9.4	20.2	36.3	34.0
20					99	ELISNASDALDK	iTRAQ4plex@N-term; Lys->Gln@12		0.03	1418.73	710.37	1418.70	710.36	2	180.8	350.0	615.6	1218.4	7.6	14.8	26.0	51.5
20					97	YESLTDPSK	iTRAQ4plex@N-term; Lys->Gln@9		0.04	1182.60	592.31	1182.55	592.28	2	131.0	145.7	396.2	547.2	10.7	11.9	32.5	44.9
20					96	YIDQEELNK	iTRAQ4plex@N-term; Lys->Gln@9		0.04	1294.66	648.34	1294.62	648.32	2	45.4	61.7	170.6	177.1	10.0	13.6	37.5	38.9
21	23.1	IPI:IPI00466069.3	EEF2	elongation factor 2	99	EGIPALDNFLDKL	iTRAQ4plex@N-term; iTRAQ4plex(K)@12	missed K-L@12	-0.02	1731.95	578.32	1731.97	578.33	3	1.4	0.0	12.8	4.9	7.4	0.0	67.0	25.7	17.6	18.3	35.0	29.1	1.0	2.0	1.7	6.3	9.5	14.8	6.4
21					99	GEGQLSAAER	iTRAQ4plex@N-term		0.02	1160.61	581.31	1160.59	581.30	2	149.6	119.5	265.4	266.5	18.7	14.9	33.1	33.3
21					99	VFDAIMNFR	iTRAQ4plex@N-term		0.02	1255.67	628.84	1255.65	628.83	2	97.9	142.8	140.9	144.0	18.6	27.2	26.8	27.4
21					99	YEWDVAEAR	iTRAQ4plex@N-term		0.00	1281.61	641.81	1281.61	641.81	2	83.0	135.8	133.3	252.7	13.7	22.5	22.0	41.8
21					99	VNFTVDQIR	iTRAQ4plex@N-term	cleaved M-V@N-term	-0.04	1234.64	618.33	1234.68	618.35	2	1390.8	849.3	2076.0	1538.9	23.8	14.5	35.5	26.3
21					99	GHVFEESQVAGTPMFVVK	iTRAQ4plex@N-term; iTRAQ4plex(K)@18		0.00	2249.17	563.30	2249.18	563.30	4	51.8	44.5	56.3	45.3	26.2	22.5	28.4	22.9
21					98	CLYASVLTAQPR	iTRAQ4plex@N-term; Pyridylethyl(C)@1		0.00	1569.85	524.29	1569.85	524.29	3	69.7	123.2	148.2	121.8	15.1	26.6	32.0	26.3
22	30.8	IPI:IPI00116074.1	ACO2	aconitate hydratase	99	DINQEVYNFLATAGAK	iTRAQ4plex@N-term; No iTRAQ4plex(K)@16		0.02	1896.99	633.34	1896.97	633.33	3	1.4	6.1	0.0	4.3	11.8	51.5	0.0	36.7	15.9	25.9	28.4	29.9	1.6	1.8	1.9	6.2	9.8	11.5	6.6
22					99	NAVTQEFGPVPDTAR	iTRAQ4plex@N-term		0.03	1744.92	582.65	1744.89	582.64	3	33.7	43.8	66.3	54.7	17.0	22.1	33.4	27.6
22					99	WVVIGDENYGEGSSR	iTRAQ4plex@N-term		0.00	1810.86	604.63	1810.86	604.63	3	11.3	12.0	15.2	16.3	20.7	21.9	27.7	29.7
22					92	DGYAQILR	iTRAQ4plex@N-term; Deamidated(Q)@5		0.07	1079.64	540.83	1079.57	540.79	2	328.2	502.9	949.9	850.1	12.5	19.1	36.1	32.3
22					99	FKLEAPDADELPR	iTRAQ4plex@N-term; iTRAQ4plex(K)@2	missed K-L@2	-0.01	1787.95	596.99	1787.97	597.00	3	155.2	139.7	126.8	144.2	27.4	24.7	22.4	25.5
22					99	GHLDNISNNLLIGAINIENGK	iTRAQ4plex@N-term; iTRAQ4plex(K)@21		0.00	2506.38	627.60	2506.37	627.60	4	21.8	37.9	42.7	39.4	15.4	26.7	30.1	27.8
22					99	VAMQDATAQMAMLQFISSGLPK	iTRAQ4plex@N-term; iTRAQ4plex(K)@22		-0.01	2625.35	876.12	2625.36	876.13	3	34.8	61.9	105.9	47.6	13.9	24.7	42.3	19.0
22					99	NDANPETHAFVTSPEIVTALAIAGTLK	iTRAQ4plex@N-term; iTRAQ4plex(K)@27		-0.02	3067.62	767.91	3067.64	767.92	4	8.6	20.0	17.3	20.9	12.9	29.9	25.9	31.3
22					92	LTIQGLK	iTRAQ4plex@N-term; iTRAQ4plex(K)@7		-0.02	1059.67	530.84	1059.69	530.85	2	587.4	600.1	789.9	677.0	22.1	22.6	29.8	25.5
22					99	QGLLPLTFADPSDYNK	iTRAQ4plex@N-term; Lys->Gln@16		0.02	1921.98	641.67	1921.95	641.66	3	1.4	4.2	9.6	11.5	5.3	15.7	35.9	43.2
23	28.8	IPI:IPI00119618.1	CANX	calnexin precursor	99	APVPTGEVYFADSFDR	iTRAQ4plex@N-term		-0.04	1913.89	638.97	1913.93	638.98	3	17.3	105.5	64.3	80.7	6.5	39.4	24.0	30.1	7.1	40.7	18.8	33.4	5.7	2.6	4.7	4.1	9.3	3.1	11.7
23					90	EIEDPEDR	iTRAQ4plex@N-term		0.00	1145.53	573.77	1145.53	573.77	2	64.2	376.3	211.0	301.8	6.7	39.5	22.1	31.7
23					99	KIPNPDFFEDLEPFK	iTRAQ4plex@N-term; iTRAQ4plex(K)@1; Lys->Gln@15	missed K-I@1	0.09	2123.17	708.73	2123.08	708.70	3	47.3	395.3	129.0	195.9	6.2	51.5	16.8	25.5
23					99	TAELSLDQFHDK	iTRAQ4plex@N-term; iTRAQ4plex(K)@12		0.01	1690.89	564.64	1690.88	564.63	3	81.3	354.0	122.4	136.6	11.7	51.0	17.6	19.7
23					85	WEVDEMKETK	iTRAQ4plex@N-term; iTRAQ4plex(K)@7; Lys->Gln@10		0.06	1581.82	528.28	1581.76	528.26	3	148.5	450.3	189.1	253.7	14.3	43.2	18.2	24.4
23					99	SDASTPPSPK	iTRAQ4plex@N-term; Lys->Gln@10		0.06	1129.60	565.81	1129.54	565.78	2	1.4	34.6	16.4	34.3	1.6	39.9	18.9	39.6
23					99	GSLSGWILSK	iTRAQ4plex@N-term; Lys->Gln@10		0.02	1190.66	596.34	1190.64	596.33	2	76.4	439.0	156.8	440.8	6.9	39.4	14.1	39.6
23					99	DDTDDEIAK	iTRAQ4plex@N-term; Lys->Gln@9		0.06	1164.55	583.28	1164.49	583.25	2	1.4	9.0	7.8	23.7	3.3	21.5	18.6	56.6
24	39.8	IPI:IPI00480560.1	HSPA8	heat shock 70kDa protein 8	99	ARFEELNADLFR	iTRAQ4plex@N-term	missed R-F@2	0.00	1623.85	542.29	1623.85	542.29	3	36.2	67.2	84.3	59.4	14.7	27.2	34.1	24.0	15.6	24.8	29.4	30.2	1.6	1.9	1.9	3.7	7.3	8.6	10.3
24					99	FEELNADLFR	iTRAQ4plex@N-term		0.03	1396.74	699.38	1396.71	699.36	2	99.3	148.4	152.1	266.8	14.9	22.3	22.8	40.0
24					99	TTPSYVAFTDTER	iTRAQ4plex@N-term		0.00	1630.80	544.61	1630.80	544.61	3	27.9	33.2	45.6	77.1	15.2	18.1	24.8	41.9
24					98	VEIIANDQGNR	iTRAQ4plex@N-term		0.03	1371.75	458.26	1371.72	458.25	3	85.8	146.0	120.6	130.2	17.8	30.3	25.0	27.0
24					99	TVTNAVVTVPAYFNDSQR	iTRAQ4plex@N-term; Gln->Arg@17		-0.01	2153.12	718.71	2153.14	718.72	3	20.3	36.6	35.6	21.0	17.9	32.2	31.4	18.5
24					99	SINPDEAVAYGAAVQAAILSGDK	iTRAQ4plex@N-term; iTRAQ4plex(K)@23		0.04	2547.38	850.13	2547.34	850.12	3	13.0	25.8	56.8	24.5	10.8	21.5	47.3	20.4
24					86	LLQDFFNGK	iTRAQ4plex@N-term; iTRAQ4plex(K)@9		0.02	1368.78	457.27	1368.76	457.26	3	27.6	50.1	69.4	37.2	14.9	27.2	37.7	20.2
24					99	NSLESYAFNMK	iTRAQ4plex@N-term; Lys->Gln@11		0.04	1446.69	724.35	1446.66	724.34	2	126.4	148.6	167.0	405.2	14.9	17.5	19.7	47.8
24					99	EIAEAYLGK	iTRAQ4plex@N-term; Lys->Gln@9		0.04	1136.63	569.32	1136.58	569.30	2	120.5	213.5	246.5	242.4	14.6	25.9	30.0	29.5
24					96	LLQDFFNGK	iTRAQ4plex@N-term; Lys->Gln@9		0.03	1224.66	613.34	1224.63	613.32	2	95.4	116.2	245.7	277.3	13.0	15.8	33.4	37.7
24					92	TVTNAVVTVPAYFNDSQR	iTRAQ4plex@N-term; Oxidation(P)@10		0.03	2141.11	714.71	2141.09	714.70	3	7.3	11.5	4.3	5.2	25.7	40.6	15.2	18.5
24					84	CNEIISWLDK	iTRAQ4plex@N-term; Pyridylethyl(C)@1; Lys->Gln@10		0.03	1468.75	490.59	1468.71	490.58	3	29.2	42.3	71.3	83.8	12.9	18.7	31.5	37.0
25	30.1	IPI:IPI00223092.5	HADHA	hydroxyacyl-Coenzyme A dehydrogenase	99	ADMVIEAVFEDLGVK	iTRAQ4plex@N-term; iTRAQ4plex(K)@15		0.02	1923.05	642.02	1923.03	642.02	3	20.1	44.3	37.0	42.8	14.0	30.7	25.7	29.7	22.3	20.2	25.9	31.6	0.9	1.2	1.4	8.5	7.2	8.1	9.9
25					99	DTTASAVAVGLR	iTRAQ4plex@N-term		0.02	1303.74	652.88	1303.72	652.87	2	110.7	105.6	139.3	208.8	19.6	18.7	24.7	37.0
25					99	LTTYAMTVPFVR	iTRAQ4plex@N-term		0.01	1541.85	514.96	1541.84	514.95	3	11.9	5.1	4.1	16.1	32.1	13.8	10.9	43.2
25					99	TIEYLEEVAVNFAK	iTRAQ4plex@N-term; Ala->Val@13; iTRAQ4plex(K)@14		-0.04	1941.03	648.02	1941.07	648.03	3	14.9	6.0	15.8	14.5	29.2	11.7	30.8	28.3
25					99	DSIFSNLIGQLDYK	iTRAQ4plex@N-term; iTRAQ4plex(K)@14		-0.01	1900.01	634.34	1900.02	634.35	3	11.8	12.0	23.2	15.3	18.9	19.3	37.3	24.5
25					99	TIEYLEEVAVNFAK	iTRAQ4plex@N-term; iTRAQ4plex(K)@14		-0.04	1913.00	638.67	1913.04	638.69	3	51.7	48.3	38.9	26.5	31.3	29.2	23.5	16.0
25					99	AGLEQGSDAGYLAESQK	iTRAQ4plex@N-term; Lys->Gln@17		0.05	1866.92	623.31	1866.87	623.30	3	8.8	13.7	22.2	32.7	11.3	17.7	28.7	42.2
26	43.4	IPI:IPI00130280.1	ATP5A1	ATP synthase subunit alpha	99	AVDSLVPIGR	iTRAQ4plex@N-term		0.00	1169.69	585.85	1169.69	585.85	2	1423.8	1833.2	2242.5	2297.2	18.3	23.5	28.8	29.5	16.0	24.1	28.7	31.3	1.5	1.8	2.0	6.5	5.4	4.2	8.2
26					99	FENAFLSHVISQHQSLLGNIR	iTRAQ4plex@N-term		0.04	2553.40	639.36	2553.36	639.35	4	33.8	41.1	57.6	48.5	18.7	22.7	31.8	26.8
26					99	TGAIVDVPVGEELLGR	iTRAQ4plex@N-term		-0.01	1767.97	590.33	1767.99	590.34	3	4.2	12.9	13.2	8.1	11.0	33.5	34.3	21.1
26					99	TGTAEMSSILEER	iTRAQ4plex@N-term		-0.02	1566.75	784.38	1566.77	784.39	2	187.9	302.1	382.5	378.6	15.0	24.1	30.6	30.3
26					90	QMSLLLR	iTRAQ4plex@N-term		-0.01	1003.59	502.80	1003.60	502.81	2	646.5	979.0	1577.1	1864.6	12.8	19.3	31.1	36.8
26					99	TGTAEMSSILEER	iTRAQ4plex@N-term; Dimethyl(R)@13		-0.02	1594.78	532.60	1594.80	532.61	3	5.8	11.2	13.8	22.2	10.9	21.1	26.1	41.8
26					99	FENAFLSHVISQHQSLLGNIR	iTRAQ4plex@N-term; Dimethyl(R)@21		0.00	2581.38	646.35	2581.39	646.35	4	35.4	35.1	53.7	54.1	19.9	19.7	30.2	30.3
26					98	VVDALGNAIDGK	iTRAQ4plex@N-term; iTRAQ4plex(K)@12		0.00	1458.83	487.29	1458.83	487.28	3	12.9	30.0	26.0	18.4	14.8	34.4	29.8	21.1
26					99	GMSLNLEPDNVGVVVFGNDK	iTRAQ4plex@N-term; iTRAQ4plex(K)@20		-0.03	2391.20	798.07	2391.23	798.09	3	111.3	81.0	72.1	77.2	32.6	23.7	21.1	22.6
26					82	VVDALGNAIDGK	iTRAQ4plex@N-term; Lys->Gln@12		0.07	1314.76	658.39	1314.69	658.35	2	163.6	241.2	421.2	570.0	11.7	17.3	30.2	40.8
26					99	NVQAEEMVEFSSGLK	iTRAQ4plex@N-term; Lys->Gln@15		0.06	1810.91	604.65	1810.85	604.62	3	8.6	21.5	17.8	36.1	10.2	25.6	21.2	43.0
27	35.4	IPI:IPI00133522.1	P4HB	protein disulfide-isomerase precursor	99	EADDIVNWLK	iTRAQ4plex@N-term; Lys->Gln@10		0.04	1345.71	673.86	1345.66	673.84	2	48.5	125.1	171.0	231.3	8.4	21.7	29.7	40.2	5.7	22.5	28.4	43.4	3.9	5.0	7.6	2.8	5.3	3.7	6.8
27					99	VDATEESDLAQQYGVR	iTRAQ4plex@N-term		-0.04	1923.89	642.30	1923.93	642.32	3	32.3	109.7	87.2	111.2	9.5	32.2	25.6	32.7
27					87	FFPASADR	iTRAQ4plex@N-term		-0.02	1053.52	527.77	1053.54	527.78	2	1040.4	3193.3	3474.7	4006.2	8.9	27.3	29.7	34.2
27					99	ILEFFGLK	iTRAQ4plex@N-term; Lys->Arg@8		0.03	1137.70	569.86	1137.67	569.84	2	6.9	29.5	86.2	111.3	3.0	12.6	36.8	47.6
27					99	LITLEEEMTK	iTRAQ4plex@N-term; Lys->Gln@10		0.02	1349.71	675.86	1349.69	675.85	2	90.8	332.4	452.7	724.6	5.7	20.8	28.3	45.3
27					99	MDSTANEVEAVK	iTRAQ4plex@N-term; Lys->Gln@12		0.03	1436.69	719.35	1436.66	719.34	2	58.3	301.1	338.7	552.7	4.7	24.1	27.1	44.2
27					99	ILEFFGLK	iTRAQ4plex@N-term; Lys->Gln@8		0.03	1109.65	555.83	1109.62	555.82	2	5.1	42.9	57.3	104.9	2.4	20.4	27.3	49.9
27					97	QLAPIWDK	iTRAQ4plex@N-term; Lys->Gln@8		0.05	1113.64	557.83	1113.59	557.80	2	195.7	637.8	801.6	1248.7	6.8	22.1	27.8	43.3
27					99	VLVGANFEEVAFDEK	iTRAQ4plex@N-term; No iTRAQ4plex(K)@15		0.02	1809.94	604.32	1809.93	604.32	3	1.4	13.1	14.5	32.9	2.3	21.1	23.4	53.2
28	64.3	IPI:IPI00850779.1	GAPDH	glyceraldehyde-3-phosphate dehydrogenase	99	LVINGKPITIFQER	iTRAQ4plex@N-term; iTRAQ4plex(K)@6		-0.04	1915.11	639.38	1915.15	639.39	3	551.2	575.5	753.0	534.5	22.8	23.8	31.2	22.1	16.5	21.3	31.6	30.6	1.3	1.9	1.9	6.0	7.9	4.0	14.4
28					96	WGEAGAEYVVESTGVFTTMEK	iTRAQ4plex@N-term; iTRAQ4plex(K)@21		0.04	2578.29	860.44	2578.25	860.42	3	13.1	19.6	21.4	17.5	18.3	27.3	29.9	24.4
28					99	VIHDNFGIVEGLMTTVHAITATQK	iTRAQ4plex@N-term; iTRAQ4plex(K)@24		0.00	2882.56	577.52	2882.56	577.52	5	7.2	16.9	20.7	8.9	13.3	31.5	38.6	16.5
28					96	QASEGPLK	iTRAQ4plex@N-term; iTRAQ4plex(K)@8		0.01	1116.65	559.33	1116.64	559.33	2	758.7	669.7	1107.5	784.4	22.8	20.2	33.4	23.6
28					97	VIPELNGK	iTRAQ4plex@N-term; Lys->Gln@8		0.02	1012.59	507.30	1012.57	507.29	2	892.6	958.8	1739.4	2776.8	14.0	15.1	27.3	43.6
28					99	VVDLMAYMASK	iTRAQ4plex@N-term; No iTRAQ4plex(K)@11		0.02	1370.73	686.37	1370.71	686.36	2	11.7	15.6	45.5	82.5	7.5	10.0	29.3	53.1
29	43.4	IPI:IPI00850840.1	LRP	ribosomal protein SA	99	AIVAIENPADVSVISSR	iTRAQ4plex@N-term		-0.06	1883.99	629.00	1884.04	629.02	3	11.4	31.0	41.6	43.7	8.9	24.3	32.6	34.3	11.8	33.8	26.9	27.5	2.9	2.3	2.3	5.1	13.5	12.7	9.3
29					99	GAHSVGLMWWMLAR	iTRAQ4plex@N-term		0.02	1757.92	586.98	1757.90	586.97	3	8.7	16.8	22.5	24.0	12.1	23.3	31.3	33.3
29					99	FTPGTFTNQIQAAFR	iTRAQ4plex@N-term		-0.01	1841.95	614.99	1841.95	614.99	3	25.6	69.3	87.0	55.9	10.8	29.1	36.6	23.5
29					94	FAAATGATPIAGR	iTRAQ4plex@N-term		0.03	1346.77	449.93	1346.74	449.92	3	29.2	182.1	28.0	27.9	10.9	68.1	10.5	10.5
29					93	LLVVTDPR	iTRAQ4plex@N-term		-0.02	1055.63	528.82	1055.65	528.83	2	270.0	1038.8	1122.5	692.6	8.6	33.3	35.9	22.2
29					99	GAHSVGLMWWMLAR	iTRAQ4plex@N-term; Oxidation(M)@8		0.01	1773.90	592.31	1773.89	592.30	3	8.8	11.6	0.0	15.2	24.7	32.6	0.0	42.7
29					98	ADHQPLTEASYVNLPTIALCNTDSPLR	iTRAQ4plex@N-term; Pyridylethyl(C)@20		0.07	3187.68	797.93	3187.61	797.91	4	18.4	53.9	64.6	52.5	9.7	28.5	34.1	27.7
29					89	YVDIAIPCNNK	iTRAQ4plex@N-term; Pyridylethyl(C)@8; iTRAQ4plex(K)@11		0.02	1641.90	548.31	1641.88	548.30	3	72.6	198.1	168.8	125.1	12.9	35.1	29.9	22.2
29					88	SGALDVLQMK	Protein Terminal Acetyl@N-term; iTRAQ4plex(K)@10	cleaved M-S@N-term	0.03	1246.70	624.36	1246.67	624.34	2	4.2	15.8	16.5	16.6	7.9	29.7	31.1	31.3
30	43.2	IPI:IPI00830215.1	IGHG1	immunoglobulin gamma-1	99	APQVYTIPPPK	iTRAQ4plex@N-term; Lys->Arg@11		0.01	1381.80	461.61	1381.78	461.60	3	106.5	23.2	45.7	67.4	43.9	9.6	18.8	27.7	28.2	21.6	25.9	24.3	0.8	0.9	0.9	12.1	5.9	5.5	8.5
30					98	SLSHSPG	iTRAQ4plex@N-term	cleaved G-K@C-term	0.02	827.45	414.73	827.43	414.72	2	8.8	11.2	9.1	12.6	21.1	26.8	21.9	30.2
30					94	TTPPSVYPLAPGSAAQTN	iTRAQ4plex@N-term	cleaved N-S@C-term	0.05	1915.03	639.35	1914.98	639.33	3	128.6	115.7	130.9	59.5	29.6	26.6	30.1	13.7
30					99	VNSAAFPAPIEK	iTRAQ4plex@N-term; iTRAQ4plex(K)@12		0.00	1530.87	511.30	1530.86	511.30	3	195.4	92.7	103.7	79.0	41.5	19.7	22.0	16.8
30					97	DVLTITLTPK	iTRAQ4plex@N-term; Lys->Arg@10		0.05	1271.80	636.91	1271.76	636.89	2	60.2	73.7	149.7	143.4	14.1	17.3	35.1	33.6
30					97	DVLTITLTPK	iTRAQ4plex@N-term; Lys->Gln@10		0.02	1243.74	622.88	1243.71	622.86	2	71.1	127.0	164.7	175.3	13.2	23.6	30.6	32.6
30					99	SVSELPIMHQDWLNGK	iTRAQ4plex@N-term; Oxidation(M)@8; iTRAQ4plex(K)@16		0.00	2157.12	720.05	2157.11	720.05	3	239.4	148.8	150.0	79.4	38.8	24.1	24.3	12.9
30					99	SVSELPIMHQDWLNGK	iTRAQ4plex@N-term; Oxidation(M)@8; Lys->Gln@16		0.02	2013.00	672.01	2012.97	672.00	3	92.8	99.5	95.6	106.6	23.5	25.2	24.2	27.0
31	32.0	IPI:IPI00845840.1	PKM2	pyruvate kinase isozymes M1/M2	99	DAVLNAWAEDVDLR	iTRAQ4plex@N-term		-0.04	1729.83	577.62	1729.88	577.63	3	7.0	10.4	6.9	6.2	23.0	34.0	22.7	20.3	15.2	24.4	30.0	30.4	1.6	2.0	2.0	6.0	6.9	7.7	8.5
31					98	LDIDSAPITAR	iTRAQ4plex@N-term		0.03	1314.76	658.39	1314.73	658.37	2	67.1	101.3	111.2	125.2	16.6	25.0	27.5	30.9
31					97	GDLGIEIPAEK	iTRAQ4plex@N-term; Lys->Gln@11		0.02	1284.69	643.35	1284.67	643.34	2	29.2	45.3	71.5	102.0	11.8	18.3	28.8	41.1
31					99	NTGIICTIGPASR	iTRAQ4plex@N-term; Pyridylethyl(C)@6		-0.02	1550.82	517.95	1550.84	517.95	3	226.0	491.8	981.9	698.7	9.4	20.5	40.9	29.1
32	61.7	IPI:IPI00459570.1	TPM3	tropomyosin 3	99	AISDELDHALNDMTSI	iTRAQ4plex@N-term		0.03	1887.93	630.32	1887.90	630.31	3	1.4	1.4	1.8	2.0	21.2	21.2	27.3	30.3	22.3	16.4	33.6	27.7	0.7	1.5	1.2	5.3	8.1	6.1	8.9
32					99	IQLVEEELDR	iTRAQ4plex@N-term		0.03	1386.78	694.40	1386.75	694.38	2	187.7	173.8	294.7	147.1	23.4	21.6	36.7	18.3
32					99	IQVLQQQADDAEER	iTRAQ4plex@N-term		0.02	1785.91	596.31	1785.90	596.31	3	20.4	32.5	26.4	33.6	18.1	28.8	23.4	29.7
32					99	EQAEAEVASLNR	iTRAQ4plex@N-term		0.01	1459.75	487.59	1459.74	487.59	3	26.9	11.9	40.3	36.3	23.3	10.3	35.0	31.5
32					99	KLVIIEGDLER	iTRAQ4plex@N-term; iTRAQ4plex(K)@1	missed K-L@1	-0.02	1571.92	524.98	1571.95	524.99	3	103.7	82.7	199.9	115.4	20.7	16.5	39.8	23.0
32					81	LATALQK	iTRAQ4plex@N-term; iTRAQ4plex(K)@7		0.00	1031.65	516.83	1031.66	516.84	2	3020.3	965.5	3541.4	1655.7	32.9	10.5	38.6	18.0
32					99	MELQEIQLK	iTRAQ4plex@N-term; Lys->Gln@9		0.04	1274.71	638.36	1274.67	638.34	2	110.7	38.5	227.0	287.6	16.7	5.8	34.2	43.3
33	37.5	IPI:IPI00154054.1	ACAT1	acetyl-CoA acetyltransferase, mitochondrial precursor	99	ASKPTLNEVVIVSAIR	iTRAQ4plex@N-term; iTRAQ4plex(K)@3	cleaved Y-A@N-term	0.02	1984.21	662.41	1984.19	662.40	3	93.0	87.9	121.4	96.9	23.3	22.0	30.4	24.3	18.0	20.2	31.4	30.4	1.1	1.7	1.7	6.8	6.5	5.4	9.0
33					99	ENGTITAANASTLNDGAAALVLMTAEAAQR	iTRAQ4plex@N-term		0.15	3088.71	1030.58	3088.56	1030.53	3	16.5	4.2	21.0	15.4	28.9	7.3	36.9	26.9
33					99	EVYMGNVIQGGEGQAPTR	iTRAQ4plex@N-term		0.00	2049.00	684.01	2049.01	684.01	3	24.8	38.2	68.9	83.9	11.5	17.7	32.0	38.9
33					99	GATPYGGVK	iTRAQ4plex@N-term; iTRAQ4plex(K)@9		0.00	1136.65	569.33	1136.64	569.33	2	708.7	540.6	1033.7	911.1	22.2	16.9	32.4	28.5
33					99	FASEITPITISVK	iTRAQ4plex@N-term; Lys->Gln@13		0.06	1548.91	775.46	1548.85	775.43	2	32.3	40.3	46.1	44.4	19.8	24.7	28.3	27.2
33					99	TPIGSFLGSLASQPATK	iTRAQ4plex@N-term; Lys->Gln@17		0.02	1817.98	607.00	1817.96	607.00	3	13.0	24.1	21.6	33.7	14.1	26.0	23.4	36.5
33					99	GATPYGGVK	iTRAQ4plex@N-term; Lys->Gln@9		0.06	992.57	497.29	992.50	497.26	2	71.8	166.4	235.3	377.1	8.4	19.6	27.7	44.3
33					99	QATLGAGLPISTPCTTVNK	iTRAQ4plex@N-term; Pyridylethyl(C)@14; iTRAQ4plex(K)@19		-0.03	2264.22	567.06	2264.24	567.07	4	184.4	323.0	477.9	190.5	15.7	27.5	40.6	16.2
34	39.7	IPI:IPI00307837.5	EEF1A1	elongation factor 1-alpha 1	99	EHALLAYTLGVK	iTRAQ4plex@N-term; iTRAQ4plex(K)@12		-0.02	1601.92	534.98	1601.94	534.99	3	712.1	1363.3	1936.7	804.2	14.8	28.3	40.2	16.7	19.5	24.9	33.7	21.9	1.3	1.7	1.1	6.6	6.7	5.0	6.9
34					99	IGGIGTVPVGR	iTRAQ4plex@N-term		0.00	1168.71	585.36	1168.70	585.36	2	399.3	633.6	526.3	321.6	21.2	33.7	28.0	17.1
34					99	SGDAAIVDMVPGKPMCVESFSDYPPLGR	iTRAQ4plex@N-term; iTRAQ4plex(K)@13; Pyridylethyl(C)@16		0.11	3330.74	833.69	3330.63	833.67	4	26.0	50.2	71.4	49.0	13.2	25.5	36.3	24.9
34					99	VETGVLKPGMVVTFAPVNVTTEVK	iTRAQ4plex@N-term; iTRAQ4plex(K)@7; iTRAQ4plex(K)@24		0.02	2946.70	737.68	2946.68	737.68	4	94.5	92.1	89.8	51.5	28.8	28.1	27.4	15.7
34					99	GNVAGDSK	iTRAQ4plex@N-term; iTRAQ4plex(K)@8		0.04	1034.60	518.31	1034.56	518.29	2	13.4	9.0	18.3	12.2	25.2	17.1	34.6	23.1
34					90	QLIVGVNK	iTRAQ4plex@N-term; Lys->Gln@8		0.04	1013.63	507.82	1013.60	507.81	2	664.9	824.9	1739.7	1636.8	13.7	17.0	35.7	33.6
35	67.9	IPI:IPI00467833.5	TPI1	triosephosphate isomerase	99	ELASQPDVDGFLVGGASLKPEFVDIINAK	iTRAQ4plex@N-term; iTRAQ4plex(K)@19; Lys->Gln@29		0.01	3316.75	830.19	3316.74	830.19	4	14.6	20.3	39.1	26.5	14.5	20.2	38.9	26.4	16.9	28.2	27.4	27.5	1.7	1.6	1.6	2.1	5.8	7.7	9.3
35					99	SNVNDGVAQSTR	iTRAQ4plex@N-term		0.05	1390.75	464.59	1390.69	464.57	3	27.7	42.1	42.9	30.7	19.3	29.3	29.9	21.4
35					96	DLGATWVVLGHSER	iTRAQ4plex@N-term		0.02	1682.90	561.97	1682.89	561.97	3	51.9	77.7	60.6	85.7	18.8	28.2	22.0	31.1
35					99	TATPQQAQEVHEK	iTRAQ4plex@N-term; iTRAQ4plex(K)@13		0.04	1753.96	585.66	1753.92	585.65	3	8.7	16.3	15.4	11.6	16.7	31.4	29.6	22.2
35					99	VVLAYEPVWAIGTGK	iTRAQ4plex@N-term; iTRAQ4plex(K)@15		-0.01	1890.08	631.03	1890.09	631.04	3	20.1	47.4	34.0	22.4	16.2	38.3	27.4	18.1
35					99	ELASQPDVDGFLVGGASLKPEFVDIINAK	iTRAQ4plex@N-term; iTRAQ4plex(K)@19; Lys->Arg@29		0.08	3344.86	837.22	3344.79	837.20	4	11.7	15.6	19.2	17.4	18.3	24.4	30.0	27.2
35					99	VVLAYEPVWAIGTGK	iTRAQ4plex@N-term; Lys->Gln@15		-0.01	1745.94	582.99	1745.95	582.99	3	15.9	28.9	16.1	52.1	14.1	25.6	14.2	46.1
36	40.6	IPI:IPI00515173.1	H2-K1	H2-K1 isoform 2 of H-2 class I histocompatibility antigen	99	EQYYTCHVYHQGLPEPLTLR	iTRAQ4plex@N-term; Pyridylethyl(C)@6		0.02	2695.36	674.85	2695.33	674.84	4	35.5	310.7	105.0	67.0	6.9	60.0	20.3	12.9	3.6	60.9	17.0	18.5	17.0	4.7	5.2	2.0	6.2	2.2	5.3
36					99	FDSDAENPR	iTRAQ4plex@N-term		0.02	1193.57	597.79	1193.54	597.78	2	101.6	5501.3	1103.7	1224.9	1.3	69.4	13.9	15.4
36					99	WMEQVEPEYWER	iTRAQ4plex@N-term		-0.01	1824.82	609.28	1824.83	609.28	3	7.3	152.4	46.1	59.7	2.7	57.4	17.4	22.5
36					99	FISVGYVDDTQFVR	iTRAQ4plex@N-term		-0.02	1788.90	597.31	1788.92	597.31	3	9.8	244.3	59.5	60.3	2.6	65.3	15.9	16.1
36					99	TWTAADMAALITK	iTRAQ4plex@N-term; Lys->Gln@13		0.04	1535.82	512.95	1535.78	512.93	3	1.4	24.0	8.5	12.6	3.0	51.5	18.3	27.2
36					99	MYGCEVGSDWR	iTRAQ4plex@N-term; Pyridylethyl(C)@4		-0.03	1550.65	517.89	1550.68	517.90	3	123.5	1546.2	398.6	425.6	5.0	62.0	16.0	17.1
37	69.7	IPI:IPI00127417.1	NME2	nucleoside diphosphate kinase B	99	EIHLWFKPEELIDYK	iTRAQ4plex@N-term; Lys->Gln@7; iTRAQ4plex(K)@15		0.03	2247.21	562.81	2247.18	562.80	4	9.9	29.7	39.4	37.3	8.5	25.5	33.9	32.1	15.9	28.1	28.3	27.8	1.8	1.8	1.7	6.5	6.7	7.1	11.0
37					99	NIIHGSDSVESAEK	iTRAQ4plex@N-term; iTRAQ4plex(K)@14		0.00	1772.92	591.98	1772.91	591.98	3	106.5	301.4	160.5	238.4	13.2	37.4	19.9	29.5
37					99	TFIAIKPDGVQR	iTRAQ4plex@N-term; iTRAQ4plex(K)@6		-0.02	1631.94	544.99	1631.96	544.99	3	1654.6	1476.0	1712.4	1133.3	27.7	24.7	28.7	19.0
37					99	QHYIDLKDRPFFPGLVK	iTRAQ4plex@N-term; Lys->Asn@7; Lys->Gln@17		0.02	2202.15	551.55	2202.13	551.54	4	73.2	98.6	102.3	240.5	14.2	19.2	19.9	46.7
37					98	GDFCIQVGR	iTRAQ4plex@N-term; Pyridylethyl(C)@4		0.04	1242.67	415.23	1242.63	415.22	3	17.4	33.5	44.9	26.3	14.2	27.5	36.8	21.5
37					99	YMNSGPVVAMVWEGLNVVK	iTRAQ4plex@N-term; Val->Met@7; iTRAQ4plex(K)@19		0.07	2412.29	805.10	2412.22	805.08	3	13.0	25.6	22.6	13.1	17.5	34.4	30.4	17.7
38	87.3	IPI:IPI00263863.8	HSPE1	10 kDa heat shock protein	99	GGIMLPEK	iTRAQ4plex@N-term; iTRAQ4plex(K)@8		-0.03	1131.63	566.82	1131.66	566.84	2	2258.3	2414.8	4319.5	2828.8	19.1	20.4	36.5	23.9	17.3	20.4	32.9	29.4	1.2	1.9	1.7	4.0	5.8	5.6	8.9
38					98	VLQATVVAVGSGGK	iTRAQ4plex@N-term; iTRAQ4plex(K)@14		-0.06	1572.88	525.30	1572.94	525.32	3	51.1	75.5	81.4	55.1	19.4	28.7	31.0	20.9
38					99	VVLDDKDYFLFR	iTRAQ4plex@N-term; iTRAQ4plex(K)@6		0.02	1817.02	606.68	1817.00	606.67	3	190.4	395.3	640.6	366.9	12.0	24.8	40.2	23.0
38					95	VVLDDKDYFLFR	iTRAQ4plex@N-term; iTRAQ4plex(K)@6; Dimethyl(R)@12		0.03	1845.06	616.03	1845.03	616.02	3	117.2	87.1	180.6	107.5	23.8	17.7	36.7	21.8
38					99	VLLPEYGGTK	iTRAQ4plex@N-term; Lys->Gln@10		0.00	1219.66	610.84	1219.66	610.84	2	260.3	227.7	644.1	813.1	13.4	11.7	33.1	41.8
38					85	SGEIEPVSVK	iTRAQ4plex@N-term; Lys->Gln@10		0.03	1187.65	594.83	1187.62	594.82	2	26.3	39.8	40.2	63.5	15.5	23.4	23.7	37.4
38					86	GGIMLPEK	iTRAQ4plex@N-term; Lys->Gln@8		-0.01	987.51	494.76	987.52	494.77	2	1553.7	1400.5	2517.9	3225.1	17.9	16.1	29.0	37.1
39	53.7	IPI:IPI00670985.3	EG668548	EG668548 hypothetical protein	99	FEDENFILK	iTRAQ4plex@N-term; Lys->Gln@9		0.05	1297.68	649.85	1297.63	649.82	2	147.9	337.5	786.2	622.2	7.8	17.8	41.5	32.9	13.6	29.8	36.7	20.0	2.2	2.7	1.5	9.3	11.9	7.1	10.8
39					99	VNPTVFFDITADDEPLGR	iTRAQ4plex@N-term	cleaved M-V@N-term	0.02	2149.10	717.37	2149.08	717.37	3	10.3	12.3	17.3	4.7	23.0	27.7	38.8	10.5
39					94	ALSTGEK	iTRAQ4plex@N-term; iTRAQ4plex(K)@7	cleaved Q-A@N-term	-0.01	992.56	497.29	992.57	497.29	2	495.9	1745.8	833.5	265.9	14.8	52.3	24.9	8.0
39					99	KITISDCGQL	iTRAQ4plex@N-term; Lys->Gln@1; Pyridylethyl(C)@7	missed K-I@1	0.02	1325.70	442.91	1325.68	442.90	3	55.2	166.2	234.3	221.0	8.2	24.6	34.6	32.7
39					99	VSFELFADKVPK	iTRAQ4plex@N-term; Lys->Gln@9; iTRAQ4plex(K)@12	missed K-V@9	0.05	1666.97	556.66	1666.92	556.65	3	345.5	332.9	470.1	205.0	25.5	24.6	34.7	15.1
39					99	IIPGFMCQGGDFTR	iTRAQ4plex@N-term; Pyridylethyl(C)@7		-0.03	1789.85	597.62	1789.88	597.63	3	56.6	870.4	1248.8	562.9	2.1	31.8	45.6	20.6
40	34.9	IPI:IPI00129928.2	FH1	fumarate hydratase	99	IYELAAGGTAVGTGLNTR	iTRAQ4plex@N-term		0.03	1907.05	636.69	1907.02	636.68	3	17.6	24.5	35.7	36.1	15.4	21.5	31.3	31.7	17.0	22.9	33.5	26.6	1.3	2.0	1.6	9.4	7.2	10.1	7.9
40					93	IASAIMK	iTRAQ4plex@N-term; iTRAQ4plex(K)@7		0.00	1020.63	511.32	1020.62	511.32	2	873.5	1144.0	1678.9	1005.6	18.6	24.3	35.7	21.4
41	18.3	IPI:IPI00265291.6	ENPP1	ectonucleotide pyrophosphatase/phosphodiesterase family member	99	AEYLHTWGGLLPVISK	iTRAQ4plex@N-term; iTRAQ4plex(K)@16		0.02	2071.19	691.40	2071.17	691.40	3	43.6	76.5	67.8	42.6	18.9	33.2	29.4	18.5	9.5	37.6	24.2	28.7	4.0	2.6	3.0	7.0	4.7	4.2	10.6
41					99	GQPIWVTANHQEVK	iTRAQ4plex@N-term; iTRAQ4plex(K)@14		0.00	1894.03	632.35	1894.03	632.35	3	87.5	763.8	448.5	524.0	4.8	41.9	24.6	28.7
41					99	TFPNHYSIVTGLYPESHGIIDNK	iTRAQ4plex@N-term; iTRAQ4plex(K)@23		-0.02	2889.47	723.38	2889.49	723.38	4	55.6	216.0	123.7	125.9	10.7	41.4	23.7	24.2
41					99	ELESPAAASLLAPMDLGEEPLEK	iTRAQ4plex@N-term; Lys->Gln@23		0.10	2553.37	852.13	2553.26	852.10	3	1.4	13.4	7.5	17.0	3.6	34.0	19.1	43.3
42	22.4	IPI:IPI00124700.1	TFRC	transferrin receptor protein 1	99	HIFWGSGSHTLSALVENLK	iTRAQ4plex@N-term; Lys->Gln@19		0.03	2239.18	560.80	2239.15	560.79	4	4.8	55.0	19.2	51.1	3.7	42.3	14.7	39.3	6.6	49.4	19.1	24.9	7.5	2.9	3.8	3.1	7.8	4.7	11.5
42					99	SAFSNLFGGEPLSYTR	iTRAQ4plex@N-term		-0.02	1888.93	630.65	1888.94	630.66	3	3.4	53.8	14.5	16.7	3.8	60.9	16.4	18.9
42					89	QLSQNTYTPR	iTRAQ4plex@N-term		-0.01	1350.69	451.24	1350.70	451.24	3	48.0	291.3	113.7	117.6	8.4	51.0	19.9	20.6
42					99	QVDGDNSHVEMK	iTRAQ4plex@N-term; iTRAQ4plex(K)@12		0.00	1645.79	549.61	1645.80	549.61	3	347.9	1619.5	849.4	366.2	10.9	50.9	26.7	11.5
42					99	LDTYEALTQK	iTRAQ4plex@N-term; Lys->Gln@10		0.05	1324.72	663.37	1324.66	663.34	2	44.6	307.4	129.3	251.3	6.1	42.0	17.7	34.3
43	37.7	IPI:IPI00113141.1	CS	citrate synthase	99	ALGVLAQLIWSR	iTRAQ4plex@N-term		-0.03	1469.86	490.96	1469.88	490.97	3	4.9	5.9	4.5	8.5	20.7	24.9	18.8	35.7	17.4	24.1	29.2	29.3	1.4	1.7	1.7	5.0	2.7	8.2	3.8
43					99	TVVGQITVDMMYGGMR	iTRAQ4plex@N-term		-0.03	1900.90	634.64	1900.93	634.65	3	20.3	37.0	68.4	44.8	11.9	21.7	40.1	26.3
43					99	VVPGYGHAVLR	iTRAQ4plex@N-term		-0.04	1310.72	437.91	1310.76	437.93	3	322.3	637.7	889.4	730.5	12.5	24.7	34.5	28.3
43					95	GLVYETSVLDPDEGIR	iTRAQ4plex@N-term		-0.01	1905.97	636.33	1905.98	636.33	3	45.2	68.8	65.6	66.6	18.4	27.9	26.6	27.1
43					98	SMSTDGLMK	iTRAQ4plex@N-term; Lys->Arg@9		0.03	1140.57	571.29	1140.54	571.28	2	277.8	254.9	311.0	345.1	23.4	21.4	26.2	29.0
44	20.9	IPI:IPI00129526.1	HSO90B1	endoplasmin precursor	99	EEEAIQLDGLNASQIR	iTRAQ4plex@N-term		0.00	1928.99	644.00	1928.99	644.00	3	8.8	9.5	14.8	29.7	14.1	15.0	23.6	47.3	7.7	23.4	28.7	40.2	3.0	3.7	5.2	5.8	7.8	5.2	6.4
44					99	GVVDSDDLPLNVSR	iTRAQ4plex@N-term		-0.02	1628.83	815.42	1628.85	815.43	2	119.0	562.8	524.7	645.6	6.4	30.4	28.3	34.9
44					99	LISLTDENALAGNEELTVK	iTRAQ4plex@N-term; Lys->Gln@19		0.01	2173.13	725.39	2173.12	725.38	3	1.4	12.7	17.5	19.8	2.7	24.7	34.1	38.5
45	37.9	IPI:IPI00403810.2	TUBA1C	tubulin alpha	99	AVFVDLEPTVIDEVR	iTRAQ4plex@N-term		0.02	1845.02	616.01	1845.00	616.01	3	45.4	60.9	73.6	91.7	16.7	22.4	27.1	33.7	16.7	24.0	32.3	26.9	1.4	1.9	1.6	4.6	10.4	10.8	10.0
45					80	EDAANNYAR	iTRAQ4plex@N-term; Ala->Val@8		0.07	1194.65	598.33	1194.58	598.29	2	272.4	366.4	334.7	322.5	21.0	28.3	25.8	24.9
45					99	AVFVDLEPTVIDEVR	iTRAQ4plex@N-term; Dimethyl(R)@15		0.00	1873.03	625.35	1873.03	625.35	3	14.9	6.8	33.9	14.9	21.2	9.7	48.1	21.1
45					89	IHFPLATYAPVISAEK	iTRAQ4plex@N-term; iTRAQ4plex(K)@16		0.00	2044.16	512.05	2044.16	512.05	4	21.5	50.1	80.0	33.5	11.6	27.1	43.2	18.1
45					99	TIGGGDDSFNTFFSETGAGK	iTRAQ4plex@N-term; Lys->Arg@20		-0.02	2178.98	727.33	2178.99	727.34	3	4.4	6.8	11.7	17.9	10.7	16.7	28.7	43.9
45					99	TIGGGDDSFNTFFSETGAGK	iTRAQ4plex@N-term; Lys->Gln@20		0.04	2150.99	718.00	2150.95	717.99	3	24.5	51.2	26.8	25.3	19.2	40.0	21.0	19.8
46	27.9	IPI:IPI00123639.1	CALR	calreticulin	99	DMHGDSEYNIMFGPDICGPGTK	iTRAQ4plex@N-term; Pyridylethyl(C)@17; Lys->Gln@22		0.06	2632.18	659.05	2632.12	659.04	4	32.4	72.8	55.5	106.1	12.1	27.3	20.8	39.8	7.2	32.5	24.7	35.6	4.5	3.5	5.0	4.3	4.7	3.5	3.6
46					99	LFPSGLDQK	iTRAQ4plex@N-term; iTRAQ4plex(K)@9		-0.04	1291.70	431.57	1291.74	431.59	3	55.0	456.2	322.5	413.9	4.4	36.6	25.8	33.2
46					87	HEQNIDCGGGYVK	iTRAQ4plex@N-term; Pyridylethyl(C)@7; iTRAQ4plex(K)@13		0.03	1811.92	453.99	1811.89	453.98	4	1.4	9.5	7.8	9.6	4.9	33.6	27.6	33.9
47	24.2	IPI:IPI00132653.1	OXCT1	succinyl-CoA:3-ketoacid-coenzyme A transferase 1	99	AGGAGVPAFYTSTGYGTLVQEGGSPIK	iTRAQ4plex@N-term; Lys->Gln@27		0.01	2728.36	910.46	2728.35	910.46	3	17.5	26.4	32.3	19.8	18.2	27.5	33.6	20.6	19.6	24.7	29.1	26.6	1.3	1.5	1.4	3.9	2.4	4.1	5.8
47					99	MISSYVGENAEFER	iTRAQ4plex@N-term		0.05	1774.89	592.64	1774.83	592.63	3	13.2	18.5	22.4	25.6	16.5	23.2	28.1	32.1
47					99	DGSVAIASKPR	iTRAQ4plex@N-term; iTRAQ4plex(K)@9		0.01	1387.82	463.61	1387.80	463.61	3	116.6	114.5	124.6	131.6	23.9	23.5	25.6	27.0
48	63.3	IPI:IPI00848816.1	CFL	cofilin-1	99	ASGVAVSDGVIK	Protein Terminal Acetyl@N-term; iTRAQ4plex(K)@12	cleaved M-A@N-term	0.00	1287.71	644.86	1287.72	644.87	2	164.1	75.3	133.5	73.5	36.8	16.9	29.9	16.5	20.6	17.3	29.6	32.5	0.8	1.4	1.6	10.0	5.1	5.2	12.3
48					99	NIILEEGKEILVGDVGQTVDDPYTTFVK	iTRAQ4plex@N-term; iTRAQ4plex(K)@8; Lys->Gln@28		0.08	3379.84	845.97	3379.76	845.95	4	20.4	30.3	44.2	43.6	14.8	21.9	31.9	31.5

48					99	YALYDATYETK	iTRAQ4plex@N-term; Lys->Arg@11		0.02	1508.75	755.38	1508.73	755.37	2	99.6	64.8	229.3	232.2	15.9	10.4	36.6	37.1											
48					99	YALYDATYETK	iTRAQ4plex@N-term; Lys->Gln@11		0.03	1480.71	741.36	1480.68	741.35	2	305.7	292.2	342.5	356.0	23.6	22.5	26.4	27.5											
48					99	EILVGDVGQTVDDPYTTFVK	iTRAQ4plex@N-term; Lys->Gln@20		0.18	2339.35	780.79	2339.17	780.73	3	4.4	5.4	8.3	18.0	12.2	14.9	23.0	49.8											
49	37.1	IPI:IPI00118736.3	PPM1B	protein phosphatase 1B2 53 kDa isoform	99	DNDGASDEAEEGGSQGK	iTRAQ4plex@N-term; Lys->Gln@17		0.10	1808.81	603.94	1808.71	603.91	3	3.2	4.3	7.2	8.1	14.0	18.9	31.6	35.5	17.7	26.4	31.7	24.2	1.5	1.8	1.4	4.5	5.9	2.5	8.1
49					99	IQNAGGSVMIQR	iTRAQ4plex@N-term		0.05	1416.81	473.28	1416.76	473.26	3	16.0	25.2	27.6	18.3	18.4	28.9	31.6	21.0											
49					99	GPTEQLVSPEPEVYEIVR	iTRAQ4plex@N-term		0.02	2185.16	729.39	2185.14	729.39	3	5.7	8.7	14.1	13.5	13.6	20.7	33.6	32.1											
49					96	HVIEAVYSR	iTRAQ4plex@N-term		0.01	1216.67	406.57	1216.67	406.56	3	10.3	10.4	12.5	7.5	25.3	25.5	30.7	18.5											
49					85	VNGSLAVSR	iTRAQ4plex@N-term		0.02	1045.62	523.82	1045.60	523.81	2	252.5	381.2	435.3	180.3	20.2	30.5	34.8	14.4											
49					99	GPTEQLVSPEPEVYEIVR	iTRAQ4plex@N-term; Dimethyl(R)@18		-0.01	2213.16	738.73	2213.17	738.73	3	1.4	3.2	2.6	2.2	14.9	34.0	27.7	23.4											
50	46.7	IPI:IPI00128973.1	GAP43	neuromodulin	99	QDEGKEDPEADQEHA	No iTRAQ4plex@N-term; iTRAQ4plex(K)@5		0.06	1840.84	614.62	1840.78	614.60	3	15.8	34.5	60.9	66.7	8.9	19.4	34.2	37.5	7.0	30.5	24.5	38.0	4.3	3.5	5.4	2.1	13.1	8.9	10.2
50					99	EGDGSATTDAAPATSPK	iTRAQ4plex@N-term; Lys->Gln@17		0.01	1718.78	573.94	1718.77	573.93	3	1.4	13.4	6.7	8.4	4.7	44.9	22.3	28.1											
50					99	QADVPAAVTDAAATTPAAEDAATK	iTRAQ4plex@N-term; Lys->Gln@24		0.08	2399.24	800.75	2399.16	800.73	3	5.6	20.3	12.6	36.3	7.5	27.2	16.9	48.5											
50					97	AEDGPAKEEPK	iTRAQ4plex@N-term; Lys->Gln@7; iTRAQ4plex(K)@11		0.11	1457.84	486.95	1457.72	486.92	3	11.4	30.5	14.4	17.8	15.4	41.2	19.4	24.0											
51	20.6	IPI:IPI00123342.4	HYOU1	hypoxia up-regulated protein 1	99	LYQPEYQEVSTEEQR	iTRAQ4plex@N-term		0.02	2041.99	681.67	2041.97	681.66	3	15.8	36.9	36.8	54.7	10.9	25.6	25.5	38.0	10.4	25.3	31.4	32.9	2.4	3.0	3.2	1.9	5.9	12.7	8.8
51					96	EVEEEPGLR	iTRAQ4plex@N-term		0.02	1200.63	601.32	1200.61	601.31	2	61.3	230.5	168.1	281.9	8.3	31.1	22.7	38.0											
51					99	VESVFETLVEDSPEEESTLTK	iTRAQ4plex@N-term; iTRAQ4plex(K)@21		0.04	2655.37	886.13	2655.33	886.12	3	5.8	9.4	22.3	11.0	12.0	19.3	46.0	22.7											
52	50.8	IPI:IPI00421223.3	TPM4	tropomyosin alpha-4 chain	99	AEGDAAALNR	iTRAQ4plex@N-term		0.03	1130.61	566.31	1130.58	566.30	2	232.2	245.5	279.7	268.8	22.6	23.9	27.3	26.2	26.8	21.2	27.6	24.4	0.8	1.0	0.9	10.5	6.8	5.2	8.5
52					99	AQEQLATALQNLEEAEK	iTRAQ4plex@N-term; iTRAQ4plex(K)@17		0.00	2173.15	725.39	2173.15	725.39	3	39.1	35.6	36.2	21.2	29.6	26.9	27.4	16.1											
52					99	AQEQLATALQNLEEAEK	iTRAQ4plex@N-term; Lys->Gln@17		0.01	2029.02	677.35	2029.01	677.34	3	49.2	23.4	56.7	83.2	23.2	11.0	26.7	39.1											
52					99	EENVGLHQTLDQTLNELNCI	iTRAQ4plex@N-term; Pyridylethyl(C)@19		-0.02	2531.22	844.75	2531.24	844.76	3	72.1	84.5	107.5	86.5	20.6	24.1	30.7	24.7											
52					93	AGLNSLEAVK	Protein Terminal Acetyl@N-term; iTRAQ4plex(K)@10	cleaved M-A@N-term	0.02	1186.68	594.35	1186.67	594.34	2	479.9	255.0	324.7	199.1	38.1	20.3	25.8	15.8											
53	27.8	IPI:IPI00116498.1	YWHAZ	14-3-3 protein zeta/delta	99	DICNDVLSLLEK	iTRAQ4plex@N-term; Pyridylethyl(C)@3; iTRAQ4plex(K)@12		0.01	1753.96	585.66	1753.95	585.66	3	6.5	49.0	55.6	31.2	4.6	34.4	39.0	22.0	10.0	21.6	35.4	33.0	2.2	3.5	3.3	6.4	6.8	2.8	10.2
53					99	DSTLIMQLLR	iTRAQ4plex@N-term		0.01	1332.77	667.39	1332.76	667.39	2	39.1	75.5	136.5	145.0	9.9	19.1	34.5	36.6											
53					89	LAEQAER	iTRAQ4plex@N-term		0.01	959.52	480.77	959.52	480.77	2	777.3	856.1	1555.3	977.4	18.7	20.5	37.3	23.5											
53					99	SVTEQGAELSNEER	iTRAQ4plex@N-term; Dimethyl(R)@14		0.01	1719.85	574.29	1719.84	574.29	3	23.5	27.0	45.7	42.5	17.0	19.5	32.9	30.6											
53					99	GIVDQSQQAYQEAFEISK	iTRAQ4plex@N-term; Lys->Arg@18		0.05	2212.14	738.39	2212.09	738.37	3	1.4	5.7	9.6	9.3	5.4	21.9	36.9	35.9											
53					99	GIVDQSQQAYQEAFEISK	iTRAQ4plex@N-term; No iTRAQ4plex(K)@18		0.02	2184.10	729.04	2184.08	729.03	3	1.4	4.2	9.4	14.8	4.7	14.0	31.6	49.6											
54	26.8	IPI:IPI00120793.1	PRNP	prion protein	99	HVAGAAAAGAVVGGLGGYMLGSAMSR	iTRAQ4plex@N-term	cleaved R-P@C-term	0.03	2474.29	825.77	2474.26	825.76	3	19.5	72.8	156.3	32.2	7.0	25.9	55.7	11.5	4.0	20.5	65.1	10.5	5.2	16.4	2.6	2.5	7.1	10.8	2.2
54					96	QHTVTTTTK	Gln->pyro-Glu@N-term; iTRAQ4plex(K)@9		0.02	1142.62	572.32	1142.61	572.31	2	3.7	39.9	101.3	21.2	2.2	24.0	61.0	12.8											
54					99	PMIHFGNDWEDR	iTRAQ4plex@N-term	cleaved R-P@N-term	0.06	1659.82	554.28	1659.76	554.26	3	16.5	72.1	149.1	31.8	6.1	26.8	55.3	11.8											
54					97	ESQAYYDGR	iTRAQ4plex@N-term		0.00	1231.56	616.79	1231.56	616.79	2	7.0	23.7	156.8	17.7	3.4	11.5	76.4	8.6											
54					99	VVEQMCVTQYQK	iTRAQ4plex@N-term; Pyridylethyl(C)@6; iTRAQ4plex(K)@12		-0.09	1847.86	616.96	1847.95	616.99	3	97.7	1250.1	6753.1	678.5	1.1	14.2	76.9	7.7											
55	34.3	IPI:IPI00135686.2	PPIB	peptidylprolyl isomerase B	99	DTNGSQFFITTVK	iTRAQ4plex@N-term; Lys->Gln@13		0.04	1600.83	534.62	1600.79	534.60	3	4.3	11.5	8.8	19.1	9.8	26.3	20.1	43.8	10.3	30.3	24.2	35.1	2.9	2.3	3.4	3.8	10.1	3.0	12.0
55					99	IEVEKPFAIAK	iTRAQ4plex@N-term; iTRAQ4plex(K)@5; Lys->Gln@11		0.05	1531.94	511.65	1531.89	511.64	3	78.6	129.8	156.4	206.9	13.7	22.7	27.4	36.2											
55					98	VVFGLFGK	iTRAQ4plex@N-term; iTRAQ4plex(K)@8		0.01	1153.72	577.87	1153.71	577.86	2	265.7	956.4	514.7	375.9	12.6	45.3	24.4	17.8											
55					99	TVDNFVALATGEK	iTRAQ4plex@N-term; Lys->Gln@13		0.04	1507.81	503.61	1507.76	503.60	3	1.4	7.3	6.8	11.6	5.2	27.0	25.1	42.8											
56	39.7	IPI:IPI00121788.1	PRDX1	peroxiredoxin-1	99	ADEGISFR	iTRAQ4plex@N-term		0.00	1037.52	519.77	1037.53	519.77	2	2415.7	2732.2	5501.9	5346.4	15.1	17.1	34.4	33.4	16.8	16.0	34.1	33.1	0.9	2.0	2.0	5.0	3.0	2.7	10.0
56					99	QITINDLPVGR	iTRAQ4plex@N-term		0.01	1368.80	685.41	1368.78	685.40	2	780.2	792.1	1842.9	1853.8	14.8	15.0	35.0	35.2											
56					98	IGYPAPNFK	iTRAQ4plex@N-term; iTRAQ4plex(K)@9		0.00	1293.73	647.87	1293.73	647.87	2	623.7	500.0	943.8	509.5	24.2	19.4	36.6	19.8											
56					99	TIAQDYGVLK	iTRAQ4plex@N-term; Lys->Gln@10		0.07	1250.73	626.37	1250.66	626.34	2	110.8	104.0	254.4	368.8	13.2	12.4	30.4	44.0											
57	32.6	IPI:IPI00130734.1	TYMS	thymidylate synthase	99	DFLDSLGFSAR	iTRAQ4plex@N-term		0.00	1370.69	686.35	1370.70	686.35	2	130.5	170.9	149.9	88.1	24.2	31.7	27.8	16.3	21.4	21.3	26.6	30.7	1.0	1.2	1.4	5.9	10.2	3.1	13.9
57					99	MLVVGSELQSDAQQLSAEAPR	iTRAQ4plex@N-term		-0.01	2372.20	791.74	2372.21	791.74	3	57.5	59.7	46.3	46.8	27.3	28.4	22.0	22.2											
57					99	MLVVGSELQSDAQQLSAEAPR	iTRAQ4plex@N-term; Ala->Val@19		-0.03	2400.22	801.08	2400.24	801.09	3	11.9	7.7	16.6	22.0	20.4	13.2	28.5	37.9											
57					99	DMDSDYSGQGVDQLQK	iTRAQ4plex@N-term; No iTRAQ4plex(K)@16		0.04	1928.89	643.97	1928.85	643.96	3	7.4	6.5	15.2	25.3	13.6	11.9	27.9	46.5											
58	29.3	IPI:IPI00110805.1	H2-D1	H2-D1 H-2 class I histocompatibility antigen	99	RPEGDVTLR	iTRAQ4plex@N-term		0.00	1185.66	396.23	1185.66	396.23	3	34.5	149.8	109.2	72.3	9.4	41.0	29.9	19.8	3.4	56.7	19.9	20.0	16.7	5.9	5.9	3.5	9.7	5.9	5.5
58					99	WIEQEGPEYWER	iTRAQ4plex@N-term		0.04	1764.87	589.30	1764.82	589.28	3	1.4	126.0	39.1	47.0	0.7	59.0	18.3	22.0											
58					99	YMEVGYVDNTEFVR	iTRAQ4plex@N-term		0.01	1864.88	622.64	1864.88	622.63	3	4.6	134.7	39.2	36.2	2.2	62.7	18.2	16.9											
58					99	TWTAADMAAQITR	iTRAQ4plex@N-term		-0.02	1578.78	527.27	1578.79	527.27	3	1.4	55.6	16.0	11.2	1.7	66.0	19.0	13.3											
58					98	YMEVGYVDNTEFVR	iTRAQ4plex@N-term; Dimethyl(R)@14		0.00	1892.91	631.98	1892.91	631.98	3	3.3	58.3	14.9	29.6	3.1	55.0	14.0	27.9											
59	35.7	IPI:IPI00454042.2	FAM3C	FAM3C	99	GINIALVNGK	iTRAQ4plex@N-term; iTRAQ4plex(K)@10		0.00	1285.79	429.61	1285.80	429.61	3	15.2	202.7	27.5	43.1	5.3	70.3	9.5	14.9	4.7	64.4	12.5	18.5	13.8	2.7	4.0	2.7	7.1	4.1	2.8
59					99	LIAELGSTSITSLGFR	iTRAQ4plex@N-term		2.06	1810.08	604.37	1808.02	603.68	3	1.4	48.2	10.9	16.8	1.8	62.3	14.1	21.8											
59					99	SALDSAIR	iTRAQ4plex@N-term		0.01	975.56	488.79	975.55	488.78	2	131.6	2707.9	339.4	703.5	3.4	69.7	8.7	18.1											
59					99	MASGAANVVGPK	iTRAQ4plex@N-term; iTRAQ4plex(K)@12		-0.03	1388.74	463.92	1388.77	463.93	3	101.3	685.7	216.8	236.6	8.2	55.3	17.5	19.1											
60	24.8	IPI:IPI00625588.1		LOC100046745 hypothetical protein	99	DLDKPFLLPVESVYSIPGR	iTRAQ4plex@N-term; Lys->Gln@4		0.01	2288.23	763.75	2288.22	763.75	3	14.4	32.0	49.0	48.8	10.0	22.2	34.0	33.8	15.2	19.5	33.3	32.0	1.3	2.2	2.1	4.7	2.3	3.8	5.4
60					99	LLDAVDTYIPVPTR	iTRAQ4plex@N-term		-0.03	1715.93	572.98	1715.96	572.99	3	23.6	25.7	41.4	51.3	16.6	18.1	29.2	36.1											
60					99	TVVTGIEMFHK	iTRAQ4plex@N-term; iTRAQ4plex(K)@11		0.02	1548.87	517.30	1548.86	517.29	3	158.0	151.0	302.8	214.1	19.1	18.3	36.7	25.9											
61	25.8	IPI:IPI00454016.5	MAT2A	S-adenosylmethionine synthetase isoform type-2	99	FVIGGPQGDAGLTGR	iTRAQ4plex@N-term		0.02	1587.87	530.30	1587.85	530.29	3	70.7	106.8	74.2	54.3	23.1	34.9	24.3	17.7	19.2	28.3	31.1	21.4	1.5	1.6	1.1	3.1	5.6	4.9	3.3
61					99	TAAYGHFGR	iTRAQ4plex@N-term		0.03	1122.60	375.21	1122.57	375.20	3	129.3	152.9	223.9	131.6	20.3	24.0	35.1	20.6											
61					99	NFDLRPGVIVR	iTRAQ4plex@N-term		0.01	1428.84	477.29	1428.83	477.28	3	114.5	210.5	209.7	148.4	16.8	30.8	30.7	21.7											
61					99	YLDEDTIYHLQPSGR	iTRAQ4plex@N-term		0.00	1949.96	650.99	1949.96	650.99	3	364.3	507.4	742.7	557.8	16.8	23.4	34.2	25.7											
62	13.3	IPI:IPI00271951.5	PDIA4	protein disulfide isomerase associated 4	99	FAMEPEEFDSDTLR	iTRAQ4plex@N-term		0.03	1829.86	610.96	1829.83	610.95	3	16.9	53.2	45.4	46.8	10.4	32.8	28.0	28.8	10.1	25.1	27.0	37.8	2.5	2.7	3.8	1.0	5.3	1.5	6.5
62					99	FDVSGYPTIK	iTRAQ4plex@N-term; Lys->Gln@10		0.06	1269.70	635.86	1269.64	635.83	2	51.3	137.9	145.7	255.0	8.7	23.4	24.7	43.2											
62					99	VDATEQTDLAK	iTRAQ4plex@N-term; Lys->Gln@11		0.05	1333.70	667.86	1333.65	667.83	2	54.8	115.5	135.0	184.0	11.2	23.6	27.6	37.6											
62					99	MDATANDITNDQYK	iTRAQ4plex@N-term; Lys->Gln@14		0.10	1742.85	581.96	1742.75	581.93	3	10.1	21.0	27.9	42.1	10.0	20.8	27.6	41.7											
63	47.6	IPI:IPI00133110.1	TXDC12	thioredoxin domain-containing protein 12 precursor	99	DEDFSPDGGYIPR	iTRAQ4plex@N-term		0.02	1610.75	537.93	1610.73	537.92	3	44.6	26.2	32.7	41.9	30.7	18.0	22.5	28.8	30.7	18.9	25.9	24.6	0.6	0.8	0.8	6.6	3.2	2.7	5.8
63					99	YFYVSAEQVVQGMK	iTRAQ4plex@N-term; iTRAQ4plex(K)@14		0.00	1936.00	646.34	1936.00	646.34	3	236.0	96.8	164.5	153.5	36.3	14.9	25.3	23.6											
63					99	EAAASGLPLMVIIHK	iTRAQ4plex@N-term; iTRAQ4plex(K)@15		-0.02	1837.06	613.36	1837.07	613.37	3	104.8	99.9	143.0	143.4	21.3	20.3	29.1	29.2											
63					99	VRPEIINESGNPSYK	iTRAQ4plex@N-term; iTRAQ4plex(K)@15		-0.05	1990.02	664.35	1990.07	664.36	3	303.2	197.5	234.7	148.4	34.3	22.3	26.6	16.8											
64	51.3	IPI:IPI00230427.5	MIF	macrophage migration inhibitory factor	99	ASVPEGFLSELTQQLAQATGK	iTRAQ4plex@N-term; Lys->Gln@21	cleaved K-P@C-term	0.09	2318.27	773.76	2318.19	773.74	3	7.6	10.2	11.0	12.2	18.5	24.8	26.9	29.8	15.8	25.1	30.0	29.1	1.6	1.9	1.8	5.1	2.3	8.2	5.5
64					98	IVNTNVPR	iTRAQ4plex@N-term	cleaved F-I@N-term	0.02	1055.64	528.83	1055.62	528.82	2	284.1	342.5	356.0	511.6	19.0	22.9	23.8	34.2											
64					99	LLCGLLSDR	iTRAQ4plex@N-term; Pyridylethyl(C)@3		-0.02	1237.68	413.57	1237.70	413.57	3	8.5	23.9	34.1	20.2	9.8	27.6	39.3	23.3											
65	23.3	IPI:IPI00132799.4	C1QBP	complement component 1	99	AEEQEPELTSTPNFVVEVTK	iTRAQ4plex@N-term; Lys->Gln@20		0.03	2390.20	797.74	2390.16	797.73	3	1.4	11.3	21.1	32.7	2.1	17.0	31.7	49.2	9.8	17.4	22.2	50.7	1.8	2.3	5.2	7.7	8.0	9.6	18.6
65					99	AFVEFLTDEIKEEK	iTRAQ4plex@N-term; iTRAQ4plex(K)@11; iTRAQ4plex(K)@14		-0.01	2129.15	710.72	2129.16	710.73	3	107.4	120.6	152.4	142.1	20.6	23.1	29.2	27.2											
65					99	AFVEFLTDEIK	iTRAQ4plex@N-term; Lys->Gln@11		0.06	1454.80	728.41	1454.74	728.38	2	29.6	23.1	50.8	273.1	7.9	6.1	13.5	72.5											
65					98	MSGDWELEVNGTEAK	iTRAQ4plex@N-term; Lys->Gln@15		0.12	1808.92	603.98	1808.80	603.94	3	8.5	23.1	14.2	53.5	8.6	23.3	14.3	53.9											
66	57.8	IPI:IPI00229517.5	LGALS	galectin-1	99	DSNNLCLHFNPR	iTRAQ4plex@N-term; Pyridylethyl(C)@6		0.07	1677.89	420.48	1677.82	420.46	4	1.4	24.9	22.7	13.1	2.3	40.1	36.5	21.1	5.3	32.8	37.4	24.4	6.1	7.0	4.6	3.1	10.7	4.8	8.9
66					86	LNMEAINYMAADGDFK	iTRAQ4plex@N-term; Deamidated(N)@7; Lys->Glu@16		0.09	1947.93	650.32	1947.84	650.29	3	3.9	28.6	38.5	30.9	3.8	28.0	37.8	30.4											
66					99	LNMEAINYMAADGDFK	iTRAQ4plex@N-term; iTRAQ4plex(K)@16		-0.03	2089.97	697.66	2090.01	697.68	3	50.4	456.9	417.6	202.7	4.5	40.5	37.0	18.0											
66					95	SFVLNLGK	iTRAQ4plex@N-term; iTRAQ4plex(K)@8		0.01	1164.72	583.37	1164.71	583.36	2	471.5	1341.5	2190.5	949.1	9.5	27.1	44.2	19.2											
66					99	LNMEAINYMAADGDFK	iTRAQ4plex@N-term; Lys->Arg@16		-0.02	1973.89	658.97	1973.91	658.98	3	5.7	15.0	32.9	25.7	7.2	18.9	41.5	32.4											
66					99	LNMEAINYMAADGDFK	iTRAQ4plex@N-term; Lys->Gln@16		0.03	1945.90	649.64	1945.87	649.63	3	1.4	23.0	32.1	33.3	1.6	25.6	35.8	37.1											
66					97	FNAHGDANTIVCNTK	iTRAQ4plex@N-term; Pyridylethyl(C)@12; iTRAQ4plex(K)@15		0.01	1997.01	500.26	1997.00	500.26	4	10.7	61.6	36.2	15.8	8.6	49.6	29.1	12.7											
67	15.5	IPI:IPI00762897.1	UGCGL1	UDP-glucose ceramide glucosyltransferase-like 1	99	ISMINNPSQEISDSSTPIFR	iTRAQ4plex@N-term		-0.02	2379.17	794.06	2379.19	794.07	3	18.3	59.6	52.3	53.2	10.0	32.5	28.5	29.0	7.4	30.0	32.4	30.1	4.0	4.4	4.1	2.5	4.3	3.6	3.6
67					99	YNSWPSSLQELLRPTFPGVIR	iTRAQ4plex@N-term		0.06	2603.46	868.83	2603.40	868.81	3	23.1	115.2	163.6	156.6	5.0	25.1	35.7	34.1											
67					99	LGIEGLSLHNILK	iTRAQ4plex@N-term; iTRAQ4plex(K)@13		0.01	1694.04	565.69	1694.03	565.69	3	9.6	43.2	43.9	36.1	7.2	32.5	33.1	27.2											
68	27.3	IPI:IPI00462199.1	BSG	basigin	99	SEINVEGPPR	iTRAQ4plex@N-term		0.01	1240.66	621.34	1240.65	621.33	2	197.2	1971.0	592.5	2417.2	3.8	38.1	11.4	46.7	4.1	32.0	13.2	50.7	7.7	3.2	12.2	2.2	11.1	3.7	14.7
68					99	YIVDADDR	iTRAQ4plex@N-term		-0.04	1109.51	555.76	1109.55	555.78	2	220.2	2863.3	868.2	3316.3	3.0	39.4	11.9	45.6											
68					99	VLQEDTLPDLHTK	iTRAQ4plex@N-term; iTRAQ4plex(K)@13		-0.01	1795.98	599.67	1795.99	599.67	3	86.6	457.7	209.4	332.5	8.0	42.1	19.3	30.6											
68					84	YVVVSTPEK	iTRAQ4plex@N-term; Lys->Arg@9		0.05	1192.71	597.36	1192.66	597.34	2	12.1	68.1	54.3	261.0	3.1	17.2	13.7	66.0											
68					97	YVVVSTPEK	iTRAQ4plex@N-term; Lys->Gln@9		0.06	1164.67	583.34	1164.61	583.31	2	24.4	196.2	82.0	549.1	2.9	23.0	9.6	64.5											
69	32.1	IPI:IPI00620531.3	RAP1B	ras-related protein Rap-1b	99	INVNEIFYDLVR	iTRAQ4plex@N-term		-0.04	1637.85	819.93	1637.89	819.95	2	300.0	502.3	253.7	408.5	20.5	34.3	17.3	27.9	27.2	36.3	18.1	18.4	1.3	0.7	0.7	9.5	2.8	1.1	13.4
69					99	SKINVNEIFYDLVR	iTRAQ4plex@N-term; iTRAQ4plex(K)@2	missed K-I@2	0.02	1997.14	666.72	1997.12	666.71	3	41.2	46.5	22.9	10.9	33.9	38.3	18.8	9.0											
70	22.3	IPI:IPI00653598.2	UQCRC1	ubiquinol-cytochrome c reductase 1	99	NNGAGYFLEHLAFK	iTRAQ4plex@N-term; iTRAQ4plex(K)@14		0.02	1868.00	623.67	1867.98	623.67	3	30.3	62.5	59.3	70.0	13.6	28.1	26.7	31.5	17.4	27.2	26.8	28.6	1.6	1.5	1.6	7.1	3.7	4.6	4.1
70					99	TDLTDYLNR	iTRAQ4plex@N-term		0.00	1253.64	627.83	1253.64	627.83	2	148.5	169.9	218.8	238.0	19.2	21.9	28.2	30.7											
70					99	VVELLADIVQNSSLEDSQIEK	iTRAQ4plex@N-term; iTRAQ4plex(K)@21		-0.01	2616.40	655.11	2616.41	655.11	4	7.4	8.4	5.7	6.3	26.5	30.3	20.6	22.5											
70					81	NALVSHLDGTTPVCEDIGR	iTRAQ4plex@N-term; Pyridylethyl(C)@14		-0.02	2245.11	562.29	2245.13	562.29	4	32.7	91.0	100.7	94.3	10.3	28.6	31.6	29.6											
71	15.6	IPI:IPI00420835.3	ITGA6	integrin alpha-6	99	LIATFPDTLTYSAYR	iTRAQ4plex@N-term		-0.01	1874.98	626.00	1874.99	626.00	3	1.4	12.2	10.5	17.4	3.4	29.4	25.3	41.9	8.1	40.2	20.6	31.1	5.0	2.6	3.9	9.2	9.9	4.9	9.3
71					99	EGGLGPLNPLLADVTK	iTRAQ4plex@N-term; Lys->Gln@17		0.05	1850.07	617.70	1850.03	617.68	3	9.1	14.3	10.0	8.3	21.8	34.4	24.0	19.8											
72					99	YTQELTLNR	iTRAQ4plex@N-term		0.04	1280.73	641.37	1280.68	641.35	2	59.8	721.3	261.3	421.2	4.1	49.3	17.9	28.8											
72					99	NIGDINQDGYPDIAVGAPYDDLGK	iTRAQ4plex@N-term; Lys->Gln@24		0.15	2663.39	888.80	2663.25	888.76	3	1.4	22.5	7.1	15.9	3.0	47.9	15.1	33.9											
73	14.2	IPI:IPI00311682.5	ATP1A1	sodium/potassium-transporting ATPase subunit alpha-1	99	DMTSEELDDILR	iTRAQ4plex@N-term		0.04	1579.79	790.90	1579.75	790.88	2	23.5	375.9	115.7	296.1	2.9	46.3	14.3	36.5	6.5	43.3	19.0	31.2	6.7	2.9	4.8	3.1	3.9	4.4	4.7
73					99	LNIPVNQVNPR	iTRAQ4plex@N-term		0.03	1406.84	469.95	1406.81	469.94	3	24.7	114.0	67.4	87.0	8.4	38.9	23.0	29.7											
73					99	GVGIISEGNETVEDIAAR	iTRAQ4plex@N-term		0.01	1973.03	658.68	1973.02	658.68	3	4.0	22.1	9.8	13.7	8.2	44.5	19.8	27.5											
74	18.4	IPI:IPI00132474.3	ITGB1	integrin beta-1	99	LSENNIQTIFAVTEEFQPVYK	iTRAQ4plex@N-term; iTRAQ4plex(K)@21		0.06	2757.50	920.17	2757.45	920.16	3	3.51	37.07	18.59	18.53	4.5	47.7	23.9	23.8	7.1	33.3	28.1	31.6	4.7	4.0	4.5	3.3	9.4	5.3	5.5
74					99	LRPEDITQIQPQQLLLK	iTRAQ4plex@N-term; iTRAQ4plex(K)@17		0.00	2320.34	581.09	2320.37	581.10	4	5.8	14.5	13.79	13.36	12.2	30.6	29.1	28.2											
74					90	DDLENVK	iTRAQ4plex@N-term; Asn->Thr@5; iTRAQ4plex(K)@7		-0.05	1106.56	554.29	1106.61	554.31	2	25.02	82.5	85.49	113.7	8.2	26.9	27.9	37.1											
74					85	GSQTIK	iTRAQ4plex@N-term; iTRAQ4plex(K)@6		0.04	920.59	461.30	920.55	461.28	2	5.64	32.5	20.23	29.27	6.4	37.1	23.1	33.4											
74					95	TVMPYISTTPAK	iTRAQ4plex@N-term; iTRAQ4plex(K)@12		0.01	1595.90	532.97	1595.88	532.97	3	10.5	64.6	96.44	93.69	4.0	24.4	36.4	35.3											
75	37.3	IPI:IPI00115454.3	SFXN1	sideroflexin-1	99	NILLTNEQLENAR	iTRAQ4plex@N-term		0.03	1670.94	557.99	1670.91	557.98	3	46.1	29.8	55.0	59.4	24.2	15.6	28.9	31.2	19.5	19.6	29.5	31.5	1.0	1.5	1.6	6.7	5.6	0.8	0.3
75					99	SSMSVTSLEDELQASIQR	iTRAQ4plex@N-term		-0.03	2124.02	709.01	2124.05	709.02	3	8.7	13.9	17.8	18.7	14.8	23.5	30.0	31.7											
76	12.2	IPI:IPI00752565.1	IGSF8	immunoglobulin superfamily member 8	99	LVAQLDTEGIGSLGPGYEDR	iTRAQ4plex@N-term		-0.02	2233.11	745.38	2233.13	745.39	3	14.4	101.2	64.1	90.4	5.3	37.5	23.7	33.5	4.8	49.0	21.2	25.0	10.1	4.4	5.2	0.5	10.7	4.3	7.4
76					99	VASGDLQVQR	iTRAQ4plex@N-term		0.07	1215.74	608.88	1215.67	608.84	2	44.3	544.4	150.9	189.2	4.8	58.6	16.2	20.4											
76					99	SDMAVEAGAPYAER	iTRAQ4plex@N-term	cleaved H-S@N-term	0.01	1609.77	537.60	1609.75	537.59	3	6.0	69.6	32.5	28.9	4.4	50.8	23.7	21.1											
77	18.2	IPI:IPI00114641.2	SLC3A2	solute carrier family 3, member 2 (CD98)	99	LLLSTDSAR	iTRAQ4plex@N-term		0.05	1118.69	560.35	1118.64	560.33	2	184.6	2416.2	568.8	1052.0	4.4	57.2	13.5	24.9	6.3	46.7	19.7	27.3	7.5	3.1	4.4	2.7	14.8	8.7	3.4
77					99	SLLHGDFHALSSTPDLFSYIR	iTRAQ4plex@N-term; Thr->Ser@13		0.00	2505.28	627.33	2505.28	627.33	4	6.9	30.5	21.8	25.1	8.2	36.3	25.8	29.7											
78	15.2	IPI:IPI00119138.1	UQCRC2	cytochrome b-c1 complex subunit 2	99	LPNGLVIASLENYAPLSR	iTRAQ4plex@N-term		0.01	2070.17	691.06	2070.16	691.06	3	1.4	0.0	4.9	6.3	11.1	0.0	38.8	50.0	17.3	16.3	31.8	34.6	0.9	1.8	2.0	6.3	14.3	8.5	13.3
78					99	SMAASGNLGHTPFLDEL	iTRAQ4plex@N-term		0.04	1902.97	635.33	1902.93	635.32	3	14.7	16.7	13.8	16.7	23.7	27.0	22.3	27.0											
78					96	GGLGLAGAK	iTRAQ4plex@N-term; iTRAQ4plex(K)@9		-0.01	1030.63	516.32	1030.64	516.33	2	982.6	1257.0	1973.4	1545.8	17.1	21.8	34.3	26.8											
79	68.5	IPI:IPI00808222.1	IGKV	immunoglobulin domain variable region	99	DIVLTQSPASLAVSLGQR	iTRAQ4plex@N-term	cleaved G-D@N-term	0.02	1998.15	667.06	1998.12	667.05	3	29.6	25.6	35.0	25.6	25.6	22.1	30.2	22.1	22.3	26.7	27.7	23.3	1.2	1.2	1.0	8.9	8.2	6.3	10.4
79					98	QNGVLNSWTDQDSK	Carbamyl@N-term; iTRAQ4plex(K)@14		-0.01	1777.83	889.92	1777.84	889.92	2	44.7	94.6	146.9	100.2	11.6	24.5	38.0	25.9											
79					98	DSTYSMSSTLTLTKDEYER	Carbamyl@N-term; iTRAQ4plex(K)@14; Dimethyl(R)@19		0.05	2441.19	814.74	2441.14	814.72	3	12.9	29.7	34.8	24.5	12.7	29.1	34.2	24.1											
79					99	LLIYAASNLESGIPAR	iTRAQ4plex@N-term		-0.01	1831.03	611.35	1831.03	611.35	3	48.4	62.8	59.1	48.4	22.1	28.7	27.0	22.1											
79					99	QNGVLNSWTDQDSK	iTRAQ4plex@N-term; Asp->Pro@12; Lys->Gln@14		0.02	1716.84	859.43	1716.82	859.42	2	102.6	61.5	43.7	43.6	40.8	24.5	17.4	17.3											
79					90	QNGVLNSWTDQDSK	iTRAQ4plex@N-term; Deamidated(N)@2; Lys->Gln@14		0.03	1735.80	579.61	1735.78	579.60	3	50.1	124.8	61.3	67.7	16.5	41.1	20.2	22.3											
79					99	DSTYSMSSTLTLTK	iTRAQ4plex@N-term; Dehydrated(T)@3; iTRAQ4plex(K)@14		0.06	1803.97	602.33	1803.92	602.31	3	219.8	148.4	151.0	137.8	33.5	22.6	23.0	21.0											
79					99	DIVLTQSPASLAVSLGQR	iTRAQ4plex@N-term; Dimethyl(R)@18	cleaved G-D@N-term	-0.03	2026.13	676.38	2026.15	676.39	3	10.3	11.0	11.9	10.3	23.7	25.3	27.3	23.8											
79					99	QNGVLNSWTDQDSK	iTRAQ4plex@N-term; Dioxidation(W)@8; Lys->Arg@14		0.05	1794.88	599.30	1794.83	599.28	3	133.5	99.2	112.1	163.2	26.3	19.5	22.1	32.1											
79					95	FSGSGSGTDFTLNI	iTRAQ4plex@N-term; Ile->Gly@14	cleaved I-H@C-term	0.13	1489.81	745.91	1489.68	745.85	2	154.5	242.1	303.2	290.9	15.6	24.4	30.6	29.4											
79					99	DSTYSMSSTLTLTKDEYER	iTRAQ4plex@N-term; iTRAQ4plex(K)@14		-0.04	2514.17	839.06	2514.20	839.08	3	255.2	533.7	410.4	106.4	19.5	40.9	31.4	8.1											
79					99	DSTYSMSSTLTLTKDEYER	iTRAQ4plex@N-term; iTRAQ4plex(K)@14; Dimethyl(R)@19		0.03	2542.27	848.43	2542.24	848.42	3	85.3	169.0	118.3	51.3	20.1	39.9	27.9	12.1											
79					83	LNNFYPK	iTRAQ4plex@N-term; iTRAQ4plex(K)@7	cleaved F-L@N-term	-0.03	1182.63	592.32	1182.66	592.34	2	3630.4	8961.6	8233.4	5890.6	13.6	33.5	30.8	22.0											
79					93	FSGSGSGTDFTLNI	iTRAQ4plex@N-term; Oxidation(N)@13; Ile->Arg@14	cleaved I-H@C-term	0.06	1604.81	535.94	1604.76	535.93	3	88.1	110.0	109.2	60.5	23.9	29.9	29.7	16.5											
79					99	QNGVLNSWTDQDSK	iTRAQ4plex@N-term; Oxidation(W)@8; iTRAQ4plex(K)@14		0.01	1894.94	632.65	1894.93	632.65	3	179.8	119.2	111.6	50.6	39.0	25.8	24.2	11.0											
79					88	ADAAPTVSIFPPSSEQLTSGGASVVCFLNNFYPI	iTRAQ4plex@N-term; Pyridylethyl(C)@26; iTRAQ4plex(K)@34		-0.08	3906.89	977.73	3906.97	977.75	4	51.4	134.8	196.2	87.3	10.9	28.7	41.8	18.6											
79					99	QNGVLNSWTDQDSK	iTRAQ4plex@N-term; Ser->Asp@13; iTRAQ4plex(K)@14		0.01	1906.94	636.65	1906.93	636.65	3	344.3	248.2	265.4	205.7	32.4	23.3	25.0	19.3											
79					98	DSTYSMSSTLTLTK	iTRAQ4plex@N-term; Thr->Ser@13; Lys->Arg@14		0.00	1691.82	564.95	1691.82	564.95	3	26.7	17.8	43.2	75.1	16.4	10.9	26.5	46.1											
79					80	DSTYSMSSTLTLTK	No iTRAQ4plex@N-term; Thr->Glu@13; iTRAQ4plex(K)@14		0.00	1705.82	569.62	1705.82	569.61	3	38.6	26.3	38.8	100.3	18.9	12.9	19.0	49.2											
80	31.4	IPI:IPI00459725.2	IDH3A	isocitrate dehydrogenase [NAD] subunit alpha	99	NVTAIQGPGGK	Carbamyl@N-term; iTRAQ4plex(K)@11		0.04	1227.71	614.86	1227.67	614.84	2	45.6	69.7	34.5	16.9	27.4	41.8	20.7	10.2	16.2	28.9	27.7	27.2	1.8	1.7	1.7	9.8	12.9	7.6	14.8
80					99	TPYTDVNIVTIR	iTRAQ4plex@N-term		0.03	1534.88	512.63	1534.85	512.62	3	8.5	27.3	25.4	33.6	8.9	28.8	26.8	35.5											
80					98	NVTAIQGPGGK	iTRAQ4plex@N-term; iTRAQ4plex(K)@11		0.01	1328.78	443.93	1328.77	443.93	3	5.7	7.4	16.5	16.6	12.3	16.0	35.7	36.0											
81	25.1	IPI:IPI00466570.4	TMED10	transmembrane emp24 domain-containing protein 10	99	DLLVTGAYEITDQSGGAGGLR	iTRAQ4plex@N-term		0.13	2236.28	746.43	2236.15	746.39	3	1.4	7.5	2.8	8.5	6.9	37.0	14.0	42.1	3.5	35.7	16.8	44.0	10.3	4.8	12.7	2.6	10.5	2.0	11.9
81					99	LEDLSESIVNDFAYMK	iTRAQ4plex@N-term; iTRAQ4plex(K)@16		-0.05	2161.03	721.35	2161.09	721.37	3	8.1	182.9	77.2	154.9	1.9	43.2	18.3	36.6											
81					96	IPDQLVILDMK	iTRAQ4plex@N-term; Lys->Gln@11		0.06	1427.84	714.93	1427.78	714.90	2	3.2	59.8	49.9	178.8	1.1	20.5	17.1	61.3											
81					91	LEDLSESIVNDFAYMK	iTRAQ4plex@N-term; Oxidation(M)@15; iTRAQ4plex(K)@16		-0.04	2177.04	726.69	2177.08	726.70	3	1.4	14.9	6.4	12.7	4.0	42.2	18.0	35.9											
82	22.1	IPI:IPI00318614.9	IDH2	isocitrate dehydrogenase [NADP], mitochondrial precursor	99	LILPHVDVQLK	iTRAQ4plex@N-term; iTRAQ4plex(K)@11		0.05	1562.03	521.68	1561.98	521.67	3	73.2	98.1	162.6	168.8	14.6	19.5	32.3	33.6	18.2	22.2	26.0	33.6	1.2	1.4	1.8	5.2	3.8	9.0	0.0
82					92	IIWQFIK	iTRAQ4plex@N-term; iTRAQ4plex(K)@7		-0.01	1234.76	618.39	1234.77	618.39	2	128.0	145.0	114.8	195.7	21.9	24.9	19.7	33.5											
83	28.9	IPI:IPI00330804.4	HSP90AA1	heat shock protein HSP 90-alpha	99	HLEINPDHSIIETLR	iTRAQ4plex@N-term		0.03	1930.07	483.52	1930.04	483.52	4	100.5	161.4	168.0	125.3	18.1	29.1	30.3	22.6	16.8	19.0	32.5	31.7	1.1	1.9	1.9	4.7	8.3	7.9	6.8
83					91	TDTGEPMGR	iTRAQ4plex@N-term		0.07	1106.58	554.30	1106.51	554.26	2	115.3	103.2	112.1	172.4	22.9	20.5	22.3	34.3											
83					84	EDQTEYLEER	iTRAQ4plex@N-term; Dimethyl(R)@10		0.02	1482.71	742.36	1482.70	742.36	2	193.1	129.8	548.8	549.7	13.6	9.1	38.6	38.7											
83					99	ELISNSSDALDK	iTRAQ4plex@N-term; Lys->Gln@12		0.02	1434.71	718.36	1434.70	718.36	2	58.6	78.7	177.5	143.8	12.8	17.2	38.7	31.4											
84	42.7	IPI:IPI00112251.1	TUBB3	tubulin beta-3	99	LATPTYGDLNHLVSATMSGVTTSLR	iTRAQ4plex@N-term		0.04	2748.46	917.16	2748.42	917.15	3	62.2	52.0	60.5	106.1	22.1	18.5	21.5	37.8	16.5	19.0	31.1	33.4	1.1	1.9	2.0	8.0	0.6	13.5	6.1
84					99	LHFFMPGFAPLTAR	iTRAQ4plex@N-term		-0.02	1747.91	583.65	1747.94	583.65	3	49.3	88.2	184.6	132.2	10.9	19.4	40.6	29.1											
85	45.2	IPI:IPI00830581.1	HSD17B10	hydroxyacyl-Coenzyme A dehydrogenase type II	99	GLVAVVTGGASGLGLATAK	iTRAQ4plex@N-term; Lys->Gln@19		0.07	1785.08	596.03	1785.01	596.01	3	5.2	5.8	5.9	6.3	22.4	25.2	25.4	27.0	19.5	27.5	26.7	26.3	1.4	1.4	1.4	6.7	4.3	6.0	10.7
85					99	EQGVLSFWR	iTRAQ4plex@N-term		-0.07	1264.60	633.30	1264.67	633.34	2	1725.3	3649.2	3801.7	6406.3	11.1	23.4	24.4	41.1											
85					83	DFLAGGVAAAISK	iTRAQ4plex@N-term; iTRAQ4plex(K)@13		0.00	1506.86	503.29	1506.86	503.30	3	44.0	54.9	35.8	29.9	26.7	33.4	21.7	18.2											
85					99	VVTIAPGLFATPLLTTLPEK	iTRAQ4plex@N-term; iTRAQ4plex(K)@20		-0.02	2368.40	790.47	2368.42	790.48	3	29.1	45.9	58.3	31.1	17.7	27.9	35.4	18.9											
86	12.8	IPI:IPI00123996.1	NRP1	neuropilin-1	99	NLSALENYNFELVDGVK	iTRAQ4plex@N-term; Lys->Gln@17		0.05	2068.08	690.37	2068.02	690.35	3	1.4	16.3	10.1	14.7	3.3	38.3	23.7	34.6	7.0	49.3	19.6	24.1	7.0	2.8	3.4	4.9	15.3	5.7	8.4
86					99	YDRLEIWDGFPEVGPHIGR	iTRAQ4plex@N-term	missed R-L@3	0.01	2399.22	600.81	2399.21	600.81	4	8.0	50.2	22.1	24.0	7.6	48.2	21.2	23.0											
86					99	NLSALENYNFELVDGVK	iTRAQ4plex@N-term; iTRAQ4plex(K)@17		-0.02	2212.15	738.39	2212.16	738.39	3	12.5	260.0	41.0	51.2	3.4	71.3	11.2	14.0											
86					84	CEWLIQAPEPYQR	iTRAQ4plex@N-term; Pyridylethyl(C)@1		0.03	1880.97	628.00	1880.94	627.99	3	90.9	259.9	145.5	161.8	13.8	39.5	22.1	24.6											
87	69.4	IPI:IPI00226455.1	SPRN	shadoo	99	ICLLLGGTLGALELLRP	iTRAQ4plex@N-term; Pyridylethyl(C)@2		-0.01	2000.19	667.74	2000.20	667.74	3	0.0	68.0	320.8	600.4	0.0	6.9	32.4	60.7	2.9	11.4	20.5	65.2	4.0	7.1	22.7	3.7	6.5	6.5	5.2
87					99	VAAAGAAAGAAAGVAAGLATGSGWR	iTRAQ4plex@N-term		-0.04	2198.13	733.72	2198.17	733.73	3	0.0	21.6	18.3	60.1	0.0	21.6	18.3	60.1											
87					98	YGSSLR	iTRAQ4plex@N-term		0.00	825.45	413.73	825.45	413.73	2	1.2	3.4	34.8	110.7	0.8	2.3	23.2	73.8											
87					99	VAAAGAAAGAAAGVAAGLATGSGWR	iTRAQ4plex@N-term; Oxidation(W)@24		-0.01	2214.15	739.06	2214.16	739.06	3	10.2	17.1	19.1	80.7	8.0	13.4	15.0	63.5											
87					99	VAAAGAAAGAAAGVAAGLATGSGWR	iTRAQ4plex@N-term; Val->Asn@14		0.05	2213.19	738.74	2213.14	738.72	3	12.3	22.4	26.8	112.6	7.1	12.9	15.4	64.7											
87					99	VAAAGAAAGAAAGVAAGLATGSGWR	iTRAQ4plex@N-term; Val->Met@14		0.07	2230.21	744.41	2230.14	744.39	3	1.4	11.4	19.2	70.3	1.4	11.1	18.8	68.7											
88	9.1	IPI:IPI00314069.1	ACOT9	acyl-coenzyme A thioesterase 9	99	NIIHELFLTTLDPK	iTRAQ4plex@N-term; iTRAQ4plex(K)@14		0.02	1941.13	648.05	1941.12	648.05	3	13.1	21.8	20.9	22.1	16.8	28.0	26.8	28.4	16.0	29.2	26.6	28.3	1.8	1.7	1.8	1.2	1.7	0.4	0.1
88					81	VAPSSEER	iTRAQ4plex@N-term		0.03	1017.55	509.78	1017.52	509.77	2	11.2	22.4	19.4	20.8	15.2	30.4	26.3	28.2											
89	18.8	IPI:IPI00416577.1	GDI2	rab GDP dissociation inhibitor beta	99	DLGTDSQIFISR	iTRAQ4plex@N-term		0.02	1494.80	499.27	1494.78	499.27	3	7.0	24.4	24.7	24.9	8.7	30.1	30.5	30.8	6.3	32.8	28.0	33.0	5.2	4.5	5.3	3.4	3.8	3.5	3.1
89					99	FDLGQDVIDFTGHSLALYR	iTRAQ4plex@N-term		0.01	2310.18	771.07	2310.18	771.07	3	8.8	80.5	57.9	79.8	3.9	35.5	25.5	35.1											
90	31.3	IPI:IPI00132950.1	RPS21	40S ribosomal protein S21	99	MGESDDSILR	iTRAQ4plex@N-term		0.04	1265.65	633.83	1265.60	633.81	2	372.9	598.9	1040.5	978.3	12.5	20.0	34.8	32.7	8.6	18.7	37.9	34.9	2.2	4.4	4.1	5.5	1.9	4.3	3.0
90					99	TYGICGAIR	iTRAQ4plex@N-term; Pyridylethyl(C)@5		-0.02	1201.62	401.55	1201.64	401.55	3	153.5	570.0	1340.9	1213.3	4.7	17.4	40.9	37.0											
91	31.5	IPI:IPI00134621.3	RAN	ran GTP-binding nuclear protein	99	NLQYYDISAK	iTRAQ4plex@N-term; Lys->Gln@10		0.09	1357.75	679.88	1357.66	679.84	2	76.7	76.2	169.7	255.2	13.3	13.2	29.4	44.2	18.6	18.0	31.5	31.9	1.0	1.7	1.7	7.6	6.8	3.0	17.3
91					99	SNYNFEKPFLWLAR	iTRAQ4plex@N-term; iTRAQ4plex(K)@7		0.04	2072.15	691.72	2072.11	691.71	3	302.8	287.2	424.9	248.0	24.0	22.7	33.6	19.6											
92	25.6	IPI:IPI00854971.1	PDIA6	protein disulfide isomerase associated 6	99	LAAVDATVNQVLASR	iTRAQ4plex@N-term		0.01	1670.95	557.99	1670.94	557.99	3	5.4	30.5	26.5	33.5	5.6	31.8	27.7	35.0	7.8	29.5	27.9	34.9	3.8	3.6	4.5	3.1	3.2	0.3	0.1
92					99	LYSSSDDVIELTPSNFNR	iTRAQ4plex@N-term	cleaved G-L@N-term	-0.03	2200.05	734.36	2200.08	734.37	3	10.0	27.1	27.9	34.6	10.0	27.2	28.1	34.7											
93	13.7	IPI:IPI00124221.1	ATP1B3	sodium/potassium-transporting ATPase subunit beta-3	99	IIDLIPDGYPQISCLPK	iTRAQ4plex@N-term; Pyridylethyl(C)@14; iTRAQ4plex(K)@17		0.01	2277.28	570.33	2277.27	570.32	4	5.1	42.9	32.5	36.9	4.4	36.5	27.7	31.4	5.1	33.9	28.3	32.7	6.6	5.5	6.4	1.1	3.7	0.9	1.7
93					99	YFPYYGK	iTRAQ4plex@N-term; Lys->Gln@7		0.04	1080.54	541.28	1080.50	541.26	2	4.7623	25.389	23.5	27.519	5.9	31.3	29.0	33.9											
94	57.1	IPI:IPI00850107.1	IGK-V	Igk-V Ig kappa	99	VEAEDVGVYYCANLQELPYTFGGGTK	No iTRAQ4plex@N-term; Pyridylethyl(C)@11; Deamidated(N)@13; Lys->Gln@26		0.01	2928.32	733.09	2928.32	733.09	4	141.2	89.1	127.1	109.6	30.2	19.1	27.2	23.5	31.4	16.3	27.3	25.1	0.5	0.9	0.8	5.0	2.4	0.2	5.4
94					99	FSGSGSGTDFTLRI	iTRAQ4plex@N-term; Carbamyl(R)@13; Ile->Thr@14	cleaved I-S@C-term; missed R-I@13	0.09	1618.86	540.63	1618.77	540.60	3	1077.0	441.2	804.8	602.9	36.8	15.1	27.5	20.6											
94					99	FSGSGSGTDFTLR	iTRAQ4plex@N-term; Leu->Val@12		0.04	1460.74	731.38	1460.70	731.36	2	244.3	132.4	245.6	281.2	27.0	14.7	27.2	31.1											
95	14.5	IPI:IPI00120245.1	ITGAV	integrin alpha-V precursor	99	GLVYIFNGR	iTRAQ4plex@N-term		0.01	1181.68	591.85	1181.67	591.84	2	24.1	142.4	80.6	104.8	6.8	40.5	22.9	29.8	9.0	37.4	23.5	30.1	4.1	2.6	3.3	3.1	4.3	0.8	0.4
95					99	NGYPDLVVGAFGVDR	iTRAQ4plex@N-term		-0.04	1721.84	574.96	1721.89	574.97	3	4.2	13.0	9.1	11.5	11.2	34.4	24.0	30.4											
96	39.9	IPI:IPI00762203.2	FTL1	ferritin light chain 1	99	NLNQALLDLHALGSAR	iTRAQ4plex@N-term		0.01	1849.04	617.35	1849.03	617.35	3	32.8	87.9	64.2	50.3	13.9	37.4	27.3	21.4	10.4	36.9	29.4	23.3	3.5	2.8	2.2	6.0	4.7	4.8	5.1
96					99	TQEAMEAALAMEK	iTRAQ4plex@N-term; Lys->Arg@13		0.05	1593.81	532.28	1593.76	532.26	3	4.3	9.9	10.8	6.0	13.9	31.9	34.9	19.4											
96					99	TQEAMEAALAMEK	iTRAQ4plex@N-term; Lys->Gln@13		0.04	1565.76	522.93	1565.72	522.91	3	1.4	16.3	10.2	11.5	3.6	41.3	26.0	29.1											
97	7.3	IPI:IPI00469000.4	SLC39A6	zinc transporter SLC39A6	99	ESASSSEVTSAVYNAVSEGTR	iTRAQ4plex@N-term		0.01	2274.08	759.03	2274.07	759.03	3	2.3	18.3	22.5	19.5	3.7	29.2	35.9	31.2	3.2	29.9	32.8	34.2	9.4	10.3	10.7	0.8	1.1	2.8	2.8
97					95	AGMTVK	iTRAQ4plex@N-term; Dehydrated(T)@4; iTRAQ4plex(K)@6		-0.03	875.48	438.75	875.51	438.76	2	8.8	122.5	125.4	136.7	2.2	31.1	31.9	34.8											
97					99	YDSQLSSNEEK	iTRAQ4plex@N-term; Lys->Gln@11		0.06	1442.69	722.35	1442.63	722.32	2	18.0	143.7	150.2	180.4	3.7	29.2	30.5	36.6											
98	18.9	IPI:IPI00187289.2	C1ORF75	transmembrane protein C1orf75 homolog	99	EVDRYDAPGIAFYPGQAQLLSCK	iTRAQ4plex@N-term; Pyridylethyl(C)@22; iTRAQ4plex(K)@23	missed R-Y@4	0.03	2933.53	734.39	2933.50	734.38	4	16.2	89.0	57.0	213.2	4.3	23.7	15.2	56.8	3.5	18.2	11.1	67.2	5.1	3.1	19.0	1.1	7.8	5.8	14.7
98					99	QETSVVNYIDQRPAAER	iTRAQ4plex@N-term		0.06	2119.14	707.39	2119.08	707.37	3	17.8	81.3	45.1	500.8	2.8	12.6	7.0	77.6											
99	5.4	IPI:IPI00458003.1	ENPP3	ectonucleotide pyrophosphatase/phosphodiesterase family member	99	ALQSVDNAFGMLMEGLK	iTRAQ4plex@N-term; iTRAQ4plex(K)@17		-0.02	2111.08	704.70	2111.10	704.71	3	1.4	19.3	10.1	11.5	3.3	45.7	23.8	27.1	5.9	43.4	25.1	25.5	7.3	4.2	4.3	3.7	3.2	1.7	2.2
99					99	STQIWTCNLFR	iTRAQ4plex@N-term; Pyridylethyl(C)@7		0.00	1616.83	539.95	1616.83	539.95	3	35.5	170.9	109.3	99.5	8.6	41.2	26.3	24.0											
100	13.5	IPI:IPI00652902.1	GNAI2	guanine nucleotide binding protein, alpha inhibiting 2	99	EYQLNDSAAYYLNDLER	iTRAQ4plex@N-term		0.12	2220.16	741.06	2220.05	741.02	3	1.4	12.0	3.9	13.9	4.5	38.5	12.4	44.6	5.1	35.8	24.3	34.8	7.0	4.7	6.8	0.9	3.8	16.8	13.9
100					99	IAQSDYIPTQQDVLR	iTRAQ4plex@N-term		0.00	1890.00	631.01	1890.00	631.01	3	4.0	23.0	25.2	17.4	5.8	33.1	36.2	24.9											
101	26.9	IPI:IPI00857709.1	TMED2	emp24 domain-containing protein 2	99	GQDMETEAHQNKLEEMINELAVAMTAVK	iTRAQ4plex@N-term; iTRAQ4plex(K)@12; iTRAQ4plex(K)@28	missed K-L@12	0.00	3561.78	713.36	3561.78	713.36	5	4.2	22.4	20.2	27.2	5.7	30.3	27.3	36.8	6.0	33.6	26.1	34.4	5.6	4.4	5.8	0.4	4.7	1.7	3.4
101					99	LEEMINELAVAMTAVK	iTRAQ4plex@N-term; iTRAQ4plex(K)@16		-0.01	2049.10	684.04	2049.11	684.04	3	6.5	38.4	25.9	33.4	6.2	36.9	24.9	32.0											
102	25.9	IPI:IPI00125460.1	ATP5J	ATP synthase-coupling factor 6	99	FEVIDKPQS	iTRAQ4plex@N-term; Lys->Gln@6		0.04	1205.65	603.83	1205.61	603.81	2	124.4	101.8	170.8	130.4	23.6	19.3	32.4	24.7	20.8	18.2	32.5	28.4	0.9	1.6	1.4	3.9	1.5	0.1	5.3
102					99	QASGGPVDIGPEYQQDLDR	iTRAQ4plex@N-term		0.00	2188.05	730.36	2188.05	730.36	3	28.1	26.5	50.5	49.9	18.1	17.1	32.6	32.2											
103	15.3	IPI:IPI00115546.4	GNAO1	guanine nucleotide-binding protein G(o) subunit alpha 2	99	LWGDSGIQECFNR	iTRAQ4plex@N-term; Pyridylethyl(C)@10		0.01	1772.85	591.96	1772.84	591.95	3	49.5	222.2	171.0	159.8	8.2	36.9	28.4	26.5	10.1	32.6	27.3	29.9	3.2	2.7	3.0	2.7	6.0	1.5	4.8
103					98	YYLDSLDR	iTRAQ4plex@N-term		0.02	1187.61	594.81	1187.59	594.80	2	271.0	640.1	592.1	752.3	12.0	28.4	26.3	33.4											
104	4.3	IPI:IPI00273801.3	SLC39A10	zinc transporter ZIP10	99	QSTEESTIGR	iTRAQ4plex@N-term		0.03	1250.65	626.33	1250.62	626.32	2	27.6	466.4	159.6	361.4	2.3	39.3	15.7	42.7	2.4	36.3	16.7	44.6	15.0	6.9	18.4	0.2	4.3	1.4	2.7
104					98	NYLGVEEEK	iTRAQ4plex@N-term; Lys->Gln@9		0.07	1223.65	612.83	1223.58	612.80	2	19.2	250.4	133.4	350.7	2.5	33.2	17.7	46.5											
105	9.2	IPI:IPI00672508.1	ACOT7	acyl coenzyme A thioester hydrolase	99	VLEVPPIVYLR	iTRAQ4plex@N-term		-0.04	1440.84	721.43	1440.88	721.45	2	198.6	152.4	314.1	293.8	20.7	15.9	32.8	30.6	20.1	18.2	28.4	33.4	0.9	1.4	1.7	7.6	11.8	10.1	10.1
105					97	ATLWYVPLSLK	iTRAQ4plex@N-term; Lys->Gln@11		0.03	1433.84	717.92	1433.80	717.91	2	14.9	9.5	43.4	54.5	12.2	7.7	35.5	44.6											
105					97	VVMTQTPLSLPVSLGDQASISCR	iTRAQ4plex@N-term; Thr->Asn@6; Pyridylethyl(C)@22	cleaved D-V@N-term	-0.02	2663.37	888.80	2663.39	888.80	3	16.2	18.3	9.9	14.7	27.4	30.9	16.8	24.9											
106	27.0	IPI:IPI00230682.6	YWHAB	14-3-3 protein alpha/beta	99	QTTVSNSQQAYQEAFEISK	iTRAQ4plex@N-term; No iTRAQ4plex(K)@19		0.04	2302.16	768.39	2302.12	768.38	3	1.4	9.0	2.9	10.7	5.8	37.5	12.0	44.6	10.5	26.3	23.4	39.8	2.5	2.2	3.8	6.5	15.8	16.1	6.8
106					97	YDDMAAAMK	iTRAQ4plex@N-term; Lys->Gln@9		0.04	1158.52	580.27	1158.48	580.25	2	82.6	82.8	190.8	191.6	15.1	15.1	34.8	35.0											
107	19.9	IPI:IPI00129519.3	BASP1	brain acid soluble protein 1	99	AGEASAESTGAADGAAPEEGEAK	iTRAQ4plex@N-term; No iTRAQ4plex(K)@23		0.05	2219.04	740.69	2218.99	740.67	3	1.4	7.3	7.2	5.1	6.7	34.9	34.1	24.4	8.2	18.8	37.4	35.5	2.3	4.5	4.3	5.2	14.9	9.9	8.1
107					99	AVLVDLEPGTMDSVR	iTRAQ4plex@N-term		0.02	1744.94	582.65	1744.92	582.65	3	20.7	19.9	37.8	51.5	15.9	15.3	29.1	39.6											
107					98	NSSYFVEWIPNNVK	iTRAQ4plex@N-term; Lys->Gln@14		0.08	1839.97	614.33	1839.89	614.30	3	1.4	6.9	9.5	9.7	5.1	25.0	34.6	35.3											
107					99	MSATFIGNSTAIQELFK	iTRAQ4plex@N-term; Lys->Gln@17		0.02	2001.02	668.01	2001.00	668.01	3	1.4	0.0	13.7	11.3	5.3	0.0	51.9	42.8											
108	9.7	IPI:IPI00845689.1	TMED9	transmembrane emp24 protein transport domain containing 9	99	QLVEQVEQIQK	iTRAQ4plex@N-term; Lys->Gln@11		0.08	1484.87	495.97	1484.80	495.94	3	1.4	22.5	5.3	50.7	1.8	28.2	6.6	63.5	7.2	44.2	26.1	22.5	6.2	3.6	3.1	6.9	13.1	12.8	19.6
108					99	FSGSGSGTDFTLK	Carbamyl@N-term; iTRAQ4plex(K)@13		-0.08	1489.64	745.82	1489.72	745.87	2	303.0	2236.6	2065.5	2016.3	4.6	33.8	31.2	30.5											
108					99	DSTYSMSSTLTLTKDEYER	Carbamyl@N-term; iTRAQ4plex(K)@14		0.00	2413.11	805.38	2413.11	805.38	3	1.4	32.3	30.4	7.4	2.0	45.1	42.6	10.4											
108					99	FSGVPDRFSGSGSGTDFTLK	iTRAQ4plex@N-term; iTRAQ4plex(K)@20	missed R-F@7	0.05	2349.23	588.32	2349.18	588.30	4	57.7	118.1	145.7	49.3	15.6	31.9	39.3	13.3											
108					99	DSTYSMSSTLTLTKDEYER	iTRAQ4plex@N-term; Oxidation(M)@6; iTRAQ4plex(K)@14		0.07	2530.27	844.43	2530.20	844.41	3	188.2	566.0	234.4	64.7	17.9	53.7	22.3	6.1											
108					99	HNSYTCEATHK	iTRAQ4plex@N-term; Pyridylethyl(C)@6; iTRAQ4plex(K)@11		0.03	1682.84	421.72	1682.81	421.71	4	4.9	260.4	120.9	87.0	1.0	55.0	25.5	18.4											
108					99	FSGSGSGTDFTLK	No iTRAQ4plex@N-term; Ser->Val@2; iTRAQ4plex(K)@13		-0.03	1458.72	730.37	1458.75	730.38	2	412.3	3390.4	821.3	860.1	7.5	61.8	15.0	15.7											
109	20.5	IPI:IPI00762435.2	FYN	fyn Isoform 1	97	IADFGLAR	iTRAQ4plex@N-term		0.02	1005.59	503.80	1005.57	503.79	2	179.9	182.9	224.1	222.5	22.2	22.6	27.7	27.5	13.3	24.2	28.6	33.9	1.8	2.1	2.5	9.1	13.4	10.8	6.0
109					99	LLQDFFNGR	iTRAQ4plex@N-term		0.04	1252.70	627.36	1252.67	627.34	2	11.8	10.0	34.3	30.0	13.8	11.7	39.8	34.8											
109					99	IINEPTAAAIAYGLDR	iTRAQ4plex@N-term		0.03	1831.03	611.35	1831.00	611.34	3	1.4	13.4	6.4	13.8	4.0	38.3	18.2	39.4											
110	10.1	IPI:IPI00118569.1	GNA13	guanine nucleotide-binding protein alpha-13 subunit	99	LGVPDYIPSQQDILLAR	iTRAQ4plex@N-term		0.01	2041.14	681.39	2041.13	681.38	3	5.7	17.3	14.1	10.2	12.0	36.5	29.9	21.6	14.6	35.1	32.8	17.6	2.4	2.3	1.2	3.7	2.0	4.1	5.7
110					99	VKEGMNIVEAMER	iTRAQ4plex@N-term; iTRAQ4plex(K)@2		-0.03	1792.92	598.65	1792.94	598.65	3	199.5	391.4	415.3	157.3	17.1	33.6	35.7	13.5											
111	23.9	IPI:IPI00230707.6	YWHAG	14-3-3 protein gamma	99	NVTELNEPLSNEER	iTRAQ4plex@N-term		0.04	1786.92	596.65	1786.88	596.63	3	5.9	4.3	35.2	17.0	9.5	6.9	56.4	27.2	9.8	12.0	48.7	29.6	1.2	5.0	3.0	0.4	7.1	11.0	3.5
111					97	ETAEADVASLNR	iTRAQ4plex@N-term		0.01	1418.72	473.91	1418.71	473.91	3	8.7	14.7	35.4	27.8	10.1	17.0	40.9	32.0											
112	3.5	IPI:IPI00831568.1	L1CAM	L1 cell adhesion molecule	94	LVVFPTDDISLK	iTRAQ4plex@N-term; Lys->Gln@12		0.06	1489.87	745.94	1489.81	745.91	2	12.7	62.3	49.4	59.5	6.9	33.9	26.9	32.4	10.9	30.9	26.7	31.5	2.8	2.4	2.9	4.3	3.2	2.2	1.9
112					99	WFSLGK	iTRAQ4plex@N-term; iTRAQ4plex(K)@6		0.01	1024.60	513.31	1024.59	513.30	2	6.5	11.607	10.3	13.85	15.4	27.5	24.4	32.8											
112					89	VEGESK	iTRAQ4plex@N-term; iTRAQ4plex(K)@6	cleaved P-V@N-term	0.01	935.53	468.77	935.52	468.77	2	2.1	6.3	5.8	5.9	10.4	31.3	28.9	29.4											
